# Mechanisms by which PE21, an extract from the white willow *Salix alba*, delays chronological aging in budding yeast

**DOI:** 10.18632/oncotarget.27209

**Published:** 2019-10-08

**Authors:** Younes Medkour, Karamat Mohammad, Anthony Arlia-Ciommo, Veronika Svistkova, Pamela Dakik, Darya Mitrofanova, Monica Enith Lozano Rodriguez, Jennifer Anne Baratang Junio, Tarek Taifour, Paola Escudero, Fani-Fay Goltsios, Sahar Soodbakhsh, Hana Maalaoui, Éric Simard, Vladimir I. Titorenko

**Affiliations:** ^1^ Department of Biology, Concordia University, Montreal, Quebec H4B 1R6, Canada; ^2^ Idunn Technologies Inc., Rosemere, Quebec J7A 4A5, Canada

**Keywords:** cellular aging, geroprotectors, lipid metabolism, necrotic cell death, mitochondria

## Abstract

We have recently found that PE21, an extract from the white willow *Salix alba*, slows chronological aging and prolongs longevity of the yeast *Saccharomyces cerevisiae* more efficiently than any of the previously known pharmacological interventions. Here, we investigated mechanisms through which PE21 delays yeast chronological aging and extends yeast longevity. We show that PE21 causes a remodeling of lipid metabolism in chronologically aging yeast, thereby instigating changes in the concentrations of several lipid classes. We demonstrate that such changes in the cellular lipidome initiate three mechanisms of aging delay and longevity extension. The first mechanism through which PE21 slows aging and prolongs longevity consists in its ability to decrease the intracellular concentration of free fatty acids. This postpones an age-related onset of liponecrotic cell death promoted by excessive concentrations of free fatty acids. The second mechanism of aging delay and longevity extension by PE21 consists in its ability to decrease the concentrations of triacylglycerols and to increase the concentrations of glycerophospholipids within the endoplasmic reticulum membrane. This activates the unfolded protein response system in the endoplasmic reticulum, which then decelerates an age-related decline in protein and lipid homeostasis and slows down an aging-associated deterioration of cell resistance to stress. The third mechanisms underlying aging delay and longevity extension by PE21 consists in its ability to change lipid concentrations in the mitochondrial membranes. This alters certain catabolic and anabolic processes in mitochondria, thus amending the pattern of aging-associated changes in several key aspects of mitochondrial functionality.

## INTRODUCTION

The budding yeast *Saccharomyces cerevisiae* is amenable to thorough molecular analyses and has relatively short and easily measurable chronological and replicative lifespans [1–7]. The use of this unicellular eukaryote with a sequenced genome as a model organism in aging research has provided fundamental insights on mechanisms of cellular aging [1, 2, 5, 6]. Studies in *S. cerevisiae* uncovered genes, signaling pathways and chemical compounds that postpone cellular aging not only in unicellular eukaryotes but also in evolutionarily diverse metazoans [1, 2, 5, 6, 8–18]. After being discovered in yeast, these genes, signaling pathways and chemical compounds appeared to extend healthy lifespan also in multicellular eukaryotes across phyla. It is believed therefore that the key aspects of the aging process and mechanisms of its delay by certain genetic, dietary and pharmacological interventions have been conserved during evolution [1, 6, 8, 17, 18].

Aging of unicellular eukaryotes and metazoans is an intricate biological phenomenon of an age-related functional deterioration [19, 20]. Such aging-associated functional decline impairs the regulation of a distinct set of cellular processes, thus making an organism more susceptible to disease and death [19, 20]. Cellular processes whose progressive dysregulation has been implicated in cellular and organismal aging of eukaryotes across phyla include cell cycle regulation, quiescent state maintenance by adult stem cells, cell growth, stress response, cellular signaling, apoptosis and other modes of regulated cell death (RCD), autophagy (including mitophagy), actin organization, nuclear DNA replication, chromatin assembly and maintenance, ribosome biogenesis and protein synthesis in the cytosol and mitochondria, protein folding, proteasomal degradation of misfolded proteins, oxidative and biosynthetic metabolic pathways in mitochondria, lipid and carbohydrate metabolism, NAD^+^ homeostasis, amino acid biosynthesis and degradation, and ammonium and amino acid uptake [19–35]. All these processes are controlled by a nutrient-sensing signaling network of longevity regulation that in evolutionarily distant metazoans integrates the insulin/insulin-like growth factor 1 (IGF-1) pathway, the AMP-dependent protein kinase (AMPK) pathway, the mammalian target of rapamycin complex 1 (mTORC1) pathway, the sirtuin-governed protein deacetylation module and the cAMP/protein kinase A (cAMP/PKA) pathway [1, 19, 21, 23, 36–39].

In chronologically aging *S. cerevisiae*, the nutrient-sensing signaling network of longevity regulation incorporates the TORC1, cAMP/PKA, Pkb-activating kinase homolog (PKH1/2), sucrose non-fermenting (SNF1) and autophagy (ATG) pathways [1, 5, 11, 32, 40–51]. The network also integrates the serine/threonine-specific protein kinases Sch9 (which is stimulated by the TORC1 and PKH1/2 pathways) and Rim15 (which is inhibited by the TORC1, PKA and PKH1/2 pathways) [1, 5, 11, 32, 40–51]. Certain chemical compounds of bacterial, fungal, plant or mammalian origin can delay the chronological aging and extend longevity of *S. cerevisiae* because they regulate the flow of information along these convergent, divergent and multiply branched signaling pathways and protein kinases. Such aging-delaying chemicals include resveratrol, rapamycin, caffeine, spermidine, myriocin, methionine sulfoxide, lithocholic acid and cryptotanshinone [1, 2, 11, 13, 44, 48, 52–54].

In search for novel chemical compounds that can delay aging and prolong longevity of chronologically aging yeast, we have recently conducted a screen of many extracts from plants used in traditional Chinese herbal medicines or in the Mediterranean diet [55]. Our screen revealed several aging-delaying and longevity-extending plant extracts (PEs). One of them is PE21, an extract from the white willow *Salix alba* [55]. PE21 delays yeast chronological aging much more efficiently than any of the previously known pharmacological interventions [55]. We demonstrated that PE21 slows aging by inhibiting a form of the pro-aging protein kinase Sch9 that is stimulated by the pro-aging PKH1/2 signaling pathway [56]. Such PE21-dependent inhibition of Sch9 coincides with changes in several cellular processes known to regulate longevity of *S. cerevisiae* [55]. In this study, we investigated mechanisms through which PE21 delays chronological aging of *S. cerevisiae* and extends its longevity. We show that these mechanisms involve a specific remodeling of the cellular lipidome, a stimulation of the unfolded protein response in the endoplasmic reticulum (ER), and an activation of catabolic and anabolic processes in mitochondria.

## RESULTS

### An overview of longevity-defining and geroprotective cellular processes affected by PE21

Our previous study has revealed that PE21 extends longevity of chronologically aging yeast cultured in a synthetic minimal medium initially containing 2% (w/v) glucose [55]. Yeast cells cultured on 2% glucose are not limited in calorie supply or intake and, thus, undergo chronological aging under so-called non-caloric restriction (non-CR) conditions [1, 2, 6]. Non-CR conditions are known to speed up chronological aging in yeast [1, 2, 6]. We also reported that PE21 prolongs yeast chronological lifespan (CLS) of yeast cultured under CR conditions on 0.5% (w/v) glucose significantly less efficiently than it does under non-CR conditions [55]. CR conditions have been shown to slow down yeast chronological aging [1, 2, 6]. Because the longevity-extending efficiency of PE21 under non-CR conditions significantly exceeds that under CR conditions, we concluded that PE21 is a CR-mimetic [55]. CR mimetics are pharmacological interventions that under non-CR conditions target the same set of longevity-defining cellular processes as CR, thus delaying aging even if calorie supply and intake are not limited [57–59].

In yeast cultured under non-CR conditions on 2% glucose, PE21 is a geroprotector that elicits a hormetic stress response and imposes changes in certain cellular processes [55]. Specifically, PE21 alters the following aspects of mitochondrial functionality: 1) it significantly increases the rate of coupled mitochondrial respiration during post-diauxic (PD) growth phase (which occurs on day 2 of culturing) and stationary (ST) growth phase (which occurs after 2 days of culturing); 2) it prolongs mitochondrial functionality by preventing an age-related decline in mitochondrial membrane potential during PD and ST growth phases; and 3) it alters the pattern of age-related changes in intracellular reactive oxygen species (ROS) that are created mainly as by-products of mitochondrial respiration [60, 61]; such PE21-dependent pattern alterations consist in decreasing ROS concentration during logarithmic (L) growth phase on day 1 of culturing and during PD growth phase, and in lowering the extent to which ROS concentration declines during ST phase [55]. Furthermore, PE21 significantly decreases the extent of oxidative damage to cellular proteins and membrane lipids during ST phase [55]. Moreover, PE21 substantially lowers the frequencies of spontaneous point mutations in the DNA within the nucleus and mitochondria during ST phase, likely because PE21 decreases the extent of oxidative damage to nuclear and mitochondrial DNA [55]. PE21 also considerably increases cell resistance to chronic oxidative and thermal stresses during ST phase [55]. In addition, PE21 promotes a rapid age-related degradation of neutral lipids (i. e. triacylglycerols [TAG] and ergosterols) stored in lipid droplets (LD) during PD and ST phases [55].

### PE21 alters the relative levels of different lipid classes in an age-related manner

The maintenance of lipid homeostasis is indispensable for healthy aging in yeast and metazoans because lipid metabolism and transport are essential contributors to the aging process in unicellular and multicellular eukaryotes [32, 62–115]. Since PE21 promotes a rapid age-related degradation of neutral lipids deposited in LD [55], we sought to determine whether PE21 may affect the abundance of other lipid classes in chronologically aging yeast under non-CR conditions. We therefore used quantitative mass spectrometry to compare the cellular lipidome of wild-type (WT) yeast cultured under non-CR conditions on 2% glucose with 0.1% (w/v) PE21 to the cellular lipidome of WT cells cultured on 2% glucose without PE21. If PE21 is used at the final concentration of 0.1% with ethanol being utilized as a vehicle at the final concentration of 0.5% (v/v), this PE exhibits the highest efficacy of yeast CLS extension under non-CR conditions on 2% glucose; this is in comparison to WT cells subjected to ethanol-mock treatment by being cultured in growth medium initially containing 2% glucose and 0.5% ethanol [55]. Cells for lipid extraction and mass spectrometric lipidomics were recovered on days 1, 2, 3 and 4 of culturing on 2% glucose because only 11.7 ± 4.4% (*n =* 35) of WT cells cultured without PE21 were viable after 4 days of such culturing [55]. In contrast, 95.6 ± 3.1% (*n =* 35) of WT cells cultured with 0.1% PE21 were viable after 4 days of culturing on 2% glucose [55].

**Figure 1:**
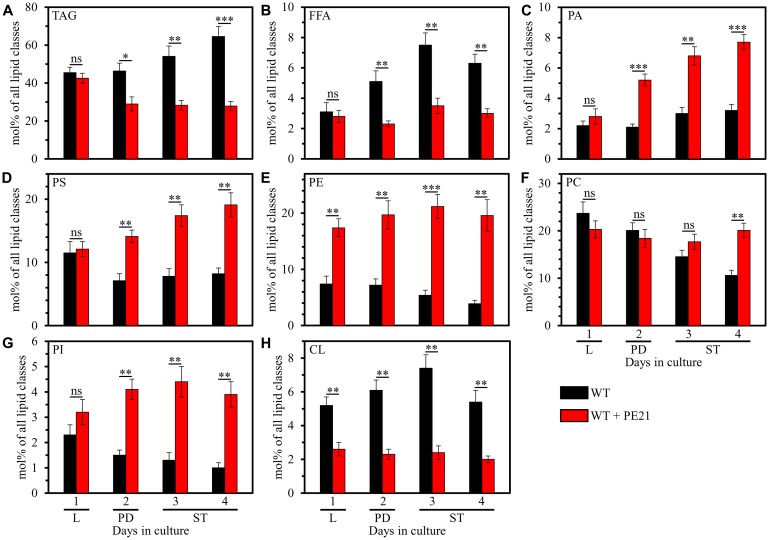
PE21 exhibits age-dependent differential effects on the relative levels of different lipid classes. Cells of the wild-type (WT) strain were grown in the synthetic minimal YNB medium (0.67% [w/v] yeast nitrogen base without amino acids) initially containing 2% (w/v) glucose, in the presence of 0.1% (w/v) PE21 (ethanol was used as a vehicle at the final concentration of 0.5% [v/v]) or in its absence (cells were subjected to ethanol-mock treatment). Cells were recovered on days 1, 2, 3 and 4 of culturing. Extraction of cellular lipids and mass spectrometric identification and quantitation of different lipid classes were carried out as described in Materials and Based on these data, the relative levels of triacylglycerols [TAG] (**A**), free fatty acids [FFA] (**B**), phosphatidic acid [PA] (**C**), phosphatidylserine [PS] (**D**), phosphatidylethanolamine [PE] (**E**), phosphatidylcholine [PC] (**F**), phosphatidylinositol [PI] (**G**) and cardiolipin [CL] (**H**) were calculated as mol% of all lipid classes in cells recovered on day 1, 2, 3 or 4 of culturing. Data are presented as means ± SEM (*n = *4; ^*^ *p < *0.05; ^**^ *p < *0.01; ^***^ *p < *0.001; ns, not significant). Abbreviations: Logarithmic (L), post-diauxic (PD) or stationary (ST) growth phase.

PE21 exhibited differential effects on the relative levels of different lipid classes calculated as mol% of all lipids; moreover, these effects of PE21 were age-related. Indeed, we found that 1) PE21 elicits a significant decline in the relative levels of TAG, free (i. e. unesterified) fatty acids (FFA) and the signature mitochondrial membrane lipid cardiolipin (CL); 2) the extent to which PE21 lowers the relative levels of TAG, FFA and CL is gradually increased with the chronological age of WT cells; 3) PE21 causes a significant decline in the relative level of CL in WT cells recovered at L phase (on day 1 of culturing), PD phase (on day 2 of culturing) and ST phase (on days 3 and 4 of culturing); and 4) PE21 significantly decreases the relative levels of TAG and FFA only in WT cells recovered at PD or ST phase of culturing (Figure 1A, 1B and 1H). Our mass spectrometric identification and quantitation of cellular lipids also revealed that 1) PE21 causes a significant rise in the relative levels of all membrane glycerophospholipids, including phosphatidic acid (PA), phosphatidylserine (PS), phosphatidylethanolamine (PE), phosphatidylcholine (PC) and phosphatidylinositol (PI); 2) the extent of such effect of PE21 on the relative levels of PA, PS, PE, PC and PI is gradually increased with the chronological age of WT cells; 3) PE21 elicits a significant rise in the relative level of PE in WT cells recovered at L phase (on day 1 of culturing), PD phase (on day 2 of culturing) and ST phase (on days 3 and 4 of culturing); 4) PE21 significantly raises the relative levels of PA, PS and PI only in WT cells recovered at PD or ST phase of culturing; and 5) PE21 causes a significant increase in the relative level of PC only in WT cells recovered at ST phase on day 4 of culturing (Figure 1C–1G).

In sum, these findings indicate that PE21 causes significant age-related changes in the relative levels of different lipid classes in WT cells under non-CR conditions.

### PE21 causes a specific remodeling of lipid metabolism and transport in chronologically aging yeast, likely by redirecting the flows of FFA and PA into different classes of lipids

Our findings that PE21 alters the relative levels of FFA, the neutral lipid TAG, the signature mitochondrial membrane lipid CL and all classes of membrane glycerophospholipids suggest that PE21 may instigate a specific remodeling of lipid metabolism and transport in several organelles of chronologically aging yeast. The metabolic and interorganellar transport processes that define the concentrations of all these lipid classes in yeast cells are well known [32, 116–149]. These processes are catalyzed by enzymes that reside in the cytosol, ER, mitochondria, LD and peroxisomes (Supplementary Figure 1) [32, 116–149].

Glucose, the only carbon source exogenously added to yeast cultures in this study, is initially converted to pyruvate via the glycolytic pathway in the cytosol (Supplementary Figure 1). The glycolytically produced pyruvate is then used for the synthesis of acetyl-CoA (Ac-CoA) through three consecutive reactions catalyzed by the cytosolic pyruvate decarboxylase isozymes Pdc1, Pdc5 and Pdc6, aldehyde dehydrogenases Ald2-Ald6, and Ac-CoA synthetase isoforms Acs1 and Acs2 (Supplementary Figure 1). After being synthesized in the cytosol, Ac-CoA is used as a substrate for the formation of FFA by the cytosolic Ac-CoA carboxylase Acc1 and FA synthase complex Fas1/Fas2 (Supplementary Figure 1). The cytosolic pool of Ac-CoA used for the formation of FFA by Acc1 and Fas1/Fas2 is also created as the product of peroxisomal β-oxidation of FFA in Fox1-, Fox2- and Fox3-dependent chemical reactions (Supplementary Figure 1). Other sources of FFA are the hydrolysis of TAG by the lipases Tgl1, Tgl3, Tgl4 and Tgl5 confined to LD (Supplementary Figure 1), as well as the lipolytic degradation of TAG-derived diacylglycerols (DAG) and monoacylglycerols (MAG) by the lipases Tgl3 and Yju3 (respectively) in LD (Supplementary Figure 1).

After FFA are formed from Ac-CoA, TAG, DAG or MAG, they are activated to yield fatty acyl-CoA esters (FA-CoA) in reactions catalyzed by the long chain acyl-CoA synthetases Faa1, Faa4 and Fat1 in the ER (Supplementary Figure 1). These FA-CoA are then used for the *de novo* synthesis of TAG, glycerophospholipids and CL by enzymes confined to the ER and mitochondria (Supplementary Figure 1). This *de novo* synthesis begins in the ER where the glycerol-3-phosphate/dihydroxyacetone phosphate acyltransferases Sct1 and Gpt2 catalyze the formation of lysophosphatidic acid (LPA) or acyl-dihydroxyacetone phosphate (ADHAP) from FA-CoA and glycerol-3-phosphate or DHAP, respectively (Supplementary Figure 1). An Ayr1-driven reaction converts ADHAP to LPA (Supplementary Figure 1). The LPA formed in an Sct1-, Gpt2- and Ayr1-dependent manner is then converted to PA in an acyl CoA-dependent reaction catalyzed by the LPA acyl-transferases Slc1, Slc4, Loa1 and Ale1 (Supplementary Figure 1). A Cds1-driven reaction converts PA to cytidine diphosphate (CDP)-DAG, which is then used as a common precursor for the Cho1-dependent synthesis of PS in the ER, transfer of PS from the ER to the outer mitochondrial membrane (OMM) via mitochondria-ER contact sites, Ups2-driven transport of PS from the OMM to the inner mitochondrial membrane (IMM) via the intermediate space (IMS), Psd1-dependent synthesis of PE in the IMM, transfer of PE from the IMM across the IMS to the OMM and then to the ER via mitochondria-ER contact sites, Pis1-dependent synthesis of PI in the ER, and Cho2- and Opi3-dependent synthesis of PC in the ER (Supplementary Figure 1). PA can also be converted to DAG in a reaction catalyzed by the PA phosphatases Pah1, App1, Dpp1 and Lpp1 in the ER (Supplementary Figure 1). The ensuing acylation of DAG to TAG occurs in an FA-CoA-dependent reaction driven by Dga1, Are1 and Are2, and in a PE- and PC-dependent reaction catalyzed by Lro1 (Supplementary Figure 1). After the *de novo* synthesis of TAG in the ER, TAG are deposited in LD (Supplementary Figure 1). In addition, PA can move from the ER to the OMM via mitochondria-ER contact sites and then from the OMM to the IMM in an Ups1-dependent transfer reaction inhibited by CL (Supplementary Figure 1). After the ER-derived PA is delivered to the IMM, it is converted into CDP-DAG, phosphatidylglycerol (PG), CL and monolysocardiolipin (MLCL) in reactions catalyzed by Tam41, Pgs1, Gep4, Crd1, Cld1 and Taz1 (Supplementary Figure 1).

Considering the intensive knowledge of lipid metabolism and interorganellar transport in yeast cells, our data on PE21-dependent changes in the cellular lipidome indicate that PE21 redirects the flows of FFA and PA into different classes of lipids to cause a specific reorganization of lipid metabolism and transport in chronologically aging yeast. A model of such PE21-driven reorganization of lipid metabolism and transport in yeast cells is schematically depicted in Supplementary Figure 2. In this model, PE21 alters the efficiencies with which FFA and PA are incorporated into the synthesis of other lipids as follows: 1) it intensifies FFA incorporation into PA, thus lowering FFA concentration and increasing PA concentration; 2) it decreases the efficiency of PA flow into the synthesis of TAG in the ER, thereby lowering TAG concentration, decreasing the concentration of FFA derived from TAG lipolysis and rising PA concentration; 3) it intensifies PA entry into the synthesis of glycerophospholipids in the ER and mitochondria, thus increasing the concentrations of PS, PE, PC and PI in the ER and rising PS and PE concentrations in mitochondria; and 4) it lowers the efficiency of PA transport from the ER to the OMM and then to the IMM, thereby decreasing the concentrations of PA-derived CL in mitochondrial membranes (Supplementary Figure 2).

### Our hypothesis on three possible mechanisms through which PE21 may delay yeast chronological aging and extend yeast longevity

**Figure 2:**
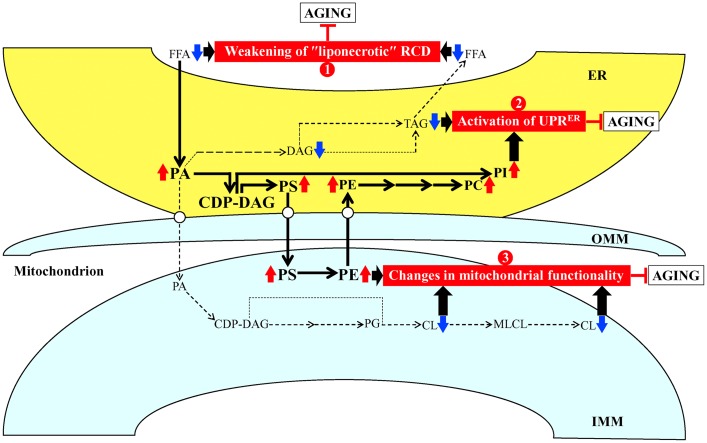
Possible mechanisms through which PE21 may delay yeast chronological aging. Arrows next to the names of lipid classes denote those of them whose concentrations are increased (red arrows) or decreased (blue arrows) in yeast cells cultured in the presence of PE21. The thickness of black arrows is proportional to the efficiency with which free fatty acids (FFA) and phosphatidic acid (PA) are included into the synthesis of other lipid classes. There may be at least three different mechanisms by which PE21 delays yeast chronological aging. These mechanisms are numbered. Mechanism 1: PE21 maintains FFA concentration below a toxic threshold, thus weakening an age-related form of FFA-driven liponecrotic regulated cell death (RCD). Mechanism 2: PE21 suppresses TAG formation and promotes glycerophospholipid synthesis in the endoplasmic reticulum (ER), thereby activating the unfolded protein response in the ER (UPR^ER^). Mechanism 3: PE21 increases phosphatidylserine (PS) and phosphatidylethanolamine (PE) concentrations and lower cardiolipin (CL) concentration in mitochondria, thus altering mitochondrial functionality. See text for more details. Other abbreviations: CDP, cytidine diphosphate; DAG, diacylglycerol; IMM, inner mitochondrial membrane; MLCL, monolysocardiolipin; OMM, outer mitochondrial membrane; PC, phosphatidylcholine; PG, phosphatidylglycerol; PI, phosphatidylinositol; TAG, triacylglycerol.

Based on the abilities of PE21 to cause a specific remodeling of lipid metabolism and transport (the present study) and to impose changes in certain cellular processes [55] within yeast cultured under non-CR conditions, we put forward a hypothesis that there may be at least three different mechanisms by which PE21 delays yeast chronological aging and extends yeast CLS. These possible mechanisms are outlined below and schematically depicted in Figure 2.

First mechanism: the present study demonstrates that PE21 lowers FFA concentration, likely because it intensifies FFA incorporation into PA (Figure 1B and 1C; Supplementary Figure 2). The present study also reveals that PE21 decreases the concentration of TAG (Figure 1A; Supplementary Figure 2), the major form of FFA storage [22, 32, 120, 121, 126, 129, 135, 136, 146]; this may further contribute to the PE21-driven decline in FFA concentration because the lipolysis of TAG in chronologically aging yeast is known to be a source of the bulk quantities of FFA [22, 32, 120, 121, 126, 129, 135, 136, 146]. An exposure of yeast cells to exogenous FFA has been shown to promote a “liponecrotic” form of RCD in an age-related manner [32, 52, 137, 150–155]. Such exposure elicits the incorporation of FFA into membrane glycerophospholipids and TAG, thereby reorganizing lipid metabolism and transfer in the ER, mitochondria, LD and the plasma membrane (PM) [32, 52, 137, 150–155]. Certain aspects of the FFA-driven reorganization of lipid metabolism and transfer are essential contributors to the commitment of yeast to liponecrosis or to the execution of this mode of RCD [32, 52, 137, 150–155]. These aspects include the following: 1) an excessive rise in PM permeability for small molecules; 2) a decline in mitochondrial functionality; 3) an excessive production of ROS in mitochondria; 4) an oxidative damage to various cellular organelles, which promotes massive autophagic degradation of these organelles; and 5) an oxidative impairment of the bulk quantities of cellular proteins, which disturbs cellular proteostasis by eliciting a build-up of dysfunctional, unfolded and aggregated proteins in the cytosol [32, 52, 137, 150–155]. Because the accumulation of excessive quantities of FFA actively increases the risk of liponecrotic cell death and decreases the chance of cell survival throughout chronological lifespan, FFA accumulation in quantities exceeding a toxic threshold shortens longevity of chronologically aging yeast [32, 52, 137, 150–155]. It needs to be emphasized that PE21 not only extends yeast longevity but also affects those aspects of the FFA-driven reorganization of the cellular lipidome that contribute to the commitment or execution of liponecrotic RCD [55]. Indeed, PE21 slows an age-related decline in mitochondrial functionality, alters the pattern of age-related changes in mitochondrially produced ROS and decreases the extent of oxidative damage to cellular proteins [55]. Taken together, these findings suggest that the first mechanism through which PE21 may delay yeast chronological aging and extend yeast CLS consists in the ability of PE21 to lower FFA concentration, thus maintaining FFA concentration below the toxic threshold and weakening an age-related form of FFA-driven liponecrotic RCD (Figure 2).

Second mechanism: the present study shows that PE21 causes significant perturbations in the relative levels of membrane lipids within the ER by weakening TAG formation and strengthening glycerophospholipid synthesis in this organelle (Figure 1B–1G; Supplementary Figure 2). Such perturbations in the relative levels of ER membrane lipids are known to stimulate the unfolded protein response in the ER (UPR^ER^) in yeast and metazoans, either by weakening the folding of ER proteins and eliciting their accumulation in the ER or without causing unfolded protein stress within this organelle [156–179]. When activated, the UPR^ER^ system allows to reinstate protein and lipid homeostasis in the ER. Such reinstatement is achieved because the activated UPR^ER^ system slows protein synthesis in the ER, stimulates *N*-linked protein glycosylation of ER proteins, promotes a refolding of improperly folded ER proteins, directs other improperly folded proteins accumulated in the ER for the removal by ER-associated degradation or autophagy, enhances vesicular traffic from the ER throughout the secretory pathway, and activates the synthesis of membrane lipids in the ER [176, 180–186]. A body of evidence indicates that the UPR^ER^ system of protein and lipid homeostasis restoration within the ER is indispensable for preventing an age-related decline in protein and lipid homeostasis maintenance within the entire cell; this is because the UPR^ER^ system slows protein synthesis, weakens oxidative and thermal protein damage, promotes protein folding and vesicular transport, stimulates autophagic and proteasomal degradation of improperly folded proteins, and controls lipid metabolism within the entire cell [161, 167, 170, 174–176, 178, 181, 185–202]. As such, the UPR^ER^ system is commonly perceived as a process that is essential for delaying cellular and organismal aging and slowing down the onset of aging-associated disorders [160, 163, 167, 181, 185–202]. Of note, the ability of PE21 to extend longevity of chronologically aging yeast coincides with its abilities to decrease the extent of oxidative damage to cellular proteins, lipids and nucleic acids, and to increase cell resistance to chronic oxidative stress [55]. In sum, the above findings suggest that the second mechanism by which PE21 may delay yeast chronological aging and extend yeast CLS consists in its ability to alter the ER lipidome, thus activating UPR^ER^ (Figure 2). Our hypothesis posits that such PE21-driven activation of the UPR^ER^ system may be responsible for the observed abilities of PE21 to slow down an age-related decline in protein, lipid and nucleic acid homeostasis and to decelerate an aging-associated weakening of cell resistance to oxidative and thermal stresses [55].

Third mechanism: the present study reveals that PE21 alters the membrane lipidome of mitochondria by rising PS and PE concentrations and lowering CL concentration in these organelles (Figure 1D, 1E and 1H; Supplementary Figure 2). A body of evidence supports the notion that the composition of mitochondrial membrane lipids is an essential contributor to mitochondrial functionality and as such, the mitochondrial membrane lipidome defines longevity of yeast and multicellular eukaryotes [32, 34, 77, 87, 97, 103, 104, 108, 203–209]. Notable, PE21 not only prolongs yeast longevity but also amends the pattern of age-related changes in several key aspects of mitochondrial functionality, including mitochondrial respiration, mitochondrial membrane potential and mitochondrial ROS production [55]. These findings suggest that the third mechanism by which PE21 may delay yeast chronological aging and extend yeast longevity consists in its ability to reorganize processes confined to mitochondria, thus altering mitochondrial functionality (Figure 2).

In a series of experiments outlined below, we assessed how each of the three mechanisms contributes to the extension of yeast CLS by PE21.

### PE21 extends longevity of chronologically aging yeast in part because it delays the age-related onset of FFA-dependent liponecrotic RCD

**Figure 3:**
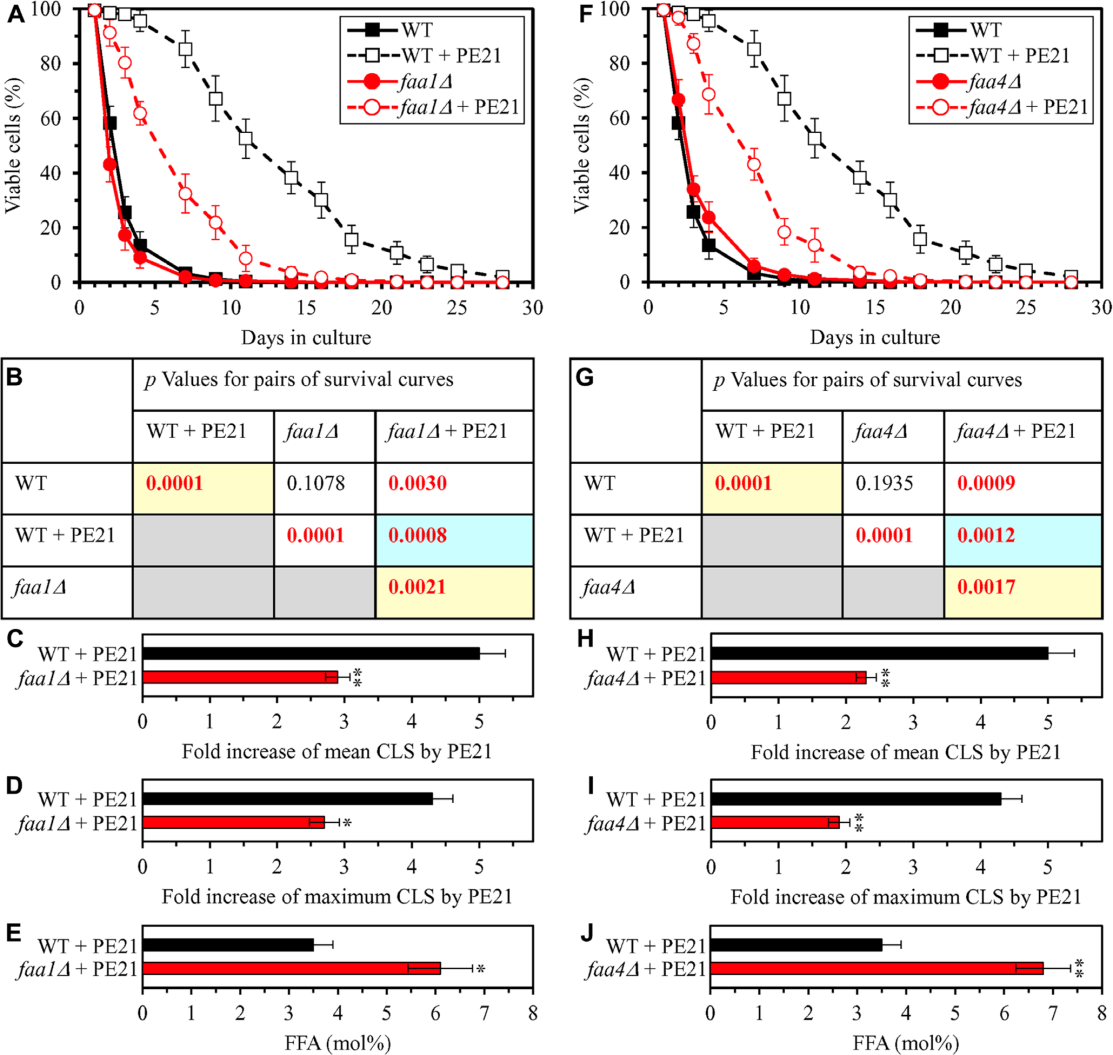
The *faa1Δ* and *faa4Δ* mutations eliminate enzymes involved in the incorporation of FFA into PA. These mutations increase cellular FFA concentration and decrease the efficiency with which PE21 prolongs yeast chronological lifespan (CLS). WT cells and mutant cells carrying a single-gene-deletion mutation eliminating either Faa1 or Faa4 were cultured in the synthetic minimal YNB medium initially containing 2% glucose with 0.1% PE21 or without it. (**A**, **F**) Survival curves of the chronologically aging WT and *faa1Δ* (**A**) or WT and *faa4Δ* (**F**) strains are shown. Data are presented as means ± SEM (*n = *3). Data for the WT strain cultured with or without PE21 are replicated in the graphs of (**A**) and (**F**) and Figures 4A, 4F, 5A, 5F, 6A, 6F, 10A–10D, 11A–11C, 14A–14D, 15A–15D. (**B**, **G**) *p* Values for different pairs of survival curves of the WT and *faa1Δ* (**B**) or WT and *faa4Δ* (**G**) strains cultured with or without PE21. Survival curves shown in A or F (respectively) were compared. Two survival curves were considered statistically different if the *p* value was less than 0.05. The *p* values for comparing pairs of survival curves using the logrank test were calculated as described in Materials and Methods. The *p* values displayed on a yellow color background indicate that PE21 statistically significantly prolongs the CLS of the WT, *faa1Δ* (**B**) and *faa4Δ* (**G**) strains. The *p* values displayed on a blue color background indicate that PE21 prolongs the CLS of the *faa1Δ* (**B**) and *faa4Δ* (**G**) strains to a lower extent than that of the WT strain. (**C**, **D**, **H**, **I**) Survival curves shown in (**A**, **F**) were used to calculate the fold of increase of the mean (**C**, **H**) and maximum (**D**, **I**) CLS by PE21 for the WT and *faa1Δ* (**C**, **D**) and WT and *faa4Δ* (**H**, **I**) strains. Data are presented as means ± SEM (*n = *3; ^*^ *p < *0.05; ^**^ *p < *0.01). (**E**, **J**) The maximum concentration of free fatty acids (FFA), which was observed in WT and *faa1Δ* (**E**) or WT and *faa4Δ* (**J**) cells recovered on day 3 of culturing with PE21, is shown. Data are presented as means ± SEM (*n = *4; ^*^ *p < *0.05; ^**^ *p < *0.01).

**Figure 4:**
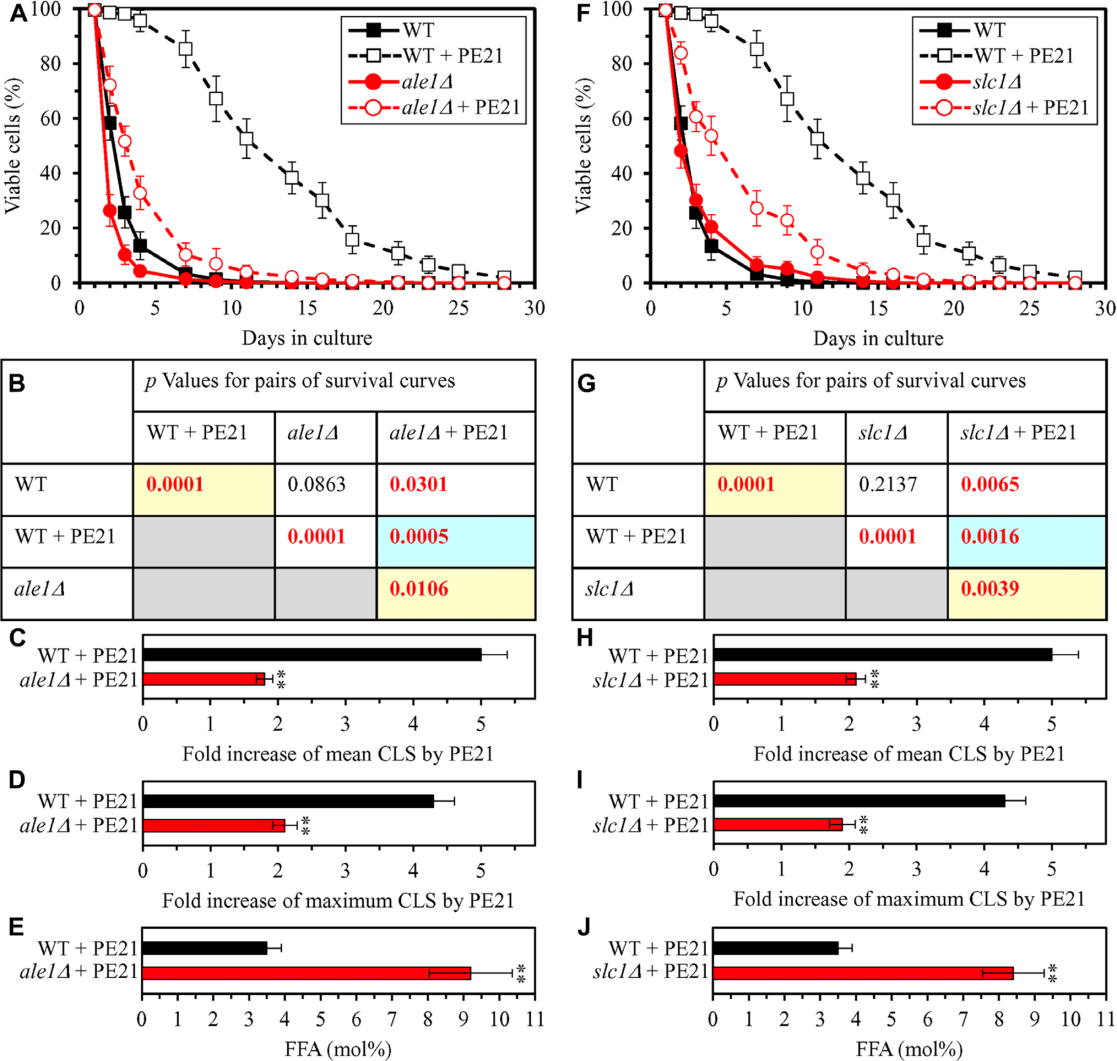
The *ale1Δ* and *slc1Δ* mutations eliminate enzymes involved in the incorporation of FFA into PA. These mutations increase cellular FFA concentration and decrease the efficiency with which PE21 prolongs yeast CLS. WT cells and mutant cells carrying a single-gene-deletion mutation eliminating either Ale1 or Slc1 were cultured in the synthetic minimal YNB medium initially containing 2% glucose with 0.1% PE21 or without it. (**A**, **F**) Survival curves of the chronologically aging WT and *ale1Δ* (**A**) or WT and *slc1Δ* (**F**) strains are shown. Data are presented as means ± SEM (*n = *3). Data for the WT strain cultured with or without PE21 are replicated in the graphs of (**A**) and (**F**) and Figures 3A, 3F, 5A, 5F, 6A, 6F, 10A–10D, 11A–11C, 14A–14D, 15A–15D. (**B**, **G**) *p* Values for different pairs of survival curves of the WT and *ale1Δ* (**B**) or WT and *slc1Δ* (**G**) strains cultured with or without PE21. Survival curves shown in (**A)** or (**F)** (respectively) were compared. Two survival curves were considered statistically different if the *p* value was less than 0.05. The *p* values for comparing pairs of survival curves using the logrank test were calculated as described in Materials and Methods. The *p* values displayed on a yellow color background indicate that PE21 statistically significantly prolongs the CLS of the WT, *ale1Δ* (**B**) and *slc1Δ* (**G**) strains. The *p* values displayed on a blue color background indicate that PE21 prolongs the CLS of the *ale1Δ* (**B**) and *slc1Δ* (**G**) strains to a lower extent than that of the WT strain. (**C**, **D**, **H**, **I**) Survival curves shown in (**A**, **F**) were used to calculate the fold of increase of the mean (**C**, **H**) and maximum (**D**, **I**) CLS by PE21 for the WT and *ale1Δ* (**C**, **D**) and WT and *slc1Δ* (**H**, **I**) strains. Data are presented as means ± SEM (*n = *3; ^**^ *p < *0.01). (**E**, **J**) The maximum concentration of free fatty acids (FFA), which was observed in WT and *ale1Δ* (**E**) or WT and *slc1Δ* (**J**) cells recovered on day 3 of culturing with PE21, is shown. Data are presented as means ± SEM (*n = *4; ^*^ *p < *0.05; ^**^ *p < *0.01).

Our hypothesis on the first mechanism through which PE21 may extend longevity of chronologically aging yeast predicts that mutations capable of increasing cellular FFA concentration will weaken the longevity-extending efficiency of PE21 (Figure 2). To test this prediction, we examined how a single-gene-deletion mutation eliminating the Faa1, Faa4, Ale1 or Slc1 protein affects the efficiency of yeast CLS extension by PE21 and how it influences the cellular concentration of FFA. Faa1, Faa4, Ale1 and Slc1 catalyze reactions of the incorporation of FFA into PA within the ER (Supplementary Figure 1) [32, 122, 125, 133]. A single-gene-deletion mutation eliminating either of these four proteins is known to increase the concentration of FFA in yeast cultured in the nutrient-rich YP (1% yeast extract and 2% peptone) medium initially containing 2% glucose [210–214]. It was unknown however if any of these mutations has a similar effect on FFA concentration in yeast cultured in a synthetic minimal YNB medium (0.67% Yeast Nitrogen Base) initially containing 2% glucose, i. e. under culturing conditions used in the present study. We found that the *faa1Δ*, *faa4Δ*, *ale1Δ* and *slc1Δ* mutations cause a significant decline in the efficiency with which PE21 can prolong both the mean and maximum CLS of *S. cerevisiae*, although the extent of such decline was different for each of these single-gene-deletion mutations (Figure 3A–3D and 3F–3I for *faa1Δ* and *faa4Δ*, respectively; Figure 4A–4D and 4F–4I for *ale11Δ* and *slc1Δ*, respectively). We also revealed that all these single-gene-deletion mutations substantially increase cellular FFA concentration; however, the extent of such rise in cellular FFA concentration was different for each of them (Figure 3E and 3J for *faa1Δ* and *faa4Δ*, respectively; Figure 4E and 4J for *ale11Δ* and *slc1Δ*, respectively). In sum, the above findings support our prediction that mutations increasing cellular FFA concentration can weaken the efficiency with which PE21 prolongs longevity of chronologically aging yeast.

**Figure 5:**
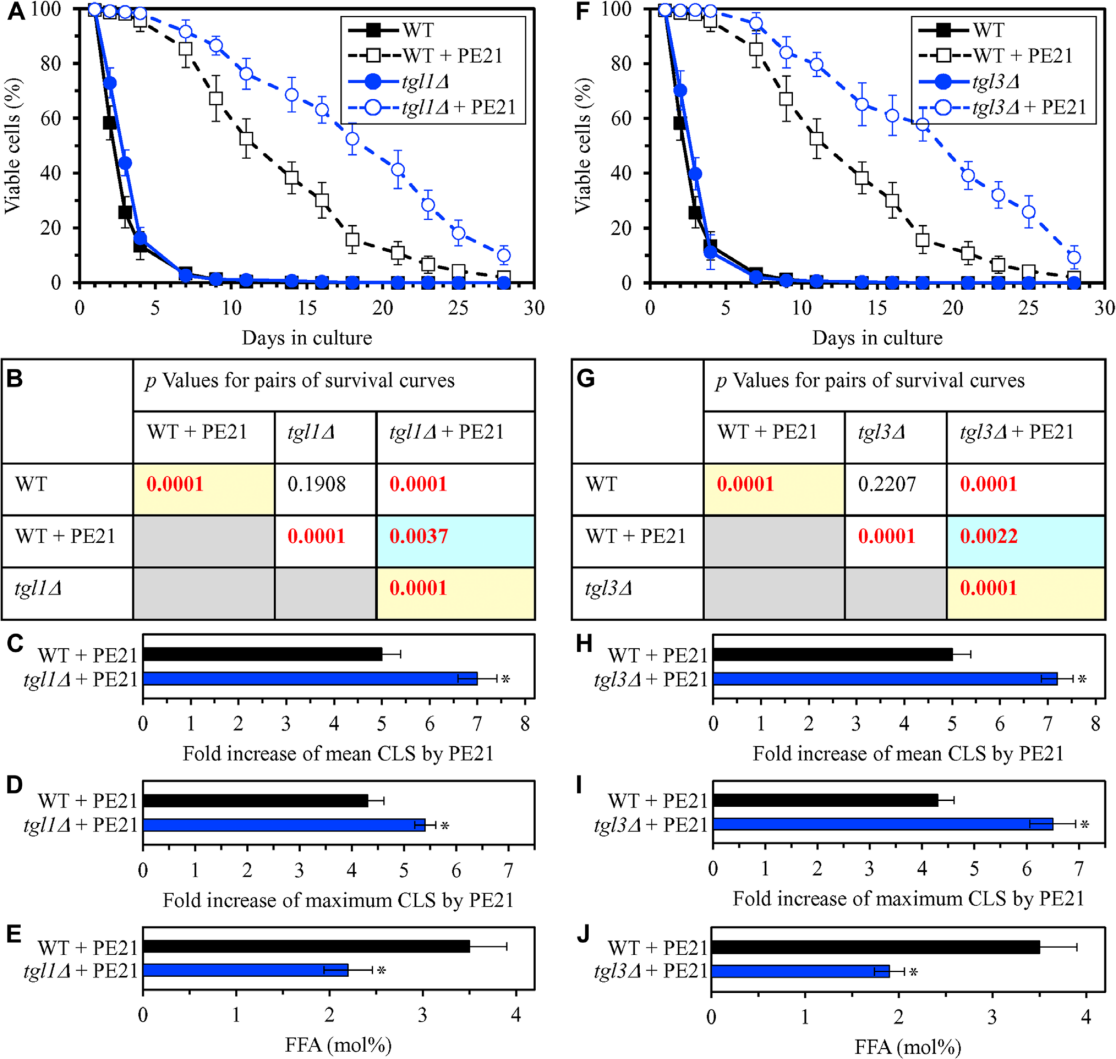
The *tgl1Δ* and *tgl3Δ* mutations eliminate enzymes involved in the formation of FFA as products of TAG lipolysis. These mutations decrease cellular FFA concentration and increase the efficiency with which PE21 prolongs yeast CLS. WT cells and mutant cells carrying a single-gene-deletion mutation eliminating either Tgl1 or Tgl3 were cultured in the synthetic minimal YNB medium initially containing 2% glucose with 0.1% PE21 or without it. (**A**, **F**) Survival curves of the chronologically aging WT and *tgl1Δ* (**A**) or WT and *tgl3Δ* (**F**) strains are shown. Data are presented as means ± SEM (*n = *3). Data for the WT strain cultured with or without PE21 are replicated in the graphs of (**A**) and (**F**) and Figures 3A, 3F, 4A, 4F, 6A, 6F, 10A–10D, 11A–11C, 14A–14D, 15A–15D. (**B**, **G**) *p* Values for different pairs of survival curves of the WT and *tgl1Δ* (**B**) or WT and *tgl3Δ* (**G**) strains cultured with or without PE21. Survival curves shown in (**A)** or (**F)** (respectively) were compared. Two survival curves were considered statistically different if the *p* value was less than 0.05. The *p* values for comparing pairs of survival curves using the logrank test were calculated as described in Materials and Methods. The *p* values displayed on a yellow color background indicate that PE21 statistically significantly prolongs the CLS of the WT, *tgl1Δ* (**B**) and *tgl3Δ* (**G**) strains. The *p* values displayed on a blue color background indicate that PE21 prolongs the CLS of the *tgl1Δ* (**B**) and *tgl3Δ* (**G**) strains to a lower extent than that of the WT strain. (**C**, **D**, **H**, **I**) Survival curves shown in (**A**, **F**) were used to calculate the fold of increase of the mean (**C**, **H**) and maximum (**D**, **I**) CLS by PE21 for the WT and *tgl1Δ* (**C**, **D**) and WT and *tgl3Δ* (**H**, **I**) strains. Data are presented as means ± SEM (*n = *3; ^*^ *p < *0.05). (**E**, **J**) The maximum concentration of free fatty acids (FFA), which was observed in WT and *tgl1Δ* (**E**) or WT and *tgl3Δ* (**J**) cells recovered on day 3 of culturing with PE21, is shown. Data are presented as means ± SEM (*n = *4; ^*^ *p < *0.05; ^**^ *p < *0.01).

**Figure 6:**
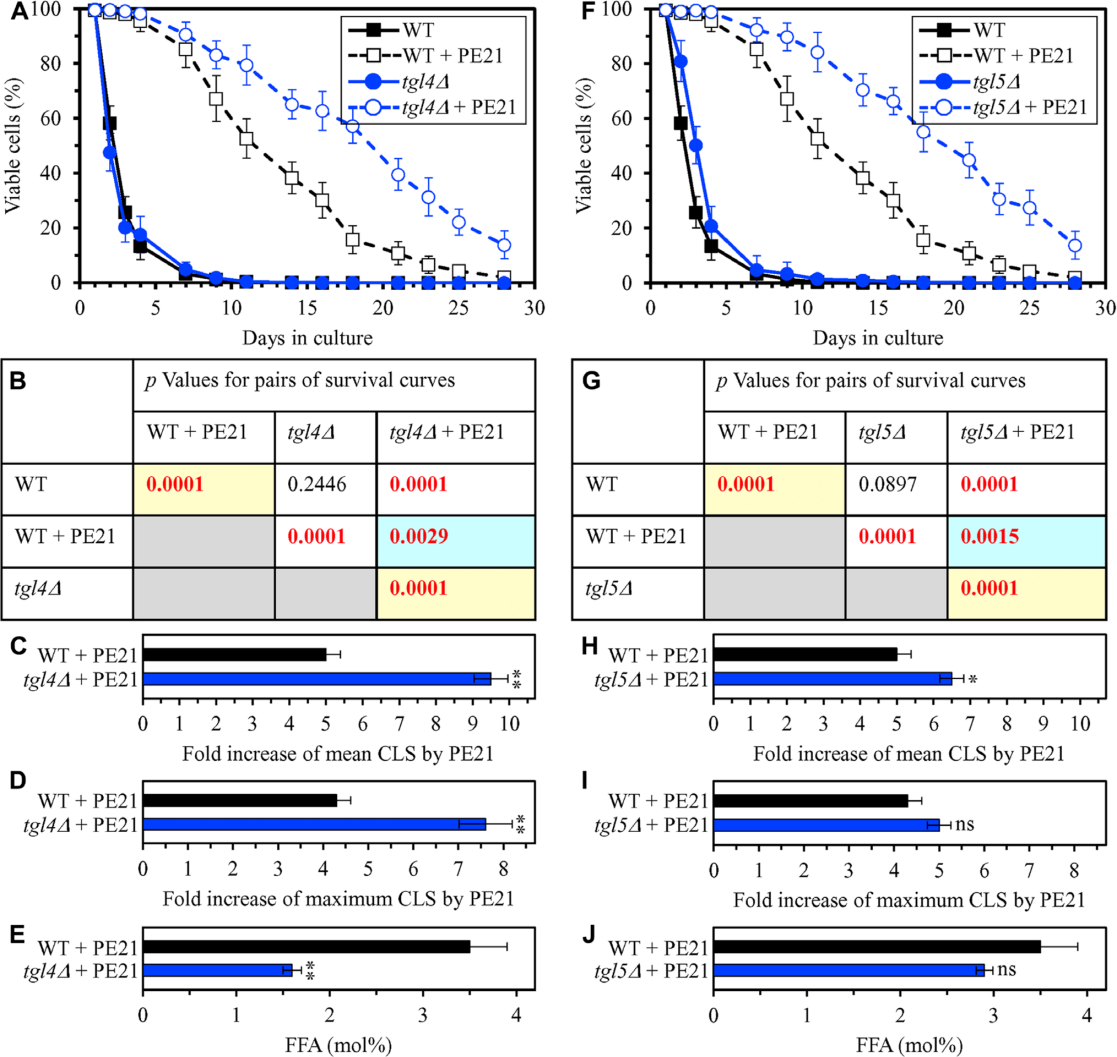
The *tgl4Δ* and *tgl5Δ* mutations eliminate enzymes that catalyze the formation of FFA as products of TAG lipolysis. These mutations cause a decline in cellular FFA concentration and elicit a rise in the efficiency of yeast CLS extension by PE21. WT cells and mutant cells carrying a single-gene-deletion mutation eliminating either Tgl4 or Tgl5 were cultured in the synthetic minimal YNB medium initially containing 2% glucose with 0.1% PE21 or without it. (**A**, **F**) Survival curves of the chronologically aging WT and *tgl4Δ* (**A**) or WT and *tgl5Δ* (**F**) strains are shown. Data are presented as means ± SEM (*n = *3). Data for the WT strain cultured with or without PE21 are replicated the graphs of (**A**) and (**F**) and Figures 3A, 3F, 4A, 4F, 5A, 5F, 10A–10D, 11A–11C, 14A–14D, 15A–15D. (**B**, **G**) *p* Values for different pairs of survival curves of the WT and *tgl4Δ* (**B**) or WT and *tgl5Δ* (**G**) strains cultured with or without PE21. Survival curves shown in (**A** or **F**) (respectively) were compared. Two survival curves were considered statistically different if the *p* value was less than 0.05. The *p* values for comparing pairs of survival curves using the logrank test were calculated as described in Materials and Methods. The *p* values displayed on a yellow color background indicate that PE21 statistically significantly extends the CLS of the WT, *tgl4Δ* (**B**) and *tgl5Δ* (**G**) strains. The *p* values displayed on a blue color background indicate that PE21 extends the CLS of the *tgl4Δ* (**B**) and *tgl5Δ* (**G**) strains to a lower extent than that of the WT strain. (**C**, **D**, **H**, **I**) Survival curves shown in (**A**, **F**) were used to calculate the fold of increase of the mean (**C**, **H**) and maximum (**D**, **I**) CLS by PE21 for the WT and *tgl4Δ* (**C**, **D**) and WT and *tgl5Δ* (**H**, **I**) strains. Data are presented as means ± SEM (*n = *3; ^**^ *p < *0.01; ns, not significant). (**E**, **J**) The maximum concentration of free fatty acids (FFA), which was observed in WT and *tgl4Δ* (**E**) or WT and *tgl5Δ* (**J**) cells recovered on day 3 of culturing with PE21, is shown. Data are presented as means ± SEM (*n = *4; ^*^ *p < *0.05; ^**^ *p < *0.01).

In our hypothesis on the first mechanism, mutations that decrease cellular FFA concentration are expected to enhance the longevity-extending efficiency of PE21 (Figure 2). We therefore investigated how a single-gene-deletion mutation eliminating the Tgl1, Tgl3, Tgl4 or Tgl5 protein influences the efficiency of yeast CLS extension by PE21 and how it affects the cellular concentration of FFA. Tgl1, Tgl3, Tgl4 and Tgl5 catalyze reactions of the formation of FFA as products of TAG lipolysis in LD (Supplementary Figure 1) [22, 32, 116, 118, 120, 121, 134–136]. A knock-out mutation removing either of these four proteins has been shown to increase the concentration of FFA in yeast cultured in the nutrient-rich YP medium initially supplemented with 2% glucose [215–217]. However, it remained unknown if any of these mutations has a similar effect on FFA concentration in yeast cultured in a synthetic minimal YNB medium initially supplemented with 2% glucose, i. e. under conditions yeast were cultured in the present study. We found that the *tgl1Δ*, *tgl3Δ*, *tgl4Δ* and *tgl5Δ* mutations increase the efficiency with which PE21 can extend yeast CLS and that the extent of such increase is different for each of these mutations (Figure 5A–5D and 5F–5I for *tgl1Δ* and *tgl3Δ*, respectively; Figure 6A–6D and 6F–6I for *tgl4Δ* and *tgl5Δ*, respectively). We also noted that all these mutations decrease cellular FFA concentration and that the extent of such decrease is different for each of them (Figure 5E and 5J for *tgl1Δ* and *tgl3Δ*, respectively; Figure 6E and 6J for *tgl3Δ* and *tgl4Δ*, respectively). Together, these data confirm that mutations decreasing cellular FFA concentration can enhance the longevity-extending efficiency of PE21.

Using the above data on the values of CLS and cellular FFA concentration for WT and mutant strains, we compared the PE21-dependent fold increase of mean or maximum CLS and the highest intracellular concentration of FFA in yeast cells cultured with PE21; FFA concentration was the highest in WT, *faa1Δ*, *faa4Δ*, *ale1Δ*, *slc1Δ*, *tgl1Δ*, *tgl3Δ*, *tgl4Δ* and *tgl5Δ* cells recovered on day 3 of culturing. We revealed that the Pearson’s correlation coefficient (*r*) values for the correlation between these two compared variables are less than - 0.9 for both possible pairwise combinations of the mean or maximum CLS and the highest intracellular concentration of FFA (Supplementary Figure 3). Because the Pearson’s *r* value ranging from -0.9 to -1.0 is considered a very high negative correlation between the two variables [218], we concluded that the PE21-dependent fold increase of mean or maximum CLS has a very high negative correlation with FFA concentration in the yeast cell. This observation confirms that, as predicted by our hypothesis on the first mechanism of PE21-dependent longevity extension, the efficiency of such extension inversely correlates with the intracellular concentration of FFA. Thus, PE21 delays yeast chronological aging and prolongs yeast CLS in part because it decreases FFA concentration in the yeast cell.

**Figure 7:**
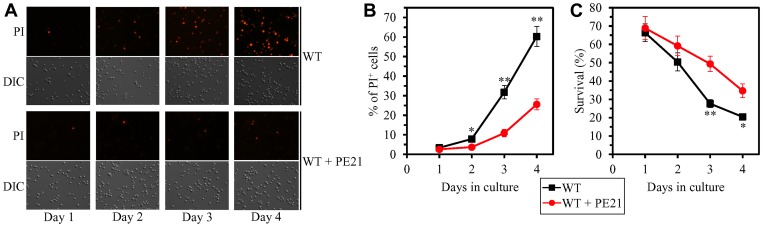
PE21 delays an age-related onset of necrotic death in yeast cells, decelerates the progression of the necrotic cell death process, and makes yeast less susceptible to a liponecrotic mode of regulated cell death (RCD). WT cells were cultured in the synthetic minimal YNB medium initially containing 2% glucose with 0.1% PE21 or without it. (**A**) Cells recovered on different days of culturing with or without PE21 were visualized using the differential interference contrast (DIC) microscopy and stained with propidium iodide (PI) as described in Materials and Methods. PI positive staining identifies cells that are permeable to PI because their plasma membranes have been damaged. Such loss of plasma membrane integrity is characteristic of necrotic cell death. (**B**) Percentage of cells displaying PI positive staining, a hallmark event of necrotic cell death. Images like the representative images shown in (**A**) were quantitated. Data are presented as means ± SEM (*n = *3; ^*^ *p < *0.05; ^**^ *p < *0.01). Data for the WT strain cultured with PE21 are replicated in Supplementary Figure 4A–4H. (**C**) Clonogenic survival of cells recovered on different days of culturing with or without PE21 and then exposed for 2 h to 0.1 mM palmitoleic acid (POA) as described in Materials and Methods. POA is a monounsaturated form of FFA that triggers a liponecrotic mode of RCD. Data are presented as means ± SEM (*n = *3; ^*^ *p < *0.05; ^**^ *p < *0.01). Data for the WT strain cultured with PE21 are replicated in Supplementary Figure 5A–5H.

Our hypothesis on the first mechanism suggests that, because PE21 maintains FFA concentration below the toxic threshold, it may weaken an age-related form of FFA-driven liponecrotic RCD (Figure 2). To test this suggestion, we first used live-cell fluorescence microscopy with propidium iodide (PI) to examine if PE21 can influence the age-related onset and/or progression of this mode of necrotic RCD in WT and mutant strains. PI positive staining is characteristic of necrotic RCD because PI is a stain used to visualize the loss of PM integrity, a hallmark event of necrotic RCD in yeast [11, 150–155, 219]. We found the following: 1) in WT cells, PE21 postpones the onset of necrosis since day 2 of culturing and decelerates the progression of necrotic RCD after that (Figure 7A and 7B); 2) since day 3 of culturing with PE21, the *faa1Δ*, *faa4Δ*, *ale1Δ* and *slc1Δ* mutations significantly increase the percentage of cells displaying PI positive staining typical of necrotic RCD (Supplementary Figure 4A–4D and 4J); and 3) since day 3 of culturing with PE21, the *tgl1Δ*, *tgl3Δ*, *tgl4Δ* and *tgl5Δ* mutations decrease the percentage of cells exhibiting PI positive staining characteristic of necrotic RCD (Supplementary Figure 4E–4H and 4J). Our comparison of the maximum percentage of cells displaying PI positive staining (which was observed in WT, *faa1Δ*, *faa4Δ*, *ale1Δ*, *slc1Δ*, *tgl1Δ*, *tgl3Δ*, *tgl4Δ* and *tgl5Δ* cells recovered on day 4 of culturing with PE21) and the highest intracellular concentration of FFA in these cells has revealed that the Pearson’s *r* value for the correlation between these two compared variables is more than 0.9 (Supplementary Figure 4I). Hence, the percentage of cells undergoing necrotic RCD has a very high positive correlation [218] and, therefore, directly correlates with FFA concentration in the yeast cell. This finding supports our assumption that PE21 delays the age-related onset of necrotic RCD and slows down the progression of this mode of RCD because it allows to sustain FFA concentration below the toxic threshold.

We then investigated if PE21 can affect the susceptibilities of WT and mutant strains to liponecrotic RCD. This mode of age-related RCD is known to be initiated in response to a brief exposure of yeast cells to exogenous FFA [52, 150, 151, 153]. The extent of liponecrotic RCD was measured as a decline in clonogenic survival of yeast cells that were treated for 2 h with a monounsaturated form of FFA called palmitoleic acid (POA). We found the following: 1) PE21 decreases the susceptibility of WT cells to liponecrotic RCD since day 2 of culturing (Figure 7C); 2) since day 2 of culturing with PE21, the *faa1Δ*, *faa4Δ*, *ale1Δ* and *slc1Δ* mutations make yeast cells more sensitive to liponecrotic RCD (Supplementary Figure 5A–5D and 5J); and 3) since day 3 of culturing with PE21, the *tgl1Δ*, *tgl3Δ*, *tgl4Δ* and *tgl5Δ* mutations make yeast cells more resistant to liponecrotic RCD (Supplementary Figure 5E–5H and 5J). Our comparison of the minimum percentage of clonogenic survival of POA-treated cells (which was observed in WT, *faa1Δ*, *faa4Δ*, *ale1Δ*, *slc1Δ*, *tgl1Δ*, *tgl3Δ*, *tgl4Δ* and *tgl5Δ* cells recovered on day 4 of culturing with PE21) and the highest intracellular concentration of FFA in these cells has shown that the Pearson’s *r* value for the correlation between these two compared variables is less than - 0.9 (Supplementary Figure 5I). We therefore have inferred that the resistance of yeast cells to liponecrotic RCD has a very high negative correlation [218] and, thus, inversely correlates with the intracellular concentration of FFA in the yeast cell. This observation indicates that PE21 makes yeast cells less vulnerable to liponecrotic RCD by allowing to lower FFA concentration.

Altogether, the above findings validate our hypothesis on the first mechanism through which PE21 decelerates yeast chronological aging and prolongs yeast CLS (Figure 2). In this mechanism, PE21 decreases the risk of aging-associated liponecrotic RCD and increases the chance of elderly cells to survive because PE21 enables yeast cells to maintain FFA concentration below the toxic threshold.

### PE21 causes global remodeling of the cellular proteome in an age-related manner

To make a first step towards testing our hypothesis on the second and third mechanisms of yeast longevity extension by PE21, we wanted to get a broader view of cellular processes that are affected by PE21 in yeast. We therefore used quantitative mass spectrometry to compare the cellular proteomes of WT yeast cultured in the presence of PE21 or in its absence.

**Figure 8:**
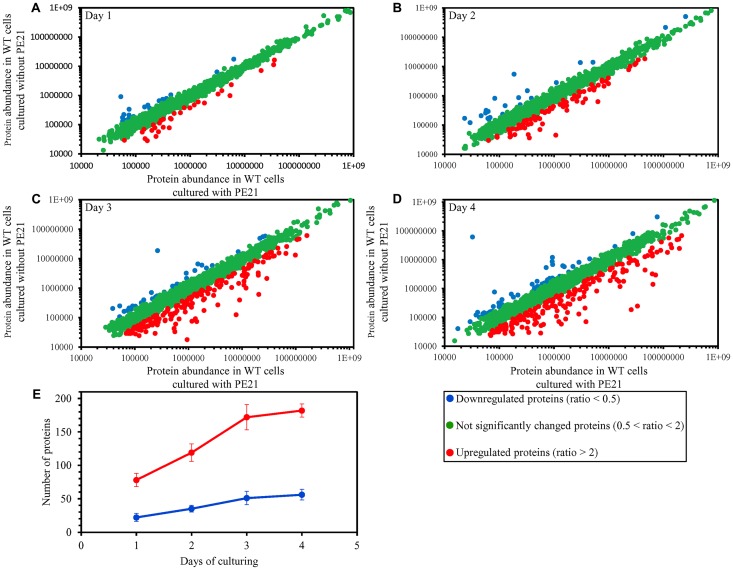
PE21 causes changes in the relative concentrations of many cellular proteins in an age-related manner. WT cells were cultured in the synthetic minimal YNB medium initially containing 2% glucose with 0.1% PE21 or without it. Cells were recovered on days 1, 2, 3 and 4 of culturing. Mass spectrometry-based identification and quantitation of proteins recovered from these cells, and the calculation of the relative abundance of cellular proteins in a pair of analyzed datasets (i. e. in the datasets of age-matched WT cells cultured with or without PE21), were performed as described in Materials and Methods. (**A**–**D**) Scatter plots comparing the relative abundance of cellular proteins between specified datasets were plotted on a log-log scale spanning six orders of magnitude. (**E**) The total number of proteins that are upregulated (displayed in red) or downregulated (displayed in blue) in response to the treatment with PE21. Data are presented as means ± SEM (*n = *2).

We found that PE21 causes changes in the relative concentrations of many cellular proteins in WT yeast (Figure 8A–8D). We also noticed that the total number of proteins upregulated or downregulated in WT cells in response to PE21 exposure is increased with the chronological age of these cells (Figure 8E).

We used principal component analysis (PCA) to compare the proteome of WT cells cultured in the presence of PE21 to the proteome of age-matched WT cells cultured in the absence of PE21; the cells were recovered on days 1, 2, 3 or 4 of culturing. Our PCA revealed that PE21 elicits a distinct cellular proteome profile in WT yeast that significantly differs from a profile of the cellular proteome in WT yeast cultured in the absence of PE21 (Supplementary Figure 6A–6D). This distinct PE21-driven cellular proteome profile was observed in WT cells recovered on any of the four days of culturing (Supplementary Figure 6A–6D). The sample with PE21 and the reference without PE21 were separated farthest from each other (i. e. 30 and 55 units of distance between final cluster centers along the PC1 and PC2 axes, respectively) in case of the cellular proteomes of chronologically old WT cells recovered on day 4 of culturing (Supplementary Figure 6D). Although the sample with PE21 and the reference without PE21 were also separated from each other in case of the cellular proteomes of chronologically young WT cells recovered on day 1 of culturing, they were clustered much closer to each other (i. e. 12 and 4 units of distance between final cluster centers along the PC1 and PC2 axes, respectively) than those of chronologically old WT cells recovered on day 4 (compare Supplementary Figure 6A and 6D).

In sum, these findings indicate that PE21 prompts the establishment of a distinct cellular proteome profile in WT yeast and that the efficiency with which PE21 changes this profile is gradually increased with the chronological age of WT yeast.

### PE21 prolongs longevity of chronologically aging yeast in part because it promotes UPR^ER^

Our hypothesis on the second mechanism through which PE21 may extend longevity of chronologically aging yeast assumes that, because PE21 alters the ER lipidome, it stimulates the UPR^ER^ system (Figure 2); such PE21-driven stimulation of UPR^ER^ may be responsible, in part, for the observed abilities of PE21 to slow down an age-related decline in protein, lipid and nucleic acid homeostasis and to decelerate an aging-associated weakening of cell resistance to oxidative and thermal stresses [55]. In support of this assumption, we found that in WT yeast PE21 alters the relative concentrations of various cellular proteins whose upregulation or downregulation is indispensable for the restoration and maintenance of cellular homeostasis because it is essential for a proper control of the UPR^ER^ system.

**Figure 9:**
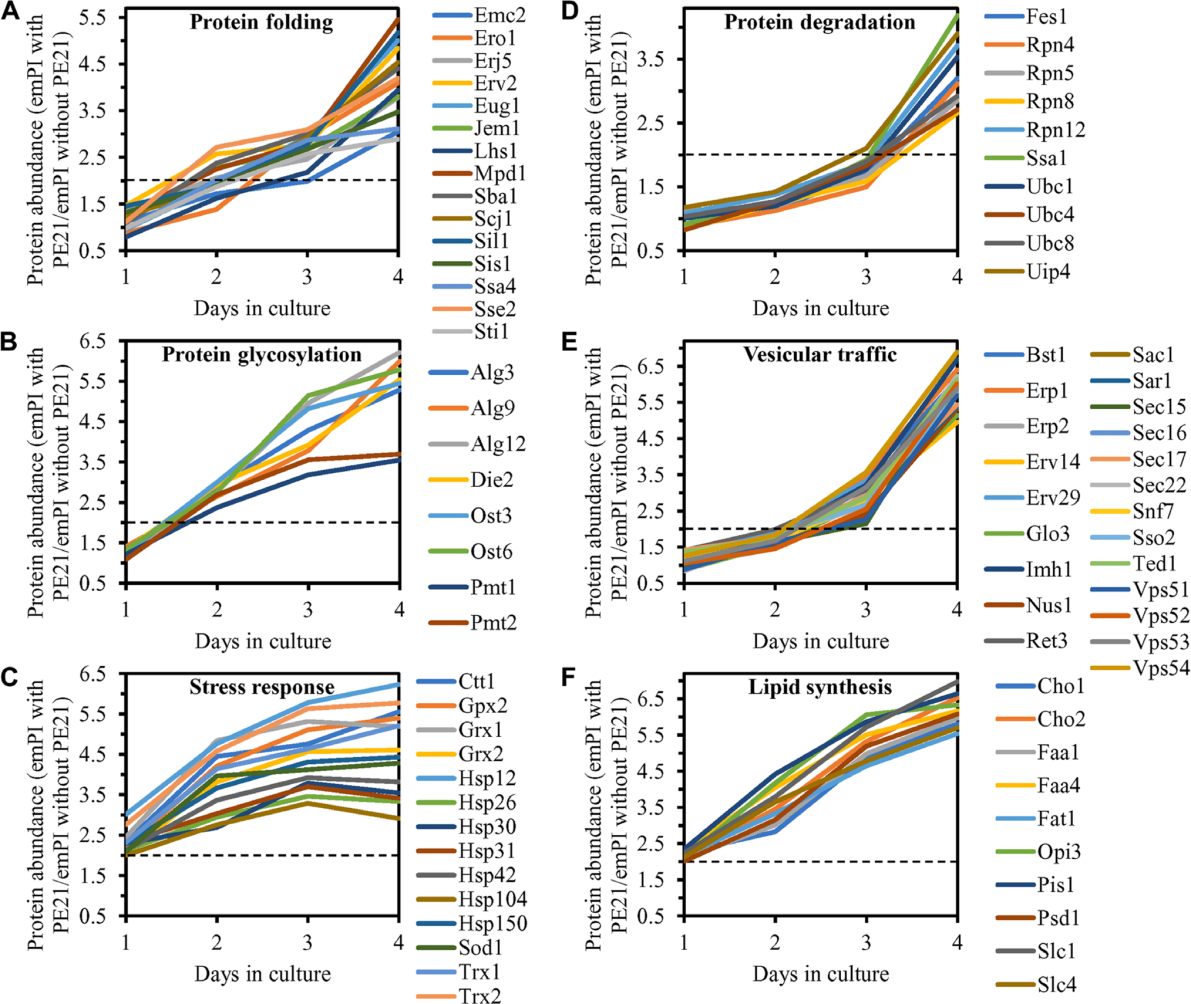
PE21 increases the abundance of six classes of cellular proteins known to be upregulated during the UPR^ER^ response in yeast. WT cells were cultured in the synthetic minimal YNB medium initially containing 2% glucose with 0.1% PE21 or without it. Cells were recovered on days 1, 2, 3 and 4 of culturing. Mass spectrometry-based identification and quantitation of proteins recovered from these cells, and the calculation of the relative abundance of cellular proteins in a pair of analyzed datasets (i. e. in the datasets of age-matched WT cells cultured with or without PE21), were performed as described in Materials and Methods. (**A**–**F**) Relative levels of proteins in WT cells cultured with PE21 (fold difference relative to those in WT cells cultured without PE21) are shown. These proteins include the following ones: chaperones involved in protein folding and assembly in the endoplasmic reticulum or the cytosol (**A**), proteins that catalyze *N*-linked protein glycosylation or *O*-linked protein mannosylation in the endoplasmic reticulum (**B**), stress response proteins that prevent and/or repair an oxidative or thermal damage to proteins in the endoplasmic reticulum, cytosol and plasma membrane (**C**), proteins involved in the degradation of improperly folded proteins accumulated in the endoplasmic reticulum via the ubiquitin-proteasome pathway (**D**), proteins implicated in vesicular traffic from the endoplasmic reticulum throughout the secretory pathway (**E**), and proteins that catalyze the synthesis of lipids in the endoplasmic reticulum and mitochondria (**F**). The 2-fold increase in the ratio “protein abundance with PE21/protein abundance without PE21” is shown by a dotted line. Data are presented as mean values of 2 independent experiments. Abbreviation: emPAI, the exponentially modified protein abundance index, a measure of the relative abundance of cellular proteins in a pair of analyzed datasets.

We noticed that PE21 increases the abundance of many cellular proteins known to be upregulated during the UPR^ER^ response in yeast [159, 161, 164, 181, 188, 189, 193, 202, 220]. The cellular proteins upregulated by PE21 included the following ones: 1) chaperones involved in protein folding and assembly in the ER or the cytosol (Figure 9A); 2) proteins that catalyze *N*-linked protein glycosylation or *O*-linked protein mannosylation in the ER (Figure 9B); 3) stress response proteins that prevent and/or repair an oxidative or thermal damage to proteins and/or lipids in the ER, mitochondria, cytosol and/or PM (Figure 9C); 4) protein components of the ubiquitin-proteasome system involved in the degradation of improperly folded proteins that are accumulated in the ER and then exported to the cytosol (Figure 9D); 5) proteins implicated in vesicular traffic from the ER throughout the secretory pathway (Figure 9E); and 6) proteins that catalyze lipid synthesis in the ER and mitochondria (Figure 9F). Of note, the PE21-dependent increase in the abundance of enzymes catalyzing the synthesis of PA (i. e. Faa1, Faa4, Fat1, Slc1 and Slc4), PS (i. e. Cho1), PE (i. e. Psd1), PC (i. e. Cho2 and Opi3) and PI (i. e. Pis1) in the ER and mitochondria (Figure 9F and Supplementary Figure 1) can satisfactorily explain the PE21-dependent rise in the concentrations of these glycerophospholipids (Figure 1C–1G). It needs to be emphasized that, as we found, a single-gene-deletion mutation eliminating Faa1, Faa4 or Slc1 causes a significant decline in longevity-extending efficiency of PE21 (Figures 3A–3D, 3F–3I and 4F–4I). Thus, PE21 extends longevity of chronologically aging yeast in part because it stimulates a branch of the UPR^ER^ network responsible for glycerophospholipid synthesis in the ER. As discussed below in this section, the PE21-driven upregulation of other UPR^ER^ network branches also contributes to the PE21-dependent extension of yeast CLS.

We also found that PE21 decreases the abundance of cellular proteins known to be downregulated during the UPR^ER^ response in yeast [159, 161, 164, 181, 188, 189, 193, 202, 220]. These proteins have been implicated in ribosome assembly, tRNA synthesis and protein translation in the cytosol (Supplementary Figure 7).

**Figure 10:**
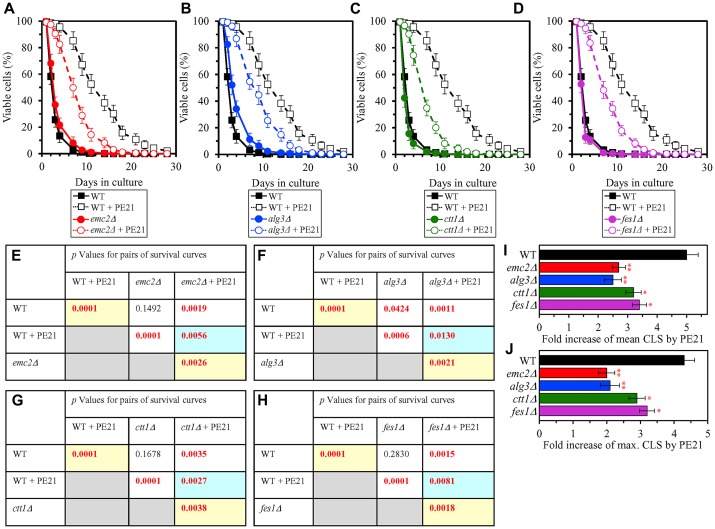
Single-gene-deletion mutations eliminating proteins that are upregulated by both PE21 and UPR^ER^ stimuli decrease the efficiency with which PE21 extends yeast longevity. WT cells and mutant cells carrying a single-gene-deletion mutation eliminating either Emc2, Alg3, Ctt1 or Fes1 were cultured in the synthetic minimal YNB medium initially containing 2% glucose with 0.1% PE21 or without it. (**A**–**D**) Survival curves of the chronologically aging WT and *emc2Δ* (**A**), WT and *alg3Δ* (**B**), WT and *ctt1Δ* (**C**) or WT and *fes1Δ* (**D**) strains are shown. Data are presented as means ± SEM (*n = *3). Data for the WT strain cultured with or without PE21 are replicated in the graphs of (**A**) and (**F**) and Figures 3A, 3F, 4A, 4F, 5A, 5F, 6A, 6F, 11A–11C, 14A–14D, 15A–15D. (**E**–**H**) *p* Values for different pairs of survival curves of the WT and *emc2Δ* (**E**), WT and *alg3Δ* (**F**), WT and *ctt1Δ* (**G**) or WT and *fes1Δ* (**H**) strains cultured with or without PE21. Survival curves shown in (**A**–**D**) (respectively) were compared. Two survival curves were considered statistically different if the *p* value was less than 0.05. The *p* values for comparing pairs of survival curves using the logrank test were calculated as described in Materials and Methods. The *p* values displayed on a yellow color background indicate that PE21 statistically significantly prolongs the CLS of the WT (**E**–**H**), *emc2Δ* (**E**), *alg3Δ* (**F**), *ctt1Δ* (**G**) and *fes1Δ* (**H**) strains. The *p* values displayed on a blue color background indicate that PE21 prolongs the CLS of the *emc2Δ* (**E**), *alg3Δ* (**F**), *ctt1Δ* (**G**) and *fes1Δ* (**H**) strains to a lower extent than that of the WT strain. (**I**, **J**) Survival curves shown in (**A**–**D**) were used to calculate the fold of increase of the mean (**I**) and maximum (**J**) CLS by PE21 for the WT, *emc2Δ*, *alg3Δ*, *ctt1Δ* and *fes1Δ* strains. Data are presented as means ± SEM (*n = *3; ^*^ *p < *0.05; ^**^ *p < *0.01).

**Figure 11:**
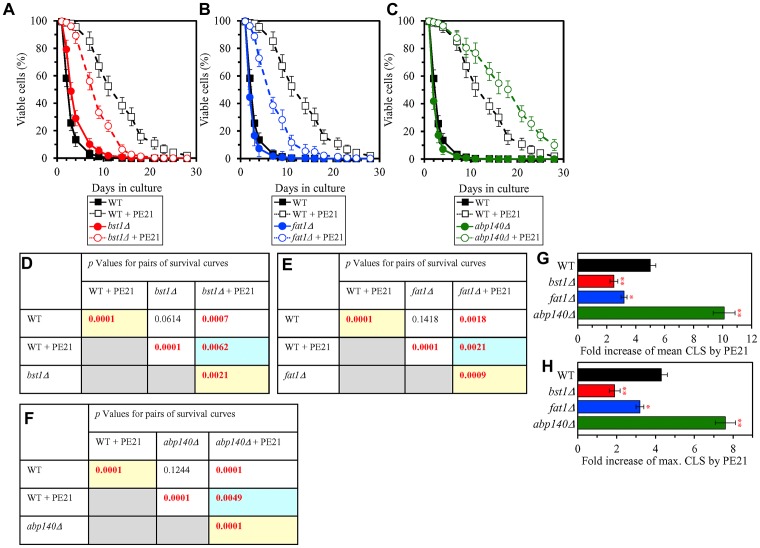
Single-gene-deletion mutations eliminating proteins that are upregulated by both PE21 and UPR^ER^ stimuli decrease the efficiency with which PE21 extends yeast longevity, while a single-gene-deletion mutation eliminating a protein that is downregulated by both PE21 and UPR^ER^ stimuli increases such efficiency. WT cells and mutant cells carrying a single-gene-deletion mutation eliminating either Bst1, Fat1 or Abp140 were cultured in the synthetic minimal YNB medium initially containing 2% glucose with 0.1% PE21 or without it. (**A**–**C**) Survival curves of the chronologically aging WT and *bst1Δ* (**A**), WT and *fat1Δ* (**B**) or WT and *abp140Δ* (**C**) strains are shown. Data are presented as means ± SEM (*n = *3). Data for the WT strain cultured with or without PE21 are replicated in the graphs of (**A**) and (**F**) and Figures 3A, 3F, 4A, 4F, 5A, 5F, 6A, 6F, 10A–10D, 14A–14D, 15A–15D. (**D**–**F**) *p* Values for different pairs of survival curves of the WT and *bst1Δ* (**D**), WT and *fat1Δ* (**E**) or WT and *abp140Δ* (**F**) strains cultured with or without PE21. Survival curves shown in **A**–**C** (respectively) were compared. Two survival curves were considered statistically different if the *p* value was less than 0.05. The *p* values for comparing pairs of survival curves using the logrank test were calculated as described in Materials and Methods. The *p* values displayed on a yellow color background indicate that PE21 statistically significantly prolongs the CLS of the WT (**D**–**F**), *bst1Δ* (**D**), *fat1Δ* (**E**) and *abp140Δ* (**F**) strains. The *p* values displayed on a blue color background indicate the following: 1) PE21 prolongs the CLS of the *bst11Δ* (**D**) and *fat1Δ* (**E**) strains to a lower extent than that of the WT strain; and 2) PE21 prolongs the CLS of the *fat1Δ* strain (**F**) to a higher extent than that of the WT strain. (**G**, **H**) Survival curves shown in (**A**–**C**) were used to calculate the fold of increase of the mean (**G**) and maximum (**H**) CLS by PE21 for the WT, *bst1Δ*, *fat1Δ* and *abp140Δ* strains. Data are presented as means ± SEM (*n = *3; ^*^ *p < *0.05; ^**^ *p < *0.01).

We thought that cellular proteins upregulated by both PE21 and UPR^ER^ stimuli may play essential roles in enabling aging delay by PE21. We therefore hypothesized that single-gene-deletion mutations eliminating such proteins may decrease the aging-delaying (geroprotective) efficiency of PE21. In support of our hypothesis, yeast mutants that lack the following proteins upregulated in a PE21- and UPR^ER^-dependent manner exhibited a statistically significant decline in the geroprotective efficiency of PE21: 1) Emc2, Erj5, Erv2 and Eug1, all of which are chaperones assisting in the folding and assembly of other proteins within the ER (Figure 10A, 10E, 10I and 10J for the *emc2Δ* mutant; Supplementary Figure 8A and 8B for the *erj5Δ*, *erv2Δ* and *eug1Δ* mutants); 2) Alg3, Alg12, Ost3 and Ost6, all of which are enzymes catalyzing *N*-linked protein glycosylation within the ER (Figure 10B, 10F, 10I and 10J for the *alg3Δ* mutant; Supplementary Figure 8C and 8D for the *alg12Δ*, *ost3Δ* and *ost6Δ* mutants); 3) Ctt1, Gpx2, Grx1 and Grx2, all of which are stress response proteins preventing and/or repairing an oxidative damage to proteins and/or lipids in the cytosol and mitochondria (Figure 10C, 10G, 10I and 10J for the *ctt1Δ* mutant; Supplementary Figure 8E and 8F for the *gpx2Δ*, *grx1Δ* and *grx2Δ* mutants); 4) Fes1, Rpn4, Ssa1 and Ubc8, all of which are components of the ubiquitin-proteasome pathway for the degradation of improperly folded proteins that amass in the ER (Figure 10D, 10H, 10I and 10J for the *fes1Δ* mutant; Supplementary Figure 8G and 8H for the *rpn4Δ*, *ssa1Δ* and *ubc8Δ* mutants); 5) Bst1, Erp1, Erp2 and Erv29, all of which are proteins involved in vesicular traffic from the ER to the Golgi apparatus (Figure 11A, 11D, 11G and 11H for the *bst1Δ* mutant; Supplementary Figure 8I and 8J for the *erp1Δ*, *erp2Δ* and *erv29Δ* mutants); and 6) Fat1 and Opi3, both being implicated in glycerophospholipid synthesis within the ER (Figure 11B, 11E, 11G and 11H for the *fat1Δ* mutant; Supplementary Figure 8K and 8L for the *opi3Δ* mutant; as indicated above and as shown in Figure 3A–3D, 3F–3I and 4F–4I, the geroprotective efficiency of PE21 is also decreased in yeast mutants lacking other glycerophospholipid synthesis enzymes that are upregulated in a PE21- and UPR^ER^-dependent manner).

We also thought that cellular proteins downregulated by both PE21 and UPR^ER^ stimuli may be important for impeding aging delay by PE21. Our hypothesis was therefore that single-gene-deletion mutations eliminating such proteins may increase the geroprotective efficiency of PE21. In support of our hypothesis, yeast mutants that lack Abp140, Acl4, Caf20 and Gis2 displayed a statistically significant rise in the geroprotective efficiency of PE21 (Figure 11C, 11F, 11G and 11H for the *abp140Δ* mutant; Supplementary Figure 8M and 8N for the *acl4Δ*, *caf20Δ* and *gis2Δ* mutants). These proteins play essential roles in ribosome assembly, tRNA synthesis and protein translation in the cytosol [221–228] and are downregulated by both PE21 (Supplementary Figure 7) and UPR^ER^ stimuli [159, 161, 164, 181, 188, 189, 193, 202, 220].

Taken together, the above findings validate our hypothesis on the second mechanism by which PE21 delays yeast chronological aging and extends yeast CLS (Figure 2). In this mechanism, PE21 stimulates the UPR^ER^ system, thus slowing an age-related decline in protein and lipid homeostasis and decelerating an aging-associated deterioration of cell resistance to oxidative and thermal stresses.

### PE21 extends longevity of chronologically aging yeast in part because it rearranges processes within mitochondria, thus changing functionality of these organelles

Our hypothesis on the third mechanism through which PE21 may extend longevity of chronologically aging yeast posits that, because PE21 alters the membrane lipidome of mitochondria, it may reorganize processes within these organelles to change mitochondrial functionality (Figure 2). In our hypothesis, such PE21-dependent change in mitochondrial functionality may be responsible, in part, for the observed ability of PE21 to amend the temporal dynamics of age-related alterations in mitochondrial respiration, mitochondrial membrane potential and mitochondrial ROS production [55]. In support of our hypothesis, we found that in WT yeast PE21 elicits changes the relative concentrations of various mitochondrial proteins implicated in key aspects of mitochondrial functionality.

**Figure 12:**
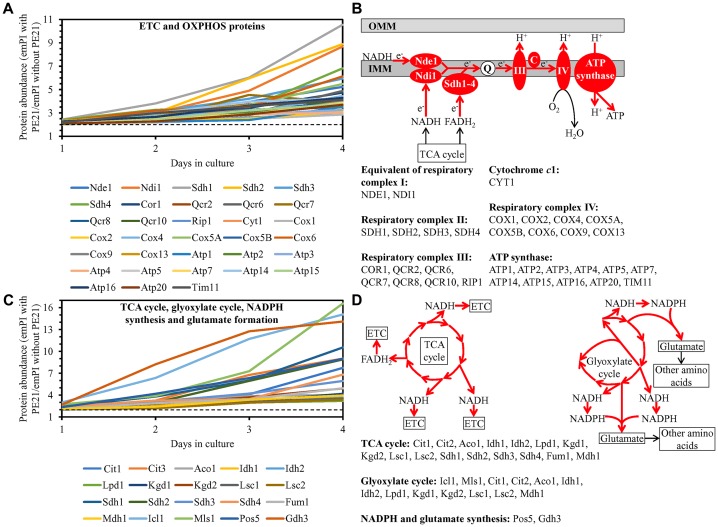
PE21 increases the abundance of proteins involved in the mitochondrial electron transport chain (ETC), oxidative phosphorylation (OXPHOS) system, tricarboxylic acid (TCA) cycle (TCA), glyoxylate cycle, NADPH synthesis and glutamate formation. WT cells were cultured in the synthetic minimal YNB medium initially containing 2% glucose with 0.1% PE21 or without it. Cells were recovered on days 1, 2, 3 and 4 of culturing. Mass spectrometry-based identification and quantitation of proteins recovered from these cells, and the calculation of the relative abundance of cellular proteins in a pair of analyzed datasets (i. e. in the datasets of age-matched WT cells cultured with or without PE21), were performed as described in Materials and Methods. (**A**, **C**) Relative levels of proteins in WT cells cultured with PE21 (fold difference relative to those in WT cells cultured without PE21) are shown. The 2-fold increase in the ratio “protein abundance with PE21/protein abundance without PE21” is shown by a dotted line. Data are presented as mean values of 2 independent experiments. (**B**) Protein components of the mitochondrial ETC and OXPHOS system whose concentrations are increased in yeast cells cultured in the presence of PE21 are displayed in red color. The names of these protein components are provided. Red arrows denote the reactions of electron transport, proton transfer across the inner mitochondrial membrane (IMM) and ATP synthesis that are accelerated due to a PE21-dependent upregulation of protein components of the mitochondrial ETC and OXPHOS system. (**D**) Red arrows indicate the reactions of the TCA cycle, glyoxylate cycle, NADPH synthesis and glutamate formation that are accelerated because of a PE21-dependent upregulation of protein components involved in these metabolic processes within mitochondria. The names of these protein components are provided. Other abbreviations: C, cytochrome *c*; emPAI, the exponentially modified protein abundance index, a measure of the relative abundance of cellular proteins in a pair of analyzed datasets; OMM, outer mitochondrial membrane; Q, ubiquinone (coenzyme Q); III and IV, respiratory complexes III and IV of the mitochondrial ETC.

**Figure 13:**
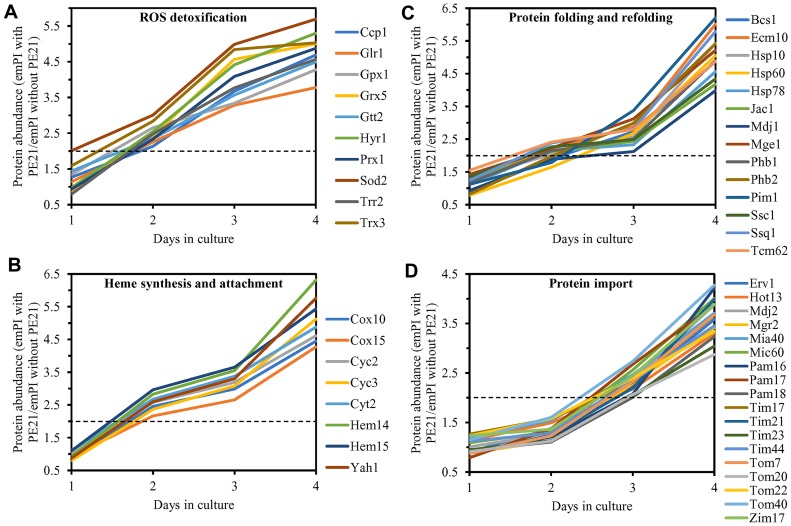
PE21 increases the abundance of mitochondrial proteins implicated in ROS detoxification, heme synthesis and protein attachment, protein folding and refolding, and protein import into mitochondria. WT cells were cultured in the synthetic minimal YNB medium initially containing 2% glucose with 0.1% PE21 or without it. Cells were recovered on days 1, 2, 3 and 4 of culturing. Mass spectrometry-based identification and quantitation of proteins recovered from these cells, and the calculation of the relative abundance of cellular proteins in a pair of analyzed datasets (i. e. in the datasets of age-matched WT cells cultured with or without PE21), were performed as described in Materials and Methods. Relative levels of proteins in WT cells cultured with PE21 (fold difference relative to those in WT cells cultured without PE21) are shown. These proteins include the following ones: mitochondrial proteins involved in ROS detoxification and oxidative stress protection (**A**), enzymes catalyzing heme synthesis and proteins facilitating heme attachment to other proteins (**B**), chaperones assisting in the folding and refolding of other mitochondrial proteins (**C**), components of the mitochondrial protein import machinery (**D**). The 2-fold increase in the ratio “protein abundance with PE21/protein abundance without PE21” is shown by a dotted line. Data are presented as mean values of 2 independent experiments. Abbreviation: emPAI, the exponentially modified protein abundance index, a measure of the relative abundance of cellular proteins in a pair of analyzed datasets.

We noted that PE21 increases the abundance of proteins involved in the following mitochondrial processes: 1) the electron transport chain (ETC) and oxidative phosphorylation (OXPHOS) system in mitochondria (Figure 12A and 12B); 2) the mitochondrial tricarboxylic acid (TCA) cycle, glyoxylate cycle, synthesis of NADPH and formation of glutamate (which is a common precursor for the synthesis of other amino acids, folates and glutathione in mitochondria and the cytosol [229]) (Figure 12C and 12D); 3) ROS detoxification and oxidative stress protection (Figure 13A); 4) the synthesis of heme and its attachment to proteins (Figure 13B); 5) protein folding and refolding (Figure 13C); and 6) protein import into mitochondria (Figure 13D).

We also noted that PE21 decreases the abundance of mitochondrial proteins implicated in the division (fission) of mitochondria as well as in RNA synthesis, processing and translation within these organelles (Supplementary Figure 9).

**Figure 14:**
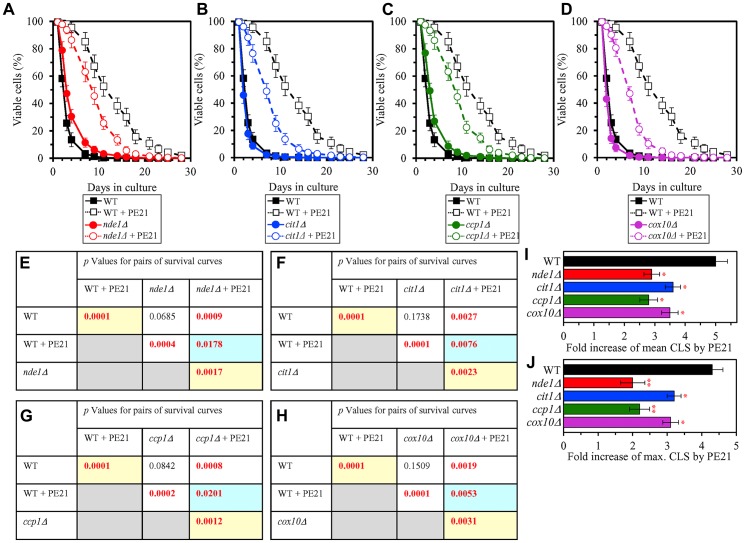
Single-gene-deletion mutations eliminating mitochondrial proteins that are upregulated by PE21 reduce the geroprotective potential of PE21. WT cells and mutant cells carrying a single-gene-deletion mutation eliminating either Nde1, Cit1, Ccp1 or Cox10 were cultured in the synthetic minimal YNB medium initially containing 2% glucose with 0.1% PE21 or without it. (**A**–**D**) Survival curves of the chronologically aging WT and *nde1Δ* (**A**), WT and *cit1Δ* (**B**), WT and *ccp1Δ* (**C**) or WT and *cox10Δ* (**D**) strains are shown. Data are presented as means ± SEM (*n = *3). Data for the WT strain cultured with or without PE21 replicated in the graphs of (**A**) and (**F**) And Figures 3A, 3F, 4A, 4F, 5A, 5F, 6A, 6F, 10A–10D, 11A–11C, 15A–15D. (**E**–**H**) *p* Values for different pairs of survival curves of the WT and *nde1Δ* (**E**), WT and *cit1Δ* (**F**), WT and *ccp1Δ* (**G**) or WT and *cox10Δ* (**H**) strains cultured with or without PE21. Survival curves shown in **A**–**D** (respectively) were compared. Two survival curves were considered statistically different if the *p* value was less than 0.05. The *p* values for comparing pairs of survival curves using the logrank test were calculated as described in Materials and Methods. The *p* values displayed on a yellow color background indicate that PE21 statistically significantly prolongs the CLS of the WT (**E–H**), *nde1Δ* (**E**), *cit1Δ* (**F**), *ccp1Δ* (**G**) and *cox10Δ* (**H**) strains. The *p* values displayed on a blue color background indicate that PE21 prolongs the CLS of the *nde1Δ* (**E**), *cit1Δ* (**F**), *ccp1Δ* (**G**) and *cox10Δ* (**H**) strains to a lower extent than that of the WT strain. (**I**, **J**) Survival curves shown in (**A**–**D**) were used to calculate the fold of increase of the mean (**I**) and maximum (**J**) CLS by PE21 for the WT, *nde1Δ*, *cit1Δ*, *ccp1Δ* and *cox10Δ* strains. Data are presented as means ± SEM (*n = *3; ^*^ *p < *0.05; ^**^ *p < *0.01).

**Figure 15:**
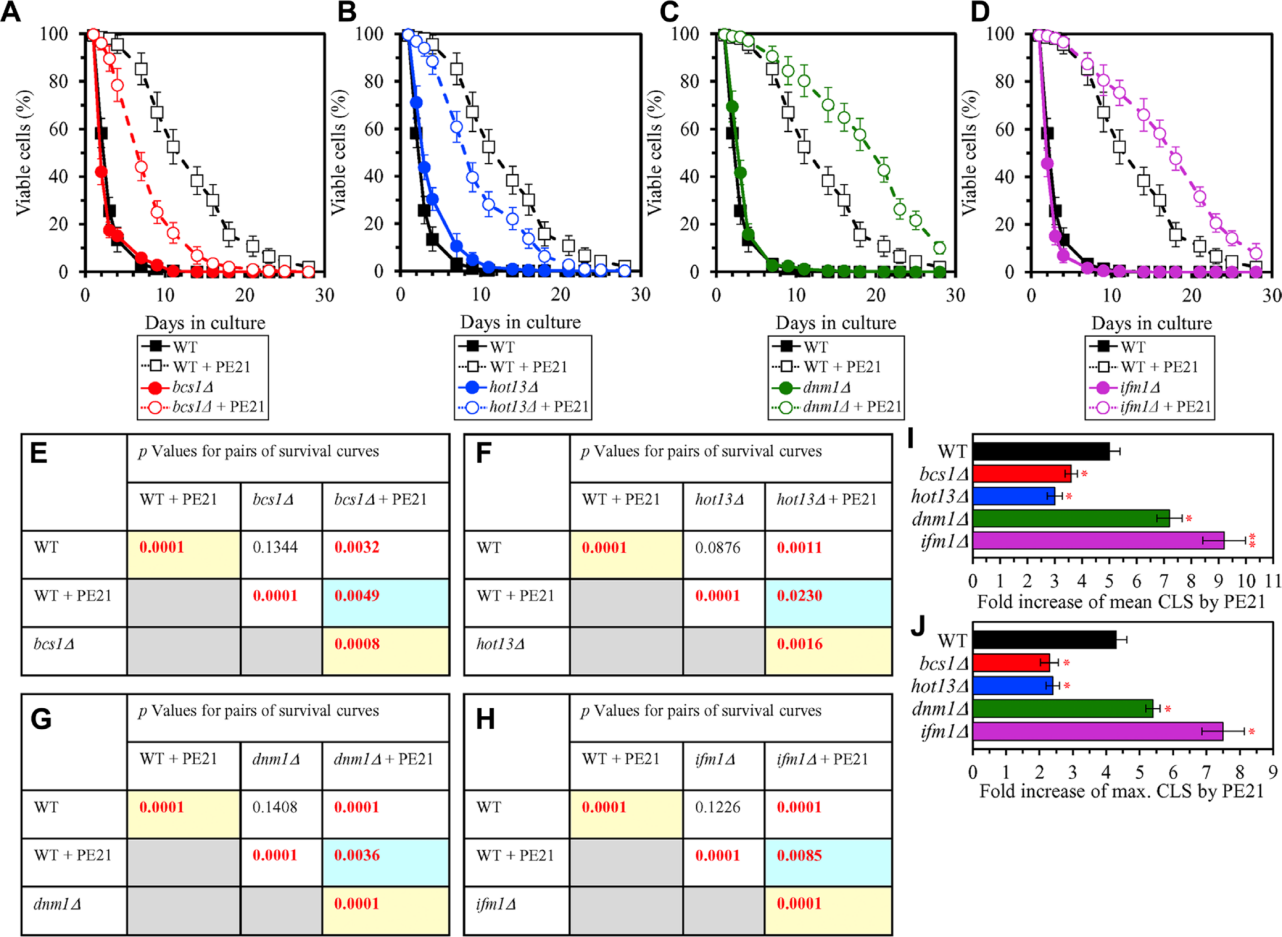
Single-gene-deletion mutations eliminating mitochondrial proteins that are upregulated by PE21 decrease the geroprotective potential of PE21, whereas single-gene-deletion mutations eliminating mitochondrial proteins that are downregulated by PE21 increases such potential. WT cells and mutant cells carrying a single-gene-deletion mutation eliminating either Bcs1, Hot13, Dnm1 or Ifm1 were cultured in the synthetic minimal YNB medium initially containing 2% glucose with 0.1% PE21 or without it. (**A**–**D**) Survival curves of the chronologically aging WT and *bcs1Δ* (**A**), WT and *hot13Δ* (**B**), WT and *dnm1Δ* (**C**) or WT and *ifm1Δ* (**D**) strains are shown. Data are presented as means ± SEM (*n = *3). Data for the WT strain cultured with or without PE21 are replicated in the graphs of (**A**) and (**F**) and Figures 3A, 3F, 4A, 4F, 5A, 5F, 6A, 6F, 10A–10D, 11A–11C, 14A–14D. (**E**–**H**) *p* Values for different pairs of survival curves of the WT and *bcs1Δ* (**E**), WT and *hot13Δ* (**F**), WT and *dnm1Δ* (**G**) or WT and *ifm1Δ* (**H**) strains cultured with or without PE21. Survival curves shown in **A**–**D** (respectively) were compared. Two survival curves were considered statistically different if the *p* value was less than 0.05. The *p* values for comparing pairs of survival curves using the logrank test were calculated as described in Materials and Methods. The *p* values displayed on a yellow color background indicate that PE21 statistically significantly prolongs the CLS of the WT (**E**–**H**), *bcs1Δ* (**E**), *hot13Δ* (**F**), *dnm1Δ* (**G**) and *ifm1Δ* (**H**) strains. The *p* values displayed on a blue color background indicate that PE21 prolongs the CLS of the *bcs1Δ* (**E**), *hot13Δ* (**F**), *dnm1Δ* (**G**) and *ifm1Δ* (**H**) strains to a lower extent than that of the WT strain. (**I**, **J**) Survival curves shown in (**A**–**D**) were used to calculate the fold of increase of the mean (**I**) and maximum (**J**) CLS by PE21 for the WT, *bcs1Δ*, *hot13Δ*, *dnm1Δ* and *ifm1Δ* strains. Data are presented as means ± SEM (*n = *3; ^*^ *p < *0.05; ^**^ *p < *0.01).

It is conceivable that those mitochondrial proteins that are upregulated by PE21 may be essential contributors to a PE21-dependent delay of yeast chronological aging. If this assumption is correct, then single-gene-deletion mutations that eliminate such mitochondrial proteins may reduce the geroprotective potential of PE21. We found that this assumption holds true, as PE21 is a significantly less efficient geroprotective agent for single-gene-deletion mutants that lack the following PE21-inducible mitochondrial proteins: 1) components of the ETC and OXPHOS system in mitochondria (Figure 14A, 14E, 14I and 14J for the *nde1Δ* mutant; Supplementary Figure 10A and 10B for the *ndi1Δ*, *sdh1Δ* and *sdh2Δ* mutants); 2) enzymes of the TCA and glyoxylate cycles in mitochondria (Figure 14B, 14F, 14I and 14J for the *cit1Δ* mutant; Supplementary Figure 10C and 10D for the *cit3Δ*, *idh1Δ* and *idh2Δ* mutants); 3) enzymes implicated in ROS detoxification and oxidative stress protection in mitochondria (Figure 14C, 14G, 14I and 14J for the *ccp1Δ* mutant; Supplementary Figure 10E and 10F for the *glr1Δ*, *gpx1Δ* and *gtt2Δ* mutants); 4) proteins involved in the formation of heme and its attachment to other proteins in mitochondria (Figure 14D, 14H, 14I and 14J for the *cox10Δ* mutant; Supplementary Figure 10G and 10H for the *cox15Δ*, *cyc2Δ* and *cyc3Δ* mutants); 5) chaperones assisting in the folding and refolding of proteins within mitochondria (Figure 15A, 15E, 15I and 15J for the *bcs1Δ* mutant; Supplementary Figure 10I and 10J for the *ecm10Δ*, *hsp78Δ* and *mdj1Δ* mutants); and 6) components of the mitochondrial protein import machinery (Figure 15B, 15F, 15I and 15J for the *hot13Δ* mutant; Supplementary Figure 10K and 10L for the *mdj2Δ*, *mgr2Δ* and *mic60Δ* mutants).

It is also plausible that those mitochondrial proteins that are downregulated by PE21 may have important contributions to the suppression of a geroprotective action of PE21. One could therefore assume that single-gene-deletion mutations that eliminate such mitochondrial proteins may enhance the geroprotective potential of PE21. This assumption holds true, as we observed that PE21 is a significantly more efficient geroprotector for single-gene-deletion mutants that lack the following PE21-suppresible mitochondrial proteins: 1) components of the mitochondrial division (fission) machinery (Figure 15C, 15G, 15I and 15J for the *dnm1Δ* mutant; Supplementary Figure 10M and 10N for the *fis1Δ*, *mdm36Δ* and *mdv1Δ* mutants); and 2) proteins involved in RNA synthesis, processing and translation within mitochondria (Figure 15D, 15H, 15I and 15J for the *ifm1Δ* mutant; Supplementary Figure 10O and 10P for the *img1Δ*, *img2Δ* and *mef1Δ* mutants).

In sum, the above findings confirm our hypothesis on the third mechanism through which PE21 decelerates yeast chronological aging and prolongs yeast longevity (Figure 2). This mechanism consists in a PE21-driven remodeling of certain processes taking place within mitochondria and the resulting changes in functionality of these organelles.

## DISCUSSION

This study and previously published findings [52, 55, 56, 150, 151, 154, 155] validate our hypothesis on the existence of three different mechanisms through which PE21 can delay yeast chronological aging and extend yeast longevity. This hypothesis is described in the Results section and schematically depicted in Figure 2.

We found that PE21 activates these three different mechanisms of aging delay and longevity extension because it instigates specific changes in the concentrations of several lipid classes, as summarized below.

The first mechanism by which PE21 slows aging and extends longevity consists in the ability of PE21 to decrease the intracellular concentration of FFA (Figure 2). This allows PE21 to sustain FFA concentration below the toxic threshold, thus postponing the age-related onset of an FFA-dependent mode of liponecrotic RCD (Figure 2). The commitment of yeast to this mode of RCD in response to excessive FFA concentrations is caused by an augmentation of PM permeability for small molecules, a weakening of mitochondrial functionality, a significant increase in mitochondrial ROS production, an oxidative damage to different types of cellular organelles and the ensuing autophagic degradation of these organelles *en masse*, and an oxidative impairment of many cytosolic proteins that leads to a build-up of the dysfunctional, unfolded and aggregated forms of these proteins [32, 52, 137, 150–155]. Because PE21 decreases the risk of aging-associated liponecrotic RCD and increases the chance of elderly cells to survive, it decelerates chronological aging and prolongs longevity of *S. cerevisiae*.

The second mechanism through which PE21 delays aging and expands longevity consists in its ability to decrease the concentration of TAG and to increase the concentrations of all glycerophospholipid classes (i. e. PA, PC, PE, PI and PS) within the ER membrane (Figure 2). These PE21-dependent perturbations in the abundance of ER membrane lipids activate the UPR^ER^ system (Figure 2). The PE21-dependent activation of the UPR^ER^ system promotes chaperone-assisted protein folding and assembly within the ER, *N*-linked protein glycosylation in the ER, the ubiquitin/proteasome-dependent degradation of improperly folded proteins in the ER, vesicular protein traffic from the ER to the Golgi apparatus, glycerophospholipid synthesis within the ER, and the reparation of oxidative damage to proteins and lipids in the cytosol and mitochondria. The PE21-dependent activation of the UPR^ER^ system also suppresses ribosome assembly, tRNA synthesis and protein translation in the cytosol; such suppression is known to delay aging and extend longevity in evolutionarily distant eukaryotes [28, 30, 230–254]. All cellular processes that are promoted or suppressed by the PE21-inducible UPR^ER^ system are indispensable for the ability of PE21 to slow yeast chronological aging because they allow to decelerate an age-related decline in protein and lipid homeostasis and to delay an aging-associated deterioration of cell resistance to oxidative and thermal stresses.

The third mechanisms underlying aging delay and longevity extension by PE21 consists in the ability of PE21 to increase PS and PE concentrations and to decrease CL concentration in the mitochondrial membranes (Figure 2). These PE21-driven changes in the mitochondrial membrane lipidome alter mitochondrial functionality because they cause an upregulation or downregulation of many mitochondrial proteins, thereby reorganizing vital processes confined to mitochondria. Mitochondrial proteins that are upregulated in response to PE21 include components of the ETC and OXPHOS, enzymes that catalyze the TCA and glyoxylate cycles, proteins involved in ROS detoxification and oxidative stress protection, proteins implicated in the synthesis of heme and its attachment to other proteins, chaperones that assist in the folding and refolding of other proteins, and components of the mitochondrial protein import machinery. Among mitochondrial proteins that are downregulated in response to PE21 are components of the mitochondrial division (fission) apparatus as well as proteins implicated in mitochondrial RNA synthesis, processing and translation. All mitochondrial processes that are upregulated or downregulated by PE21 play essential roles in the PE21-dependent delay of yeast chronological aging because they allow to amend the pattern of age-related changes in mitochondrial respiration, mitochondrial membrane potential and mitochondrial ROS production [55].

The challenge for the future is to define mechanisms underlying the PE21-dependent remodeling of the ER and mitochondrial membrane lipidomes in chronologically aging yeast. Because PE21 slows aging by inhibiting a form of the pro-aging protein kinase Sch9 that is activated by the pro-aging PKH1/2 signaling pathway [56], one could envision that these mechanisms may involve certain changes in the reversible phosphorylation of proteins implicated in lipid metabolism and transport in the ER and mitochondria. Our ongoing studies address the validity of this assumption.

## MATERIALS AND METHODS

### Yeast strains, media and growth conditions

The wild-type strain *Saccharomyces cerevisiae* BY4742 (*MAT*α* his3Δ1 leu2Δ0 lys2Δ0 ura3Δ0*) and single-gene-deletion mutant strains in the BY4742 genetic background (all from Thermo Scientific/Open Biosystems) were grown in a synthetic minimal YNB medium (0.67% (w/v) Yeast Nitrogen Base without amino acids from Fisher Scientific; #DF0919-15-3) initially containing 2% (w/v) glucose (#D16-10; Fisher Scientific), 20 mg/l *L*-histidine (# H8125; Sigma), 30 mg/l *L*-leucine (#L8912; Sigma), 30 mg/l *L*-lysine (#L5501; Sigma) and 20 mg/l uracil (#U0750; Sigma), with 0.1% (w/v) PE21 (Idunn Technologies Inc.) or without it. PE21 is an ethanol/water extract from the bark of *Salix alba* [55]. If added to growth medium at the time of cell inoculation at a final concentration of 0.1% (w/v), PE21 increases both the mean and maximum chronological lifespans of wild-type strain cultured in medium initially containing 2% (w/v) glucose [55]. A 20% stock solution of PE21 in ethanol was made on the day of adding this PE to cell cultures. The stock solution of PE21 was added to growth medium with 2% (w/v) glucose immediately following cell inoculation into the medium. In a culture supplemented with PE21, ethanol was used as a vehicle at the final concentration of 0.5% (v/v). In the same experiment, yeast cells were also subjected to ethanol-mock treatment by being cultured in growth medium initially containing 2% (w/v) glucose and 0.5% (v/v) ethanol. Cells were cultured at 30° C with rotational shaking at 200 rpm in Erlenmeyer flasks at a “flask volume/medium volume” ratio of 5:1.

### Mass spectrometric identification and quantitation of cellular lipids

Extraction of cellular lipids and their mass spectrometric identification and quantitation were performed as previously described [7]. Briefly, a sample of cells was taken from a culture on a certain day of culturing. A fraction of the sample was diluted to determine the total number of cells using a hemocytometer (# 3200; Hausser Scientific). 5 × 10^7^ cells were harvested by centrifugation in a Centra CL2 clinical centrifuge for 5 min at 3,000 × g at room temperature. The cell pellet was washed once in ice-cold nanopure water and once in ice-cold 155 mM ammonium bicarbonate (pH 8.0), and the cells were harvested by centrifugation at 16,000 × g for 1 min at 4° C. The cell pellet was stored at –80° C until lipid extraction. For lipid extraction, the pelleted cells kept at –80° C were thawed on ice before being resuspended in 200 μl of ice-cold nanopure water. The re-suspended sample was transferred to a 15-ml high-strength glass screw top centrifuge tube with a Teflon lined cap (#0556912; Fisher Scientific). The volume of each sample was topped off to 1 ml with ice-cold nanopure water. To each tube the following was added: 20 μl of the internal standard mix prepared in Chromasolv HPLC (>99.9%) chloroform (Sigma-Aldrich) as described [7], 800 μl of 425–600 μM acid-washed glass beads to break open the cells (#G8772; Sigma-Aldrich) and 3 ml of a Chromasolv HPLC (>99.9%) chloroform-methanol mixture (both from Sigma-Aldrich) at a 17:1 ratio. The samples were then vortexed vigorously for 2 h at 4° C and subjected to centrifugation in a Centra CL2 clinical centrifuge at 3,000 × g for 5 min at room temperature. The lower organic phase was then transferred to another 15-ml high-strength glass screw top centrifuge tube using a glass Pasteur pipette with careful attention not to disrupt the glass beads or upper aqueous phase. 1.5 ml of chloroform-methanol (2:1) solution was added to the remaining upper aqueous phase. The samples were again vortexed vigorously at 4° C for 2 h. The initially separated organic phase was kept at 4° C for the duration of the second vortexing. At the end of 2-h vortexing, the samples were again centrifuged for 5 min at 3,000 × g at room temperature; the lower organic phase was then separated and added to the corresponding initial organic phase with a glass Pasteur pipette. With both lower organic phases combined, the solvent was evaporated off by nitrogen gas flow. Once all solvent was evaporated, the remaining lipid film was dissolved in 100 μl of chloroform-methanol (1:2) and immediately transferred into 2-ml glass vials with Teflon screw tops to avoid evaporation until samples were analyzed by mass spectrometry (MS). Samples were then stored at –80° C and ran on the LTQ Orbitrap Mass Spectrometer within one week of the extraction. Samples were diluted (1:1) with chloroform/methanol (1:2) mixture supplemented with 0.1% ammonium hydroxide. Lipids were resolved by direct injection using a Thermo Orbitrap Velos mass spectrometer equipped with a HESI-II ion source (Thermo Scientific, Waltham, MA, USA) at a flow rate of 5 ml/min. The optimized tune setting and instrument methods for mass spectrometric analysis of lipids were previously described [7]. Mass spectra were converted to open format mzXML using the ProteoWizard MSConvert software (http://proteowizard.sourceforge.net/), the file format used by the Lipid Identification Software LipidXplorer (https://wiki.mpi-cbg.de/lipidx/Main_Page) for the automated detection and quantitation of lipid species. Data were normalized by taking the ratio of signal intensity of precursor ions to that of their respective lipid class-specific internal standard (spiked standard), multiplied by the concentration of that standard to give a molar quantity.

### Total cell lysates preparation

Total cell lysates were made by vortexing the cells in TCL buffer (25 mM Tris/HCl pH 8.5, 150 mM NaCl, 1 mM EDTA, 0.1 mM dithiothreitol [DTT], 4% CHAPS, 1 mM PMSF, protease inhibitor cocktail [#P8340; Sigma]) with glass beads three times for 1 min. Lysates were then centrifuged for 3 min at 21,000 × g at 4° C and supernatants collected.

### Mass spectrometric identification and quantitation of cellular proteins

SDS-PAGE of cellular proteins was performed as previously described [255]. Protein bands stained with QC Colloidal Coomassie Blue were cut out from an SDS-PAGE gel with a razor blade. The gel pieces were placed in individual 0.5-ml siliconized Eppendorf tubes. The bands were destained by washing twice with distilled water for 1 h. The bands were then incubated in 50 μl of acetonitrile (ACN) for 5 min at 37° C, after which ACN was removed and the bands were dried at 37° C. Next, the destained bands were incubated in 50 μl of 10 mM DTT for 30 min at 37° C to reduce thiol groups in peptides. DTT was discarded and the bands were incubated in 50 μl of 55 mM iodoacetamide (IAA) for 20 min at 37° C in the dark to remove the residual DTT. IAA was removed, and the bands were incubated in 50 μl of a 1:1 mixture of 100 mM ammonium bicarbonate (ABC) and 50% acetonitrile for 10 min at 37° C. The mixture was discarded, and the bands were incubated twice in 50 μl of ACN under the same conditions and dried at 37° C. The trypsin and trypsin buffer were prepared as follows: 1) 1.6 ml of a 1:1 mixture of 100 mM ABC and 10mM CaCl2 were used to resuspend 20 μg of trypsin; and 2) for protein digest, 50 μl of trypsin solution (1 mg/ml) was added to the bands, which were then incubated overnight at 37° C. The following day, the samples were spun down and the supernatants containing peptides were transferred to new 0.5-ml siliconized Eppendorf tubes. To extract more peptides, the gel pieces were subjected to several washes and treatments at room temperature; the supernatants were conserved and combined with the first set to extracted peptides. For the first extraction, the bands were initially incubated in 50 μl of 25 mM ABC for 10 min and then in 50 μl of ACN for 10 min. The samples were spun down, and the supernatant were added to the first set of extracted peptides. For the second extraction, the bands were incubated in 50 μl of 5% formic acid for 10 min and then in 50 μl of ACN for 10 min. The samples were spun down, and the supernatant were combined with the first set of extracted peptides. The gel pieces were no longer used and discarded. To prevent possible oxidation during storage, 12.5 ml of 100 mM DTT was added to each set of peptides. The peptides were completely dried in a Speed-Vac at medium temperature settings (37° C) for 2 h and stored at –20° C until MS analysis. Dried peptides were resuspended in 20 μl of 5% ACN. For each recovered protein band, an aliquot of 10 μl of dried peptides in 5% ACN was diluted 2-fold in nanopure water for MS analysis. Samples can be stored at –20° C until being subjected to MS analysis. Individual proteins composing each band were then identified by reverse phase high performance liquid chromatography coupled to mass spectrometry (RP-HPLC/MS) using an LTQ Orbitrap. 3-μl aliquots of peptides were separated in ACN gradient using a 100-μM capillary column packed with C18 mobile phase. Once acquiring time was completed using the LTQ Orbitrap, the raw mass spectrometry data file obtained by Xcalibur were analyzed using the Thermo Scientific Xcalibur Proteome Discoverer application (version 1.3) hereafter referred to as the Proteome Discoverer. The Proteome Discoverer was used to identify individual proteins by comparing the raw data of mass spectra of digested fragments to the mass spectra of peptides within the Uniprot FASTA database. The analysis by the Proteome Discoverer coupled to the FASTA database was enabled by using the peak-finding search engine SEQUEST. The SEQUEST engine processes MS data using a peak-finding algorithm to search the raw data for generating a peak probability list with relative protein abundances. The “Proteome Discoverer” software was used to calculate the exponentially modified protein abundance index (emPAI), a measure of the relative abundance of cellular proteins in a pair of analyzed datasets.

### Chronological lifespan assay

A sample of cells was taken from a culture at a certain day following cell inoculation and PE21 addition into the medium. A fraction of the sample was diluted to determine the total number of cells using a hemacytometer. Another fraction of the cell sample was diluted, and serial dilutions of cells were plated in duplicate onto YEP medium (1% (w/v) yeast extract, 2% (w/v) peptone; both from Fisher Scientific; #BP1422-2 and #BP1420-2, respectively) containing 2% glucose (#D16-10; Fisher Scientific) as carbon source. After 2 d of incubation at 30° C, the number of colony forming units (CFU) per plate was counted. The number of CFU was defined as the number of viable cells in a sample. For each culture, the percentage of viable cells was calculated as follows: (number of viable cells per ml/total number of cells per ml) × 100. The percentage of viable cells in mid-logarithmic growth phase was set at 100%.

### Cell viability assay for monitoring the susceptibility of yeast to a mode of cell death induced by palmitoleic acid (POA)

A sample of cells was taken from a culture on a certain day of culturing. A fraction of the sample was diluted to determine the total number of cells using a hemocytometer. 8 × 10^7^ cells were harvested by centrifugation for 1 min at 21,000 × g at room temperature and resuspended in 8 ml of YP medium containing 0.2% glucose as carbon source. Each cell suspension was divided into 8 equal aliquots. Three pairs of aliquots were supplemented with POA (#P9417; Sigma) from a 50-mM stock solution (in 10% chloroform, 45% hexane and 45% ethanol; #650498, #248878 and #34852, respectively; all from Sigma). The final concentration of POA was 0.05 mM, 0.1 mM or 0.15 mM for each pair of aliquots; in all these aliquots, the final concentrations of chloroform, hexane and ethanol were 0.03%, 0.135% and 0.135%, respectively. One pair of aliquots was supplemented only with chloroform, hexane and ethanol added to the final concentrations of 0.03%, 0.135% and 0.135%, respectively. All aliquots were then incubated for 2 h at 30° C on a Labquake rotator (#400110; Thermolyne/Barnstead International) set for 360° rotation. Serial dilutions of cells were plated in duplicate onto plates containing YP medium with 2% glucose as carbon source. After 2 d of incubation at 30° C, the number of colony forming units (CFU) per plate was counted. The number of CFU was defined as the number of viable cells in a sample. For each aliquot of cells exposed to POA, the % of viable cells was calculated as follows: (number of viable cells per ml in the aliquot exposed to POA/number of viable cells per ml in the control aliquot that was not exposed to POA) × 100.

### Fluorescence microscopy

Propidium iodide (PI; #P4170, Sigma) staining for visualizing the extent of plasma membrane permeability for small molecules [256] and Annexin V (#A13201; Thermo Fisher Scientific) staining for visualizing externalized phosphatidylserine [256] were performed according to established procedures. Live imaging was performed on a Leica DM6000B epifluorescence microscope equipped with a high-resolution Hamamatsu Orca ER CCD camera using oil immersion and a 100× objective. Images were acquired with 20-ms exposures using PerkinElmer Volocity software. Image files were exported as TIFFs then opened in ImageJ, where the percentage of PI- and Annexin V-positive cells was calculated.

### Statistical analysis

Statistical analysis was performed using Microsoft Excel’s (2010) Analysis ToolPack-VBA. All data on cell survival are presented as mean ± SEM. The *p* values for comparing the means of two groups using an unpaired two-tailed *t* test were calculated with the help of the GraphPad Prism 7 statistics software. The logrank test for comparing each pair of survival curves was performed with GraphPad Prism 7. Two survival curves were considered statistically different if the *p* value was less than 0.05.

## SUPPLEMENTARY MATERIALS



## Abbreviations

Ac-CoA: acetyl-CoA
ACN: acetonitrile
ADHAP: acyl-dihydroxyacetone phosphate
AMPK: AMP-dependent protein kinase
ATG: autophagy
CDP: cytidine diphosphate
CFU: colony forming units
CL: cardiolipin
CLS: chronological lifespan
CR: caloric restriction
DAG: diacylglycerols
DTT: dithiothreitol
emPAI: exponentially modified protein abundance index
ER: endoplasmic reticulum
ETC: electron transport chain
FA-CoA: fatty acyl-CoA esters
FFA: free (unesterified) fatty acids
IAA: iodoacetamide
IGF-1: insulin/insulin-like growth factor 1
IMM: inner mitochondrial membrane
IMS: intermediate space
L: logarithmic growth phase
LD: lipid droplets
LPA: lysophosphatidic acid
MAG: monoacylglycerols
MS: mass spectrometry
mTORC1: mammalian target of rapamycin complex 1
OMM: outer mitochondrial membrane
OXPHOS: oxidative phosphorylation
PA: phosphatidic acid
PC: phosphatidylcholine
PCA: principal component analysis
PD: post-diauxic growth phase
PE: phosphatidylethanolamine
PG: phosphatidylglycerol
PI: phosphatidylinositol
PKA: protein kinase A
PKH1/2: Pkb-activating kinase homolog
PM: plasma membrane
POA: palmitoleic acid
PS: phosphatidylserine
RCD: regulated cell death
ROS: reactive oxygen species
RP-HPLC/MS: reverse phase high performance liquid chromatography coupled to mass spectrometry
SNF: sucrose non-fermenting
ST: stationary growth phase
TAG: triacylglycerols
TCA: tricarboxylic acid
UPR^ER^: unfolded protein response in the endoplasmic reticulum
WT: wild type
YNB: yeast nitrogen base

## References

[1] Fontana L, Partridge L, Longo VD Extending healthy life span-from yeast to humans. Science. 2010; 328:321–26. 10.1126/science.1172539. 20395504 PMC3607354

[2] Kaeberlein M Lessons on longevity from budding yeast. Nature. 2010; 464:513–19. 10.1038/nature08981. Erratum in: Nature. 2010 Apr 29;464(7293):1390. 10.1038/nature08981. 20336133 PMC3696189

[3] Weissman J, Guthrie C, Fink GR Guide to Yeast Genetics: Functional Genomics, Proteomics, and Other Systems Analyses Burlington: Academic Press; 2010 Available from: https://www.elsevier.com/books/guide-to-yeast-genetics-functional-genomics-proteomics-and-other-systems-analysis/guthrie/978-0-12-375171-3.

[4] Botstein D, Fink GR Yeast: an experimental organism for 21st Century biology. Genetics. 2011; 189:695–704. 10.1534/genetics.111.130765. 22084421 PMC3213361

[5] Longo VD, Shadel GS, Kaeberlein M, Kennedy B Replicative and chronological aging in Saccharomyces cerevisiae. Cell Metab. 2012; 16:18–31. 10.1016/j.cmet.2012.06.002. 22768836 PMC3392685

[6] Arlia-Ciommo A, Leonov A, Piano A, Svistkova V, Titorenko VI Cell-autonomous mechanisms of chronological aging in the yeast Saccharomyces cerevisiae. Microb Cell. 2014; 1:163–78. 10.15698/mic2014.06.152. 28357241 PMC5354559

[7] Richard VR, Bourque SD, Titorenko VI Metabolomic and lipidomic analyses of chronologically aging yeast. Methods Mol Biol. 2014; 1205:359–73. 10.1007/978-1-4939-1363-3_21. 25213255

[8] Pitt JN, Kaeberlein M Why Is Aging Conserved and What Can We Do about it? PLoS Biol. 2015; 13:e1002131. 10.1371/journal.pbio.1002131. . Erratum in: Correction: Why Is Aging Conserved and What Can We Do about it? [PLoS Biol. 2015]. 10.1371/journal.pbio.1002131. 25923592 PMC4414409

[9] Crane MM, Kaeberlein M The paths of mortality: how understanding the biology of aging can help explain systems behavior of single cells. Curr Opin Syst Biol. 2018; 8:25–31. 10.1016/j.coisb.2017.11.010. 29552673 PMC5851462

[10] Mair W, Dillin A Aging and survival: the genetics of life span extension by dietary restriction. Annu Rev Biochem. 2008; 77:727–54. 10.1146/annurev.biochem.77.061206.171059. 18373439

[11] Eisenberg T, Knauer H, Schauer A, Büttner S, Ruckenstuhl C, Carmona-Gutierrez D, Ring J, Schroeder S, Magnes C, Antonacci L, Fussi H, Deszcz L, Hartl R, et al Induction of autophagy by spermidine promotes longevity. Nat Cell Biol. 2009; 11:1305–14. 10.1038/ncb1975. 19801973

[12] Madeo F, Pietrocola F, Eisenberg T, Kroemer G Caloric restriction mimetics: towards a molecular definition. Nat Rev Drug Discov. 2014; 13:727–40. 10.1038/nrd4391. 25212602

[13] Leonov A, Arlia-Ciommo A, Piano A, Svistkova V, Lutchman V, Medkour Y, Titorenko VI Longevity extension by phytochemicals. Molecules. 2015; 20:6544–72. 10.3390/molecules20046544. 25871373 PMC6272139

[14] Moskalev A, Chernyagina E, de Magalhães JP, Barardo D, Thoppil H, Shaposhnikov M, Budovsky A, Fraifeld VE, Garazha A, Tsvetkov V, Bronovitsky E, Bogomolov V, Scerbacov A, et al Geroprotectors.org: a new, structured and curated database of current therapeutic interventions in aging and age-related disease. Aging (Albany NY). 2015; 7:616–28. 10.18632/aging.100799. 26342919 PMC4600621

[15] Moskalev A, Chernyagina E, Tsvetkov V, Fedintsev A, Shaposhnikov M, Krut’ko V, Zhavoronkov A, Kennedy BK Developing criteria for evaluation of geroprotectors as a key stage toward translation to the clinic. Aging Cell. 2016; 15:407–15. 10.1111/acel.12463. 26970234 PMC4854916

[16] Moskalev A, Chernyagina E, Kudryavtseva A, Shaposhnikov M Geroprotectors: A unified concept and screening approaches. Aging Dis. 2017; 8:354–63. 10.14336/AD.2016.1022. 28580190 PMC5440114

[17] Madeo F, Carmona-Gutierrez D, Kepp O, Kroemer G Spermidine delays aging in humans. Aging (Albany NY). 2018; 10:2209–11. 10.18632/aging.101517. 30082504 PMC6128428

[18] Madeo F, Eisenberg T, Pietrocola F, Kroemer G Spermidine in health and disease. Science. 2018; 359. 10.1126/science.aan2788. 29371440

[19] López-Otín C, Blasco MA, Partridge L, Serrano M, Kroemer G The hallmarks of aging. Cell. 2013; 153:1194–217. 10.1016/j.cell.2013.05.039. 23746838 PMC3836174

[20] Olshansky SJ, Martin GM, Kirkland JL Aging: The Longevity Dividend Cold Spring Harbor: Cold Spring Harbor Laboratory Press; 2016. .

[21] Greer EL, Brunet A Signaling networks in aging. J Cell Sci. 2008; 121:407–12. 10.1242/jcs.021519. 18256383

[22] Goldberg AA, Bourque SD, Kyryakov P, Boukh-Viner T, Gregg C, Beach A, Burstein MT, Machkalyan G, Richard V, Rampersad S, Titorenko VI A novel function of lipid droplets in regulating longevity. Biochem Soc Trans. 2009; 37:1050–55. 10.1042/BST0371050. 19754450

[23] Kenyon CJ The genetics of ageing. Nature. 2010; 464:504–12. 10.1038/nature08980. Erratum in: Nature. 2010 Sep 30;467(7315):622. 10.1038/nature08980. 20336132

[24] Kyryakov P, Beach A, Richard VR, Burstein MT, Leonov A, Levy S, Titorenko VI Caloric restriction extends yeast chronological lifespan by altering a pattern of age-related changes in trehalose concentration. Front Physiol. 2012; 3:256. 10.3389/fphys.2012.00256. 22783207 PMC3390693

[25] Beach A, Leonov A, Arlia-Ciommo A, Svistkova V, Lutchman V, Titorenko VI Mechanisms by which different functional states of mitochondria define yeast longevity. Int J Mol Sci. 2015; 16:5528–54. 10.3390/ijms16035528. 25768339 PMC4394491

[26] Goodell MA, Rando TA Stem cells and healthy aging. Science. 2015; 350:1199–204. 10.1126/science.aab3388. 26785478

[27] Medkour Y, Svistkova V, Titorenko VI Cell-Nonautonomous Mechanisms Underlying Cellular and Organismal Aging. Int Rev Cell Mol Biol. 2016; 321:259–97. 10.1016/bs.ircmb.2015.09.003. 26811290

[28] Steffen KK, Dillin A A Ribosomal Perspective on Proteostasis and Aging. Cell Metab. 2016; 23:1004–12. 10.1016/j.cmet.2016.05.013. 27304502

[29] Leonov A, Feldman R, Piano A, Arlia-Ciommo A, Lutchman V, Ahmadi M, Elsaser S, Fakim H, Heshmati-Moghaddam M, Hussain A, Orfali S, Rajen H, Roofigari-Esfahani N, et al Caloric restriction extends yeast chronological lifespan via a mechanism linking cellular aging to cell cycle regulation, maintenance of a quiescent state, entry into a non-quiescent state and survival in the non-quiescent state. Oncotarget. 2017; 8:69328–50. 10.18632/oncotarget.20614. 29050207 PMC5642482

[30] Anisimova AS, Alexandrov AI, Makarova NE, Gladyshev VN, Dmitriev SE Protein synthesis and quality control in aging. Aging (Albany NY). 2018; 10:4269–88. 10.18632/aging.101721. 30562164 PMC6326689

[31] Hansen M, Rubinsztein DC, Walker DW Autophagy as a promoter of longevity: insights from model organisms. Nat Rev Mol Cell Biol. 2018; 19:579–93. 10.1038/s41580-018-0033-y. . Erratum in: Publisher Correction: Autophagy as a promoter of longevity: insights from model organisms. [Nat Rev Mol Cell Biol. 2018]. 10.1038/s41580-018-0033-y. 30006559 PMC6424591

[32] Mitrofanova D, Dakik P, McAuley M, Medkour Y, Mohammad K, Titorenko VI Lipid metabolism and transport define longevity of the yeast Saccharomyces cerevisiae. Front Biosci (Landmark Ed). 2018; 23:1166–94. 10.2741/4638. 28930594

[33] So WK, Cheung TH Molecular Regulation of Cellular Quiescence: A Perspective from Adult Stem Cells and Its Niches. Methods Mol Biol. 2018; 1686:1–25. 10.1007/978-1-4939-7371-2_1. 29030809

[34] Dakik P, Medkour Y, Mohammad K, Titorenko VI Mechanisms through which some mitochondria-generated metabolites act as second messengers that are essential contributors to the aging process in eukaryotes across phyla. Front Physiol. 2019; 10:461. 10.3389/fphys.2019.00461. 31057428 PMC6482166

[35] Escobar KA, Cole NH, Mermier CM, VanDusseldorp TA Autophagy and aging: maintaining the proteome through exercise and caloric restriction. Aging Cell. 2019; 18:e12876. 10.1111/acel.12876. 30430746 PMC6351830

[36] Riera CE, Merkwirth C, De Magalhaes Filho CD, Dillin A Signaling Networks Determining Life Span. Annu Rev Biochem. 2016; 85:35–64. 10.1146/annurev-biochem-060815-014451. 27294438

[37] Salminen A, Kaarniranta K, Kauppinen A Age-related changes in AMPK activation: role for AMPK phosphatases and inhibitory phosphorylation by upstream signaling pathways. Ageing Res Rev. 2016; 28:15–26. 10.1016/j.arr.2016.04.003. 27060201

[38] Pan H, Finkel T Key proteins and pathways that regulate lifespan. J Biol Chem. 2017; 292:6452–60. 10.1074/jbc.R116.771915. 28264931 PMC5399099

[39] Slack C, Tullet J Signal Transduction Pathways in Ageing. Subcell Biochem. 2018; 90:323–50. 10.1007/978-981-13-2835-0_11. 30779014

[40] Powers RW 3rd, Kaeberlein M, Caldwell SD, Kennedy BK, Fields S Extension of chronological life span in yeast by decreased TOR pathway signaling. Genes Dev. 2006; 20:174–84. 10.1101/gad.1381406. 16418483 PMC1356109

[41] Urban J, Soulard A, Huber A, Lippman S, Mukhopadhyay D, Deloche O, Wanke V, Anrather D, Ammerer G, Riezman H, Broach JR, De Virgilio C, Hall MN, Loewith R Sch9 is a major target of TORC1 in Saccharomyces cerevisiae. Mol Cell. 2007; 26:663–74. 10.1016/j.molcel.2007.04.020. 17560372

[42] Wei M, Fabrizio P, Hu J, Ge H, Cheng C, Li L, Longo VD Life span extension by calorie restriction depends on Rim15 and transcription factors downstream of Ras/PKA, Tor, and Sch9. PLoS Genet. 2008; 4:e13. 10.1371/journal.pgen.0040013. 18225956 PMC2213705

[43] Alvers AL, Fishwick LK, Wood MS, Hu D, Chung HS, Dunn WA Jr, Aris JP Autophagy and amino acid homeostasis are required for chronological longevity in Saccharomyces cerevisiae. Aging Cell. 2009; 8:353–69. 10.1111/j.1474-9726.2009.00469.x. 19302372 PMC2802268

[44] Morselli E, Galluzzi L, Kepp O, Criollo A, Maiuri MC, Tavernarakis N, Madeo F, Kroemer G Autophagy mediates pharmacological lifespan extension by spermidine and resveratrol. Aging (Albany NY). 2009; 1:961–70. 10.18632/aging.100110. 20157579 PMC2815753

[45] Lu JY, Lin YY, Sheu JC, Wu JT, Lee FJ, Chen Y, Lin MI, Chiang FT, Tai TY, Berger SL, Zhao Y, Tsai KS, Zhu H, et al Acetylation of yeast AMPK controls intrinsic aging independently of caloric restriction. Cell. 2011; 146:969–79. 10.1016/j.cell.2011.07.044. 21906795 PMC3176974

[46] Richard VR, Leonov A, Beach A, Burstein MT, Koupaki O, Gomez-Perez A, Levy S, Pluska L, Mattie S, Rafesh R, Iouk T, Sheibani S, Greenwood M, et al Macromitophagy is a longevity assurance process that in chronologically aging yeast limited in calorie supply sustains functional mitochondria and maintains cellular lipid homeostasis. Aging (Albany NY). 2013; 5:234–69. 10.18632/aging.100547. 23553280 PMC3651518

[47] Conrad M, Schothorst J, Kankipati HN, Van Zeebroeck G, Rubio-Texeira M, Thevelein JM Nutrient sensing and signaling in the yeast Saccharomyces cerevisiae. FEMS Microbiol Rev. 2014; 38:254–99. 10.1111/1574-6976.12065. 24483210 PMC4238866

[48] Huang X, Withers BR, Dickson RC Sphingolipids and lifespan regulation. Biochim Biophys Acta. 2014; 1841:657–64. 10.1016/j.bbalip.2013.08.006. 23954556 PMC3925463

[49] Swinnen E, Ghillebert R, Wilms T, Winderickx J Molecular mechanisms linking the evolutionary conserved TORC1-Sch9 nutrient signalling branch to lifespan regulation in Saccharomyces cerevisiae. FEMS Yeast Res. 2014; 14:17–32. 10.1111/1567-1364.12097. 24102693

[50] Jiao R, Postnikoff S, Harkness TA, Arnason TG The SNF1 kinase ubiquitin-associated domain restrains its activation, activity, and the yeast life span. J Biol Chem. 2015; 290:15393–404. 10.1074/jbc.M115.647032. 25869125 PMC4505455

[51] Teixeira V, Costa V Unraveling the role of the Target of Rapamycin signaling in sphingolipid metabolism. Prog Lipid Res. 2016; 61:109–33. 10.1016/j.plipres.2015.11.001. 26703187

[52] Goldberg AA, Richard VR, Kyryakov P, Bourque SD, Beach A, Burstein MT, Glebov A, Koupaki O, Boukh-Viner T, Gregg C, Juneau M, English AM, Thomas DY, Titorenko VI Chemical genetic screen identifies lithocholic acid as an anti-aging compound that extends yeast chronological life span in a TOR-independent manner, by modulating housekeeping longevity assurance processes. Aging (Albany NY). 2010; 2:393–414. 10.18632/aging.100168. 20622262 PMC2933888

[53] Minois N, Carmona-Gutierrez D, Madeo F Polyamines in aging and disease. Aging (Albany NY). 2011; 3:716–32. 10.18632/aging.100361. 21869457 PMC3184975

[54] Arlia-Ciommo A, Piano A, Svistkova V, Mohtashami S, Titorenko VI Mechanisms underlying the anti-aging and anti-tumor effects of lithocholic bile acid. Int J Mol Sci. 2014; 15:16522–43. 10.3390/ijms150916522. 25238416 PMC4200844

[55] Lutchman V, Medkour Y, Samson E, Arlia-Ciommo A, Dakik P, Cortes B, Feldman R, Mohtashami S, McAuley M, Chancharoen M, Rukundo B, Simard É, Titorenko VI Discovery of plant extracts that greatly delay yeast chronological aging and have different effects on longevity-defining cellular processes. Oncotarget. 2016; 7:16542–66. 10.18632/oncotarget.7665. 26918729 PMC4941334

[56] Lutchman V, Dakik P, McAuley M, Cortes B, Ferraye G, Gontmacher L, Graziano D, Moukhariq FZ, Simard É, Titorenko VI Six plant extracts delay yeast chronological aging through different signaling pathways. Oncotarget. 2016; 7:50845–63. 10.18632/oncotarget.10689. 27447556 PMC5239441

[57] Ingram DK, Zhu M, Mamczarz J, Zou S, Lane MA, Roth GS, deCabo R Calorie restriction mimetics: an emerging research field. Aging Cell. 2006; 5:97–108. 10.1111/j.1474-9726.2006.00202.x. 16626389

[58] Ingram DK, Roth GS Calorie restriction mimetics: can you have your cake and eat it, too? Ageing Res Rev. 2015; 20:46–62. 10.1016/j.arr.2014.11.005. 25530568

[59] Longo VD, Antebi A, Bartke A, Barzilai N, Brown-Borg HM, Caruso C, Curiel TJ, de Cabo R, Franceschi C, Gems D, Ingram DK, Johnson TE, Kennedy BK, et al Interventions to Slow Aging in Humans: Are We Ready? Aging Cell. 2015; 14:497–510. 10.1111/acel.12338. 25902704 PMC4531065

[60] Giorgio M, Trinei M, Migliaccio E, Pelicci PG Hydrogen peroxide: a metabolic by-product or a common mediator of ageing signals? Nat Rev Mol Cell Biol. 2007; 8:722–28. 10.1038/nrm2240. 17700625

[61] Ristow M, Schmeisser K Mitohormesis: Promoting Health and Lifespan by Increased Levels of Reactive Oxygen Species (ROS). Dose Response. 2014; 12:288–341. 10.2203/dose-response.13-035.Ristow. 24910588 PMC4036400

[62] Blüher M, Kahn BB, Kahn CR Extended longevity in mice lacking the insulin receptor in adipose tissue. Science. 2003; 299:572–74. 10.1126/science.1078223. 12543978

[63] Chiu CH, Lin WD, Huang SY, Lee YH Effect of a C/EBP gene replacement on mitochondrial biogenesis in fat cells. Genes Dev. 2004; 18:1970–75. 10.1101/gad.1213104. 15289464 PMC514177

[64] Picard F, Kurtev M, Chung N, Topark-Ngarm A, Senawong T, Machado De Oliveira R, Leid M, McBurney MW, Guarente L Sirt1 promotes fat mobilization in white adipocytes by repressing PPAR-gamma. Nature. 2004; 429:771–76. 10.1038/nature02583. Erratum in: Nature. 2004 Aug 19;430(7002):921. 10.1038/nature02583. 15175761 PMC2820247

[65] Bordone L, Guarente L Calorie restriction, SIRT1 and metabolism: understanding longevity. Nat Rev Mol Cell Biol. 2005; 6:298–305. 10.1038/nrm1616. 15768047

[66] Grönke S, Mildner A, Fellert S, Tennagels N, Petry S, Müller G, Jäckle H, Kühnlein RP Brummer lipase is an evolutionary conserved fat storage regulator in Drosophila. Cell Metab. 2005; 1:323–30. 10.1016/j.cmet.2005.04.003. 16054079

[67] Skorupa DA, Dervisefendic A, Zwiener J, Pletcher SD Dietary composition specifies consumption, obesity, and lifespan in Drosophila melanogaster. Aging Cell. 2008; 7:478–90. 10.1111/j.1474-9726.2008.00400.x. 18485125 PMC2574586

[68] Goldberg AA, Bourque SD, Kyryakov P, Gregg C, Boukh-Viner T, Beach A, Burstein MT, Machkalyan G, Richard V, Rampersad S, Cyr D, Milijevic S, Titorenko VI Effect of calorie restriction on the metabolic history of chronologically aging yeast. Exp Gerontol. 2009; 44:555–71. 10.1016/j.exger.2009.06.001. 19539741

[69] Argmann C, Dobrin R, Heikkinen S, Auburtin A, Pouilly L, Cock TA, Koutnikova H, Zhu J, Schadt EE, Auwerx J Ppargamma2 is a key driver of longevity in the mouse. PLoS Genet. 2009; 5:e1000752. 10.1371/journal.pgen.1000752. 19997628 PMC2780700

[70] Narbonne P, Roy R Caenorhabditis elegans dauers need LKB1/AMPK to ration lipid reserves and ensure long-term survival. Nature. 2009; 457:210–14. 10.1038/nature07536. 19052547

[71] Soukas AA, Kane EA, Carr CE, Melo JA, Ruvkun G Rictor/TORC2 regulates fat metabolism, feeding, growth, and life span in Caenorhabditis elegans. Genes Dev. 2009; 23:496–511. 10.1101/gad.1775409. 19240135 PMC2648650

[72] Bjedov I, Toivonen JM, Kerr F, Slack C, Jacobson J, Foley A, Partridge L Mechanisms of life span extension by rapamycin in the fruit fly Drosophila melanogaster. Cell Metab. 2010; 11:35–46. 10.1016/j.cmet.2009.11.010. 20074526 PMC2824086

[73] Shmookler Reis RJ, Xu L, Lee H, Chae M, Thaden JJ, Bharill P, Tazearslan C, Siegel E, Alla R, Zimniak P, Ayyadevara S Modulation of lipid biosynthesis contributes to stress resistance and longevity of C. elegans mutants. Aging (Albany NY). 2011; 3:125–47. 10.18632/aging.100275. 21386131 PMC3082008

[74] Hou NS, Taubert S Function and Regulation of Lipid Biology in Caenorhabditis elegans Aging. Front Physiol. 2012; 3:143. 10.3389/fphys.2012.00143. 22629250 PMC3355469

[75] Katewa SD, Demontis F, Kolipinski M, Hubbard A, Gill MS, Perrimon N, Melov S, Kapahi P Intramyocellular fatty-acid metabolism plays a critical role in mediating responses to dietary restriction in Drosophila melanogaster. Cell Metab. 2012; 16:97–103. 10.1016/j.cmet.2012.06.005. 22768842 PMC3400463

[76] Streeper RS, Grueter CA, Salomonis N, Cases S, Levin MC, Koliwad SK, Zhou P, Hirschey MD, Verdin E, Farese RV Jr Deficiency of the lipid synthesis enzyme, DGAT1, extends longevity in mice. Aging (Albany NY). 2012; 4:13–27. 10.18632/aging.100424. 22291164 PMC3292902

[77] Beach A, Richard VR, Leonov A, Burstein MT, Bourque SD, Koupaki O, Juneau M, Feldman R, Iouk T, Titorenko VI Mitochondrial membrane lipidome defines yeast longevity. Aging (Albany NY). 2013; 5:551–74. 10.18632/aging.100578. 23924582 PMC3765583

[78] Gonzalez-Covarrubias V Lipidomics in longevity and healthy aging. Biogerontology. 2013; 14:663–72. 10.1007/s10522-013-9450-7. 23948799

[79] Gonzalez-Covarrubias V, Beekman M, Uh HW, Dane A, Troost J, Paliukhovich I, van der Kloet FM, Houwing-Duistermaat J, Vreeken RJ, Hankemeier T, Slagboom EP Lipidomics of familial longevity. Aging Cell. 2013; 12:426–34. 10.1111/acel.12064. 23451766 PMC3709127

[80] Hansen M, Flatt T, Aguilaniu H Reproduction, fat metabolism, and life span: what is the connection? Cell Metab. 2013; 17:10–19. 10.1016/j.cmet.2012.12.003. Erratum in: Cell Metab. 2014 Jun 3;19(6):1066. 10.1016/j.cmet.2012.12.003. 23312280 PMC3567776

[81] Jové M, Naudí A, Aledo JC, Cabré R, Ayala V, Portero-Otin M, Barja G, Pamplona R Plasma long-chain free fatty acids predict mammalian longevity. Sci Rep. 2013; 3:3346. 10.1038/srep03346. 24284984 PMC3842621

[82] Karpac J, Biteau B, Jasper H Misregulation of an adaptive metabolic response contributes to the age-related disruption of lipid homeostasis in Drosophila. Cell Rep. 2013; 4:1250–61. 10.1016/j.celrep.2013.08.004. 24035390 PMC3832190

[83] Kniazeva M, Han M Fat chance for longevity. Genes Dev. 2013; 27:351–54. 10.1101/gad.214189.113. 23431052 PMC3589552

[84] Magner DB, Wollam J, Shen Y, Hoppe C, Li D, Latza C, Rottiers V, Hutter H, Antebi A The NHR-8 nuclear receptor regulates cholesterol and bile acid homeostasis in C. elegans. Cell Metab. 2013; 18:212–24. 10.1016/j.cmet.2013.07.007. 23931753 PMC3909615

[85] Naudí A, Jové M, Ayala V, Portero-Otín M, Barja G, Pamplona R Membrane lipid unsaturation as physiological adaptation to animal longevity. Front Physiol. 2013; 4:372. 10.3389/fphys.2013.00372. 24381560 PMC3865700

[86] O’Rourke EJ, Kuballa P, Xavier R, Ruvkun G ω-6 Polyunsaturated fatty acids extend life span through the activation of autophagy. Genes Dev. 2013; 27:429–40. 10.1101/gad.205294.112. 23392608 PMC3589559

[87] Burstein MT, Titorenko VI A mitochondrially targeted compound delays aging in yeast through a mechanism linking mitochondrial membrane lipid metabolism to mitochondrial redox biology. Redox Biol. 2014; 2:305–07. 10.1016/j.redox.2014.01.011. 24563847 PMC3926115

[88] Canaan A, DeFuria J, Perelman E, Schultz V, Seay M, Tuck D, Flavell RA, Snyder MP, Obin MS, Weissman SM Extended lifespan and reduced adiposity in mice lacking the FAT10 gene. Proc Natl Acad Sci U S A. 2014; 111:5313–18. 10.1073/pnas.1323426111. 24706839 PMC3986194

[89] Jové M, Naudí A, Ramírez-Núñez O, Portero-Otín M, Selman C, Withers DJ, Pamplona R Caloric restriction reveals a metabolomic and lipidomic signature in liver of male mice. Aging Cell. 2014; 13:828–37. 10.1111/acel.12241. 25052291 PMC4331741

[90] Mahanti P, Bose N, Bethke A, Judkins JC, Wollam J, Dumas KJ, Zimmerman AM, Campbell SL, Hu PJ, Antebi A, Schroeder FC Comparative metabolomics reveals endogenous ligands of DAF-12, a nuclear hormone receptor, regulating C. elegans development and lifespan. Cell Metab. 2014; 19:73–83. 10.1016/j.cmet.2013.11.024. 24411940 PMC3924769

[91] Minois N, Rockenfeller P, Smith TK, Carmona-Gutierrez D Spermidine feeding decreases age-related locomotor activity loss and induces changes in lipid composition. PLoS One. 2014; 9:e102435. 10.1371/journal.pone.0102435. 25010732 PMC4092136

[92] Folick A, Oakley HD, Yu Y, Armstrong EH, Kumari M, Sanor L, Moore DD, Ortlund EA, Zechner R, Wang MC Aging. Lysosomal signaling molecules regulate longevity in Caenorhabditis elegans. Science. 2015; 347:83–86. 10.1126/science.1258857. 25554789 PMC4425353

[93] Lemieux GA, Ashrafi K Neural Regulatory Pathways of Feeding and Fat in Caenorhabditis elegans. Annu Rev Genet. 2015; 49:413–38. 10.1146/annurev-genet-120213-092244. 26473379

[94] Niso-Santano M, Malik SA, Pietrocola F, Bravo-San Pedro JM, Mariño G, Cianfanelli V, Ben-Younès A, Troncoso R, Markaki M, Sica V, Izzo V, Chaba K, Bauvy C, et al Unsaturated fatty acids induce non-canonical autophagy. EMBO J. 2015; 34:1025–41. 10.15252/embj.201489363. 25586377 PMC4406650

[95] Schroeder EA, Brunet A Lipid Profiles and Signals for Long Life. Trends Endocrinol Metab. 2015; 26:589–92. 10.1016/j.tem.2015.08.007. 26439976 PMC4631627

[96] Aguilaniu H, Fabrizio P, Witting M The Role of Dafachronic Acid Signaling in Development and Longevity in Caenorhabditis elegans: Digging Deeper Using Cutting-Edge Analytical Chemistry. Front Endocrinol (Lausanne). 2016; 7:12. 10.3389/fendo.2016.00012. 26903948 PMC4749721

[97] Kim HE, Grant AR, Simic MS, Kohnz RA, Nomura DK, Durieux J, Riera CE, Sanchez M, Kapernick E, Wolff S, Dillin A Lipid Biosynthesis Coordinates a Mitochondrial-to-Cytosolic Stress Response. Cell. 2016; 166:1539–1552.e16. 10.1016/j.cell.2016.08.027. 27610574 PMC5922983

[98] Lemieux GA, Ashrafi K Investigating Connections between Metabolism, Longevity, and Behavior in Caenorhabditis elegans. Trends Endocrinol Metab. 2016; 27:586–96. 10.1016/j.tem.2016.05.004. 27289335 PMC4958586

[99] Martin-Montalvo A, Sun Y, Diaz-Ruiz A, Ali A, Gutierrez V, Palacios HH, Curtis J, Siendones E, Ariza J, Abulwerdi GA, Sun X, Wang AX, Pearson KJ, et al Cytochrome b 5 reductase and the control of lipid metabolism and healthspan. NPJ Aging Mech Dis. 2016; 2:16006. 10.1038/npjamd.2016.6. 28721264 PMC5515006

[100] Watts JL Using Caenorhabditis elegans to Uncover Conserved Functions of Omega-3 and Omega-6 Fatty Acids. J Clin Med. 2016; 5. 10.3390/jcm5020019. 26848697 PMC4773775

[101] Bozek K, Khrameeva EE, Reznick J, Omerbašić D, Bennett NC, Lewin GR, Azpurua J, Gorbunova V, Seluanov A, Regnard P, Wanert F, Marchal J, Pifferi F, et al Lipidome determinants of maximal lifespan in mammals. Sci Rep. 2017; 7:5. 10.1038/s41598-017-00037-7. . Erratum in: Publisher Correction: Lipidome determinants of maximal lifespan in mammals. [Sci Rep. 2019]. 10.1038/s41598-017-00037-7. 31043659 PMC6494842

[102] Green CL, Mitchell SE, Derous D, Wang Y, Chen L, Han JJ, Promislow DE, Lusseau D, Douglas A, Speakman JR The effects of graded levels of calorie restriction: IX. Global metabolomic screen reveals modulation of carnitines, sphingolipids and bile acids in the liver of C57BL/6 mice. Aging Cell. 2017; 16:529–40. 10.1111/acel.12570. 28139067 PMC5418186

[103] Leonov A, Arlia-Ciommo A, Bourque SD, Koupaki O, Kyryakov P, Dakik P, McAuley M, Medkour Y, Mohammad K, Di Maulo T, Titorenko VI Specific changes in mitochondrial lipidome alter mitochondrial proteome and increase the geroprotective efficiency of lithocholic acid in chronologically aging yeast. Oncotarget. 2017; 8:30672–91. 10.18632/oncotarget.16766. 28410198 PMC5458158

[104] Medkour Y, Dakik P, McAuley M, Mohammad K, Mitrofanova D, Titorenko VI Mechanisms Underlying the Essential Role of Mitochondrial Membrane Lipids in Yeast Chronological Aging. Oxid Med Cell Longev. 2017; 2017:2916985. 10.1155/2017/2916985. 28593023 PMC5448074

[105] Miller KN, Burhans MS, Clark JP, Howell PR, Polewski MA, DeMuth TM, Eliceiri KW, Lindstrom MJ, Ntambi JM, Anderson RM Aging and caloric restriction impact adipose tissue, adiponectin, and circulating lipids. Aging Cell. 2017; 16:497–507. 10.1111/acel.12575. 28156058 PMC5418198

[106] Watts JL, Ristow M Lipid and Carbohydrate Metabolism in Caenorhabditis elegans. Genetics. 2017; 207:413–46. 10.1534/genetics.117.300106. 28978773 PMC5629314

[107] Arlia-Ciommo A, Leonov A, Beach A, Richard VR, Bourque SD, Burstein MT, Kyryakov P, Gomez-Perez A, Koupaki O, Feldman R, Titorenko VI Caloric restriction delays yeast chronological aging by remodeling carbohydrate and lipid metabolism, altering peroxisomal and mitochondrial functionalities, and postponing the onsets of apoptotic and liponecrotic modes of regulated cell death. Oncotarget. 2018; 9:16163–84. 10.18632/oncotarget.24604. 29662634 PMC5882325

[108] Arlia-Ciommo A, Leonov A, Mohammad K, Beach A, Richard VR, Bourque SD, Burstein MT, Goldberg AA, Kyryakov P, Gomez-Perez A, Koupaki O, Titorenko VI Mechanisms through which lithocholic acid delays yeast chronological aging under caloric restriction conditions. Oncotarget. 2018; 9:34945–71. 10.18632/oncotarget.26188. 30405886 PMC6201858

[109] Bárcena C, Quirós PM, Durand S, Mayoral P, Rodríguez F, Caravia XM, Mariño G, Garabaya C, Fernández-García MT, Kroemer G, Freije JM, López-Otín C Methionine Restriction Extends Lifespan in Progeroid Mice and Alters Lipid and Bile Acid Metabolism. Cell Rep. 2018; 24:2392–403. 10.1016/j.celrep.2018.07.089. 30157432 PMC6130051

[110] Das UN Ageing: is there a role for arachidonic acid and other bioactive lipids? A review. J Adv Res. 2018; 11:67–79. 10.1016/j.jare.2018.02.004. 30034877 PMC6052661

[111] Gáliková M, Klepsatel P Obesity and Aging in the Drosophila Model. Int J Mol Sci. 2018; 19:E1896. 10.3390/ijms19071896. 29954158 PMC6073435

[112] Conte M, Martucci M, Sandri M, Franceschi C, Salvioli S The Dual Role of the Pervasive “Fattish” Tissue Remodeling With Age. Front Endocrinol (Lausanne). 2019; 10:114. 10.3389/fendo.2019.00114. 30863366 PMC6400104

[113] Lahiri V, Hawkins WD, Klionsky DJ Watch What You (Self-) Eat: Autophagic Mechanisms that Modulate Metabolism. Cell Metab. 2019; 29:803–26. 10.1016/j.cmet.2019.03.003. 30943392 PMC6450419

[114] Papsdorf K, Brunet A Linking Lipid Metabolism to Chromatin Regulation in Aging. Trends Cell Biol. 2019; 29:97–116. 10.1016/j.tcb.2018.09.004. 30316636 PMC6340780

[115] Pradas I, Jové M, Huynh K, Puig J, Ingles M, Borras C, Viña J, Meikle PJ, Pamplona R Exceptional human longevity is associated with a specific plasma phenotype of ether lipids. Redox Biol. 2019; 21:101127. 10.1016/j.redox.2019.101127. 30711699 PMC6357979

[116] Kurat CF, Natter K, Petschnigg J, Wolinski H, Scheuringer K, Scholz H, Zimmermann R, Leber R, Zechner R, Kohlwein SD Obese yeast: triglyceride lipolysis is functionally conserved from mammals to yeast. J Biol Chem. 2006; 281:491–500. 10.1074/jbc.M508414200. 16267052

[117] Black PN, DiRusso CC Yeast acyl-CoA synthetases at the crossroads of fatty acid metabolism and regulation. Biochim Biophys Acta. 2007; 1771:286–98. 10.1016/j.bbalip.2006.05.003. Erratum in: Biochim Biophys Acta. 2007 Jul;1771(7):911. 10.1016/j.bbalip.2006.05.003. 16798075

[118] Rajakumari S, Grillitsch K, Daum G Synthesis and turnover of non-polar lipids in yeast. Prog Lipid Res. 2008; 47:157–71. 10.1016/j.plipres.2008.01.001. 18258205

[119] Carman GM, Han GS Phosphatidic acid phosphatase, a key enzyme in the regulation of lipid synthesis. J Biol Chem. 2009; 284:2593–97. 10.1074/jbc.R800059200. 18812320 PMC2631973

[120] Kohlwein SD Triacylglycerol homeostasis: insights from yeast. J Biol Chem. 2010; 285:15663–67. 10.1074/jbc.R110.118356. 20231294 PMC2871431

[121] Kohlwein SD Obese and anorexic yeasts: experimental models to understand the metabolic syndrome and lipotoxicity. Biochim Biophys Acta. 2010; 1801:222–29. 10.1016/j.bbalip.2009.12.016. 20056167

[122] Carman GM, Han GS Regulation of phospholipid synthesis in the yeast Saccharomyces cerevisiae. Annu Rev Biochem. 2011; 80:859–83. 10.1146/annurev-biochem-060409-092229. 21275641 PMC3565220

[123] Titorenko VI, Terlecky SR Peroxisome metabolism and cellular aging. Traffic. 2011; 12:252–59. 10.1111/j.1600-0854.2010.01144.x. 21083858 PMC3077116

[124] Beach A, Burstein MT, Richard VR, Leonov A, Levy S, Titorenko VI Integration of peroxisomes into an endomembrane system that governs cellular aging. Front Physiol. 2012; 3:283. 10.3389/fphys.2012.00283. 22936916 PMC3424522

[125] Henry SA, Kohlwein SD, Carman GM Metabolism and regulation of glycerolipids in the yeast Saccharomyces cerevisiae. Genetics. 2012; 190:317–49. 10.1534/genetics.111.130286. 22345606 PMC3276621

[126] Zechner R, Zimmermann R, Eichmann TO, Kohlwein SD, Haemmerle G, Lass A, Madeo F FAT SIGNALS-lipases and lipolysis in lipid metabolism and signaling. Cell Metab. 2012; 15:279–91. 10.1016/j.cmet.2011.12.018. 22405066 PMC3314979

[127] Beach A, Titorenko VI Essential roles of peroxisomally produced and metabolized biomolecules in regulating yeast longevity. Subcell Biochem. 2013; 69:153–67. 10.1007/978-94-007-6889-5_9. 23821148

[128] Horvath SE, Daum G Lipids of mitochondria. Prog Lipid Res. 2013; 52:590–614. 10.1016/j.plipres.2013.07.002. 24007978

[129] Kohlwein SD, Veenhuis M, van der Klei IJ Lipid droplets and peroxisomes: key players in cellular lipid homeostasis or a matter of fat-store ‘em up or burn ‘em down. Genetics. 2013; 193:1–50. 10.1534/genetics.112.143362. 23275493 PMC3527239

[130] Leonov A, Titorenko VI A network of interorganellar communications underlies cellular aging. IUBMB Life. 2013; 65:665–74. 10.1002/iub.1183. 23818261

[131] Pascual F, Carman GM Phosphatidate phosphatase, a key regulator of lipid homeostasis. Biochim Biophys Acta. 2013; 1831:514–22. 10.1016/j.bbalip.2012.08.006. 22910056 PMC3549317

[132] Baile MG, Lu YW, Claypool SM The topology and regulation of cardiolipin biosynthesis and remodeling in yeast. Chem Phys Lipids. 2014; 179:25–31. 10.1016/j.chemphyslip.2013.10.008. 24184646 PMC3947685

[133] Klug L, Daum G Yeast lipid metabolism at a glance. FEMS Yeast Res. 2014; 14:369–88. 10.1111/1567-1364.12141. 24520995

[134] Koch B, Schmidt C, Daum G Storage lipids of yeasts: a survey of nonpolar lipid metabolism in Saccharomyces cerevisiae, Pichia pastoris, and Yarrowia lipolytica. FEMS Microbiol Rev. 2014; 38:892–915. 10.1111/1574-6976.12069. 24597968

[135] Gao Q, Goodman JM The lipid droplet-a well-connected organelle. Front Cell Dev Biol. 2015; 3:49. 10.3389/fcell.2015.00049. 26322308 PMC4533013

[136] Hashemi HF, Goodman JM The life cycle of lipid droplets. Curr Opin Cell Biol. 2015; 33:119–24. 10.1016/j.ceb.2015.02.002. 25703629 PMC4380764

[137] Arlia-Ciommo A, Svistkova V, Mohtashami S, Titorenko VI A novel approach to the discovery of anti-tumor pharmaceuticals: searching for activators of liponecrosis. Oncotarget. 2016; 7:5204–25. 10.18632/oncotarget.6440. 26636650 PMC4868681

[138] Dakik P, Titorenko VI Communications between Mitochondria, the Nucleus, Vacuoles, Peroxisomes, the Endoplasmic Reticulum, the Plasma Membrane, Lipid Droplets, and the Cytosol during Yeast Chronological Aging. Front Genet. 2016; 7:177. 10.3389/fgene.2016.00177. 27729926 PMC5037234

[139] Fernández-Murray JP, McMaster CR Lipid synthesis and membrane contact sites: a crossroads for cellular physiology. J Lipid Res. 2016; 57:1789–805. 10.1194/jlr.R070920. 27521373 PMC5036376

[140] Barbosa AD, Siniossoglou S Function of lipid droplet-organelle interactions in lipid homeostasis. Biochim Biophys Acta Mol Cell Res. 2017; 1864:1459–68. 10.1016/j.bbamcr.2017.04.001. 28390906

[141] Dimmer KS, Rapaport D Mitochondrial contact sites as platforms for phospholipid exchange. Biochim Biophys Acta Mol Cell Biol Lipids. 2017; 1862:69–80. 10.1016/j.bbalip.2016.07.010. 27477677

[142] Elbaz-Alon Y Mitochondria-organelle contact sites: the plot thickens. Biochem Soc Trans. 2017; 45:477–88. 10.1042/BST20160130. 28408488

[143] Mårtensson CU, Doan KN, Becker T Effects of lipids on mitochondrial functions. Biochim Biophys Acta Mol Cell Biol Lipids. 2017; 1862:102–13. 10.1016/j.bbalip.2016.06.015. 27349299

[144] Tatsuta T, Langer T Intramitochondrial phospholipid trafficking. Biochim Biophys Acta Mol Cell Biol Lipids. 2017; 1862:81–89. 10.1016/j.bbalip.2016.08.006. 27542541

[145] Csordás G, Weaver D, Hajnóczky G Endoplasmic Reticulum-Mitochondrial Contactology: Structure and Signaling Functions. Trends Cell Biol. 2018; 28:523–40. 10.1016/j.tcb.2018.02.009. 29588129 PMC6005738

[146] Graef M Lipid droplet-mediated lipid and protein homeostasis in budding yeast. FEBS Lett. 2018; 592:1291–303. 10.1002/1873-3468.12996. 29397034 PMC5947121

[147] Renne MF, de Kroon AI The role of phospholipid molecular species in determining the physical properties of yeast membranes. FEBS Lett. 2018; 592:1330–45. 10.1002/1873-3468.12944. 29265372 PMC5947837

[148] Simmen T, Herrera-Cruz MS Plastic mitochondria-endoplasmic reticulum (ER) contacts use chaperones and tethers to mould their structure and signaling. Curr Opin Cell Biol. 2018; 53:61–69. 10.1016/j.ceb.2018.04.014. 29870872

[149] Tamura Y, Kawano S, Endo T Organelle contact zones as sites for lipid transfer. J Biochem. 2019; 165:115–23. 10.1093/jb/mvy088. 30371789

[150] Richard VR, Beach A, Piano A, Leonov A, Feldman R, Burstein MT, Kyryakov P, Gomez-Perez A, Arlia-Ciommo A, Baptista S, Campbell C, Goncharov D, Pannu S, et al Mechanism of liponecrosis, a distinct mode of programmed cell death. Cell Cycle. 2014; 13:3707–26. 10.4161/15384101.2014.965003. 25483081 PMC4613542

[151] Sheibani S, Richard VR, Beach A, Leonov A, Feldman R, Mattie S, Khelghatybana L, Piano A, Greenwood M, Vali H, Titorenko VI Macromitophagy, neutral lipids synthesis, and peroxisomal fatty acid oxidation protect yeast from “liponecrosis”, a previously unknown form of programmed cell death. Cell Cycle. 2014; 13:138–47. 10.4161/cc.26885. 24196447 PMC3925724

[152] Carmona-Gutierrez D, Bauer MA, Zimmermann A, Aguilera A, Austriaco N, Ayscough K, Balzan R, Bar-Nun S, Barrientos A, Belenky P, Blondel M, Braun RJ, Breitenbach M, et al Guidelines and recommendations on yeast cell death nomenclature. Microb Cell. 2018; 5:4–31. 10.15698/mic2018.01.607. 29354647 PMC5772036

[153] Mohammad K, Dakik P, Medkour Y, McAuley M, Mitrofanova D, Titorenko VI Yeast Cells Exposed to Exogenous Palmitoleic Acid Either Adapt to Stress and Survive or Commit to Regulated Liponecrosis and Die. Oxid Med Cell Longev. 2018; 2018:3074769. 10.1155/2018/3074769. 29636840 PMC5831759

[154] Rockenfeller P, Gourlay CW Lipotoxicty in yeast: a focus on plasma membrane signalling and membrane contact sites. FEMS Yeast Res. 2018; 18. 10.1093/femsyr/foy034. 29718175 PMC5905628

[155] Rockenfeller P, Smolnig M, Diessl J, Bashir M, Schmiedhofer V, Knittelfelder O, Ring J, Franz J, Foessl I, Khan MJ, Rost R, Graier WF, Kroemer G, et al Diacylglycerol triggers Rim101 pathway-dependent necrosis in yeast: a model for lipotoxicity. Cell Death Differ. 2018; 25:767–83. 10.1038/s41418-017-0014-2. 29230001 PMC5864183

[156] Borradaile NM, Han X, Harp JD, Gale SE, Ory DS, Schaffer JE Disruption of endoplasmic reticulum structure and integrity in lipotoxic cell death. J Lipid Res. 2006; 47:2726–37. 10.1194/jlr.M600299-JLR200. 16960261

[157] Deguil J, Pineau L, Rowland Snyder EC, Dupont S, Beney L, Gil A, Frapper G, Ferreira T Modulation of lipid-induced ER stress by fatty acid shape. Traffic. 2011; 12:349–62. 10.1111/j.1600-0854.2010.01150.x. 21143717

[158] Fu S, Yang L, Li P, Hofmann O, Dicker L, Hide W, Lin X, Watkins SM, Ivanov AR, Hotamisligil GS Aberrant lipid metabolism disrupts calcium homeostasis causing liver endoplasmic reticulum stress in obesity. Nature. 2011; 473:528–31. 10.1038/nature09968. 21532591 PMC3102791

[159] Promlek T, Ishiwata-Kimata Y, Shido M, Sakuramoto M, Kohno K, Kimata Y Membrane aberrancy and unfolded proteins activate the endoplasmic reticulum stress sensor Ire1 in different ways. Mol Biol Cell. 2011; 22:3520–32. 10.1091/mbc.e11-04-0295. 21775630 PMC3172275

[160] Fu S, Watkins SM, Hotamisligil GS The role of endoplasmic reticulum in hepatic lipid homeostasis and stress signaling. Cell Metab. 2012; 15:623–34. 10.1016/j.cmet.2012.03.007. 22560215

[161] Thibault G, Shui G, Kim W, McAlister GC, Ismail N, Gygi SP, Wenk MR, Ng DT The membrane stress response buffers lethal effects of lipid disequilibrium by reprogramming the protein homeostasis network. Mol Cell. 2012; 48:16–27. 10.1016/j.molcel.2012.08.016. 23000174 PMC3496426

[162] Cui W, Ma J, Wang X, Yang W, Zhang J, Ji Q Free fatty acid induces endoplasmic reticulum stress and apoptosis of β-cells by Ca2+/calpain-2 pathways. PLoS One. 2013; 8:e59921. 10.1371/journal.pone.0059921. 23527285 PMC3604010

[163] Lagace TA, Ridgway ND The role of phospholipids in the biological activity and structure of the endoplasmic reticulum. Biochim Biophys Acta. 2013; 1833:2499–510. 10.1016/j.bbamcr.2013.05.018. 23711956

[164] Surma MA, Klose C, Peng D, Shales M, Mrejen C, Stefanko A, Braberg H, Gordon DE, Vorkel D, Ejsing CS, Farese R Jr, Simons K, Krogan NJ, Ernst R A lipid E-MAP identifies Ubx2 as a critical regulator of lipid saturation and lipid bilayer stress. Mol Cell. 2013; 51:519–30. 10.1016/j.molcel.2013.06.014. 23891562 PMC3791312

[165] Volmer R, van der Ploeg K, Ron D Membrane lipid saturation activates endoplasmic reticulum unfolded protein response transducers through their transmembrane domains. Proc Natl Acad Sci U S A. 2013; 110:4628–33. 10.1073/pnas.1217611110. 23487760 PMC3606975

[166] Wu H, Ng BS, Thibault G Endoplasmic reticulum stress response in yeast and humans. Biosci Rep. 2014; 34. 10.1042/BSR20140058. 24909749 PMC4076835

[167] Volmer R, Ron D Lipid-dependent regulation of the unfolded protein response. Curr Opin Cell Biol. 2015; 33:67–73. 10.1016/j.ceb.2014.12.002. 25543896 PMC4376399

[168] Covino R, Ballweg S, Stordeur C, Michaelis JB, Puth K, Wernig F, Bahrami A, Ernst AM, Hummer G, Ernst R A Eukaryotic Sensor for Membrane Lipid Saturation. Mol Cell. 2016; 63:49–59. 10.1016/j.molcel.2016.05.015. 27320200

[169] Akoumi A, Haffar T, Mousterji M, Kiss RS, Bousette N Palmitate mediated diacylglycerol accumulation causes endoplasmic reticulum stress, Plin2 degradation, and cell death in H9C2 cardiomyoblasts. Exp Cell Res. 2017; 354:85–94. 10.1016/j.yexcr.2017.03.032. 28336294

[170] Chen E, Tsai TH, Li L, Saha P, Chan L, Chang BH PLIN2 is a Key Regulator of the Unfolded Protein Response and Endoplasmic Reticulum Stress Resolution in Pancreatic β Cells. Sci Rep. 2017; 7:40855. 10.1038/srep40855. 28102311 PMC5244387

[171] Halbleib K, Pesek K, Covino R, Hofbauer HF, Wunnicke D, Hänelt I, Hummer G, Ernst R Activation of the Unfolded Protein Response by Lipid Bilayer Stress. Mol Cell. 2017; 67:673–684.e8. 10.1016/j.molcel.2017.06.012. 28689662

[172] Covino R, Hummer G, Ernst R Integrated Functions of Membrane Property Sensors and a Hidden Side of the Unfolded Protein Response. Mol Cell. 2018; 71:458–67. 10.1016/j.molcel.2018.07.019. 30075144

[173] Ho N, Xu C, Thibault G From the unfolded protein response to metabolic diseases - lipids under the spotlight. J Cell Sci. 2018; 131. 10.1242/jcs.199307. 29439157

[174] Koh JH, Wang L, Beaudoin-Chabot C, Thibault G Lipid bilayer stress-activated IRE-1 modulates autophagy during endoplasmic reticulum stress. J Cell Sci. 2018; 131. 10.1242/jcs.217992. 30333136

[175] Shyu P Jr, Wong XF, Crasta K, Thibault G Dropping in on lipid droplets: insights into cellular stress and cancer. Biosci Rep. 2018; 38. 10.1042/BSR20180764. 30111611 PMC6146295

[176] Almanza A, Carlesso A, Chintha C, Creedican S, Doultsinos D, Leuzzi B, Luís A, McCarthy N, Montibeller L, More S, Papaioannou A, Püschel F, Sassano ML, et al Endoplasmic reticulum stress signalling - from basic mechanisms to clinical applications. FEBS J. 2019; 286:241–78. 10.1111/febs.14608. 30027602 PMC7379631

[177] Cho H, Stanzione F, Oak A, Kim GH, Yerneni S, Qi L, Sum AK, Chan C Intrinsic Structural Features of the Human IRE1α Transmembrane Domain Sense Membrane Lipid Saturation. Cell Rep. 2019; 27:307–320.e5. 10.1016/j.celrep.2019.03.017. 30943411 PMC6467502

[178] Fun XH, Thibault G Lipid bilayer stress and proteotoxic stress-induced unfolded protein response deploy divergent transcriptional and non-transcriptional programmes. Biochim Biophys Acta Mol Cell Biol Lipids. 2019 4 24. 10.1016/j.bbalip.2019.04.009. [Epub ahead of print]. 31028913

[179] Hariri H, Speer N, Bowerman J, Rogers S, Fu G, Reetz E, Datta S, Feathers JR, Ugrankar R, Nicastro D, Henne WM Mdm1 maintains endoplasmic reticulum homeostasis by spatially regulating lipid droplet biogenesis. J Cell Biol. 2019; 218:1319–34. 10.1083/jcb.201808119. 30808705 PMC6446837

[180] Ron D, Walter P Signal integration in the endoplasmic reticulum unfolded protein response. Nat Rev Mol Cell Biol. 2007; 8:519–29. 10.1038/nrm2199. 17565364

[181] Jonikas MC, Collins SR, Denic V, Oh E, Quan EM, Schmid V, Weibezahn J, Schwappach B, Walter P, Weissman JS, Schuldiner M Comprehensive characterization of genes required for protein folding in the endoplasmic reticulum. Science. 2009; 323:1693–97. 10.1126/science.1167983. 19325107 PMC2877488

[182] Walter P, Ron D The unfolded protein response: from stress pathway to homeostatic regulation. Science. 2011; 334:1081–86. 10.1126/science.1209038. 22116877

[183] Araki K, Nagata K Protein folding and quality control in the ER. Cold Spring Harb Perspect Biol. 2011; 3:a007526. 10.1101/cshperspect.a007526. 21875985 PMC3220362

[184] Gardner BM, Pincus D, Gotthardt K, Gallagher CM, Walter P Endoplasmic reticulum stress sensing in the unfolded protein response. Cold Spring Harb Perspect Biol. 2013; 5:a013169. 10.1101/cshperspect.a013169. 23388626 PMC3578356

[185] Higuchi-Sanabria R, Frankino PA, Paul JW 3rd, Tronnes SU, Dillin A A Futile Battle? Protein Quality Control and the Stress of Aging. Dev Cell. 2018; 44:139–63. 10.1016/j.devcel.2017.12.020. 29401418 PMC5896312

[186] Karagöz GE, Acosta-Alvear D, Walter P The Unfolded Protein Response: Detecting and Responding to Fluctuations in the Protein-Folding Capacity of the Endoplasmic Reticulum. Cold Spring Harb Perspect Biol. 2019; 11. 10.1101/cshperspect.a033886. 30670466 PMC6719602

[187] Salminen A, Kaarniranta K ER stress and hormetic regulation of the aging process. Ageing Res Rev. 2010; 9:211–17. 10.1016/j.arr.2010.04.003. 20416402

[188] Hou J, Tang H, Liu Z, Österlund T, Nielsen J, Petranovic D Management of the endoplasmic reticulum stress by activation of the heat shock response in yeast. FEMS Yeast Res. 2014; 14:481–94. 10.1111/1567-1364.12125. 24237754

[189] Labunskyy VM, Gerashchenko MV, Delaney JR, Kaya A, Kennedy BK, Kaeberlein M, Gladyshev VN Lifespan extension conferred by endoplasmic reticulum secretory pathway deficiency requires induction of the unfolded protein response. PLoS Genet. 2014; 10:e1004019. 10.1371/journal.pgen.1004019. 24391512 PMC3879150

[190] Cui HJ, Liu XG, McCormick M, Wasko BM, Zhao W, He X, Yuan Y, Fang BX, Sun XR, Kennedy BK, Suh Y, Zhou ZJ, Kaeberlein M, Feng WL PMT1 deficiency enhances basal UPR activity and extends replicative lifespan of Saccharomyces cerevisiae. Age (Dordr). 2015; 37:9788. 10.1007/s11357-015-9788-7. 25936926 PMC4417673

[191] Weindling E, Bar-Nun S Sir2 links the unfolded protein response and the heat shock response in a stress response network. Biochem Biophys Res Commun. 2015; 457:473–78. 10.1016/j.bbrc.2015.01.021. 25600811

[192] Piperi C, Adamopoulos C, Papavassiliou AG XBP1: A Pivotal Transcriptional Regulator of Glucose and Lipid Metabolism. Trends Endocrinol Metab. 2016; 27:119–22. 10.1016/j.tem.2016.01.001. 26803729

[193] Cohen N, Breker M, Bakunts A, Pesek K, Chas A, Argemí J, Orsi A, Gal L, Chuartzman S, Wigelman Y, Jonas F, Walter P, Ernst R, et al Iron affects Ire1 clustering propensity and the amplitude of endoplasmic reticulum stress signaling. J Cell Sci. 2017; 130:3222–33. 10.1242/jcs.201715. 28794014 PMC5665437

[194] Guzel E, Arlier S, Guzeloglu-Kayisli O, Tabak MS, Ekiz T, Semerci N, Larsen K, Schatz F, Lockwood CJ, Kayisli UA Endoplasmic Reticulum Stress and Homeostasis in Reproductive Physiology and Pathology. Int J Mol Sci. 2017; 18. 10.3390/ijms18040792. 28397763 PMC5412376

[195] Martínez G, Duran-Aniotz C, Cabral-Miranda F, Vivar JP, Hetz C Endoplasmic reticulum proteostasis impairment in aging. Aging Cell. 2017; 16:615–23. 10.1111/acel.12599. 28436203 PMC5506418

[196] Postnikoff SD, Johnson JE, Tyler JK The integrated stress response in budding yeast lifespan extension. Microb Cell. 2017; 4:368–75. 10.15698/mic2017.11.597. 29167799 PMC5695854

[197] Remondelli P, Renna M The Endoplasmic Reticulum Unfolded Protein Response in Neurodegenerative Disorders and Its Potential Therapeutic Significance. Front Mol Neurosci. 2017; 10:187. 10.3389/fnmol.2017.00187. 28670265 PMC5472670

[198] Moon HW, Han HG, Jeon YJ Protein Quality Control in the Endoplasmic Reticulum and Cancer. Int J Mol Sci. 2018; 19. 10.3390/ijms19103020. 30282948 PMC6213883

[199] Beaupere C, Labunskyy VM (Un)folding mechanisms of adaptation to ER stress: lessons from aneuploidy. Curr Genet. 2019; 65:467–71. 10.1007/s00294-018-0914-9. 30511161 PMC6421085

[200] Chadwick SR, Lajoie P Endoplasmic Reticulum Stress Coping Mechanisms and Lifespan Regulation in Health and Diseases. Front Cell Dev Biol. 2019; 7:84. 10.3389/fcell.2019.00084. 31231647 PMC6558375

[201] Lehrbach NJ, Ruvkun G Endoplasmic reticulum-associated SKN-1A/Nrf1 mediates a cytoplasmic unfolded protein response and promotes longevity. Elife. 2019; 8. 10.7554/eLife.44425. 30973820 PMC6459674

[202] Schmidt RM, Schessner JP, Borner GH, Schuck S The proteasome biogenesis regulator Rpn4 cooperates with the unfolded protein response to promote ER stress resistance. Elife. 2019; 8. 10.7554/eLife.43244. 30865586 PMC6415940

[203] Ha EE, Frohman MA Regulation of mitochondrial morphology by lipids. Biofactors. 2014; 40:419–24. 10.1002/biof.1169. 24771456 PMC4146713

[204] Valencak TG, Azzu V Making heads or tails of mitochondrial membranes in longevity and aging: a role for comparative studies. Longev Healthspan. 2014; 3:3. 10.1186/2046-2395-3-3. 24588808 PMC3996024

[205] Beach A, Richard VR, Bourque S, Boukh-Viner T, Kyryakov P, Gomez-Perez A, Arlia-Ciommo A, Feldman R, Leonov A, Piano A, Svistkova V, Titorenko VI Lithocholic bile acid accumulated in yeast mitochondria orchestrates a development of an anti-aging cellular pattern by causing age-related changes in cellular proteome. Cell Cycle. 2015; 14:1643–56. 10.1080/15384101.2015.1026493. 25839782 PMC4614269

[206] Vögtle FN, Keller M, Taskin AA, Horvath SE, Guan XL, Prinz C, Opalińska M, Zorzin C, van der Laan M, Wenk MR, Schubert R, Wiedemann N, Holzer M, Meisinger C The fusogenic lipid phosphatidic acid promotes the biogenesis of mitochondrial outer membrane protein Ugo1. J Cell Biol. 2015; 210:951–60. 10.1083/jcb.201506085. 26347140 PMC4576865

[207] Medkour Y, Titorenko VI Mitochondria operate as signaling platforms in yeast aging. Aging (Albany NY). 2016; 8:212–13. 10.18632/aging.100914. 26928478 PMC4789576

[208] Ademowo OS, Dias HK, Burton DG, Griffiths HR Lipid (per) oxidation in mitochondria: an emerging target in the ageing process? Biogerontology. 2017; 18:859–79. 10.1007/s10522-017-9710-z. 28540446 PMC5684309

[209] Pollard AK, Ortori CA, Stöger R, Barrett DA, Chakrabarti L Mouse mitochondrial lipid composition is defined by age in brain and muscle. Aging (Albany NY). 2017; 9:986–98. 10.18632/aging.101204. 28325886 PMC5391243

[210] Johnson DR, Knoll LJ, Levin DE, Gordon JI Saccharomyces cerevisiae contains four fatty acid activation (FAA) genes: an assessment of their role in regulating protein N-myristoylation and cellular lipid metabolism. J Cell Biol. 1994; 127:751–62. 10.1083/jcb.127.3.751. 7962057 PMC2120220

[211] Athenstaedt K, Daum G Biosynthesis of phosphatidic acid in lipid particles and endoplasmic reticulum of Saccharomyces cerevisiae. J Bacteriol. 1997; 179:7611–16. 10.1128/jb.179.24.7611-7616.1997. 9401016 PMC179720

[212] Athenstaedt K, Daum G Phosphatidic acid, a key intermediate in lipid metabolism. Eur J Biochem. 1999; 266:1–16. 10.1046/j.1432-1327.1999.00822.x. 10542045

[213] Benghezal M, Roubaty C, Veepuri V, Knudsen J, Conzelmann A SLC1 and SLC4 encode partially redundant acyl-coenzyme A 1-acylglycerol-3-phosphate O-acyltransferases of budding yeast. J Biol Chem. 2007; 282:30845–55. 10.1074/jbc.M702719200. 17675291

[214] Riekhof WR, Wu J, Jones JL, Voelker DR Identification and characterization of the major lysophosphatidylethanolamine acyltransferase in Saccharomyces cerevisiae. J Biol Chem. 2007; 282:28344–52. 10.1074/jbc.M705256200. 17652094

[215] Athenstaedt K, Daum G YMR313c/TGL3 encodes a novel triacylglycerol lipase located in lipid particles of Saccharomyces cerevisiae. J Biol Chem. 2003; 278:23317–23. 10.1074/jbc.M302577200. 12682047

[216] Athenstaedt K, Daum G Tgl4p and Tgl5p, two triacylglycerol lipases of the yeast Saccharomyces cerevisiae are localized to lipid particles. J Biol Chem. 2005; 280:37301–09. 10.1074/jbc.M507261200. 16135509

[217] Köffel R, Tiwari R, Falquet L, Schneiter R The Saccharomyces cerevisiae YLL012/YEH1, YLR020/YEH2, and TGL1 genes encode a novel family of membrane-anchored lipases that are required for steryl ester hydrolysis. Mol Cell Biol. 2005; 25:1655–68. 10.1128/MCB.25.5.1655-1668.2005. 15713625 PMC549362

[218] Mukaka MM Statistics corner: A guide to appropriate use of correlation coefficient in medical research. Malawi Med J. 2012; 24:69–71. 23638278 PMC3576830

[219] Eisenberg T, Carmona-Gutierrez D, Büttner S, Tavernarakis N, Madeo F Necrosis in yeast. Apoptosis. 2010; 15:257–68. 10.1007/s10495-009-0453-4. 20238475

[220] Jesch SA, Liu P, Zhao X, Wells MT, Henry SA Multiple endoplasmic reticulum-to-nucleus signaling pathways coordinate phospholipid metabolism with gene expression by distinct mechanisms. J Biol Chem. 2006; 281:24070–83. 10.1074/jbc.M604541200. 16777852

[221] Zanchin NI, McCarthy JE Characterization of the *in vivo* phosphorylation sites of the mRNA.cap-binding complex proteins eukaryotic initiation factor-4E and p20 in Saccharomyces cerevisiae. J Biol Chem. 1995; 270:26505–10. 10.1074/jbc.270.44.26505. 7592868

[222] Altmann M, Schmitz N, Berset C, Trachsel H A novel inhibitor of cap-dependent translation initiation in yeast: p20 competes with eIF4G for binding to eIF4E. EMBO J. 1997; 16:1114–21. 10.1093/emboj/16.5.1114. 9118949 PMC1169710

[223] Wade CH, Umbarger MA, McAlear MA The budding yeast rRNA and ribosome biosynthesis (RRB) regulon contains over 200 genes. Yeast. 2006; 23:293–306. 10.1002/yea.1353. 16544271

[224] D’Silva S, Haider SJ, Phizicky EM A domain of the actin binding protein Abp140 is the yeast methyltransferase responsible for 3-methylcytidine modification in the tRNA anti-codon loop. RNA. 2011; 17:1100–10. 10.1261/rna.2652611. 21518804 PMC3096042

[225] Noma A, Yi S, Katoh T, Takai Y, Suzuki T, Suzuki T Actin-binding protein ABP140 is a methyltransferase for 3-methylcytidine at position 32 of tRNAs in Saccharomyces cerevisiae. RNA. 2011; 17:1111–19. 10.1261/rna.2653411. 21518805 PMC3096043

[226] Sammons MA, Samir P, Link AJ Saccharomyces cerevisiae Gis2 interacts with the translation machinery and is orthogonal to myotonic dystrophy type 2 protein ZNF9. Biochem Biophys Res Commun. 2011; 406:13–19. 10.1016/j.bbrc.2011.01.086. 21277287

[227] Rojas M, Farr GW, Fernandez CF, Lauden L, McCormack JC, Wolin SL Yeast Gis2 and its human ortholog CNBP are novel components of stress-induced RNP granules. PLoS One. 2012; 7:e52824. 10.1371/journal.pone.0052824. 23285195 PMC3528734

[228] Stelter P, Huber FM, Kunze R, Flemming D, Hoelz A, Hurt E Coordinated ribosomal L4 protein assembly into the pre-ribosome is regulated by its eukaryote-specific extension. Mol Cell. 2015; 58:854–62. 10.1016/j.molcel.2015.03.029. 25936803 PMC6742479

[229] Fraenkel DG Yeast intermediary metabolism Cold Spring Harbor: Cold Spring Harbor Laboratory Press; 2011.

[230] Stearns SC, Kaiser M The effects of enhanced expression of elongation factor EF-1 alpha on lifespan in Drosophila melanogaster. IV. A summary of three experiments. Genetica. 1993; 91:167–82. 10.1007/BF01435996. 8125267

[231] Shikama N, Ackermann R, Brack C Protein synthesis elongation factor EF-1 alpha expression and longevity in Drosophila melanogaster. Proc Natl Acad Sci U S A. 1994; 91:4199–203. 10.1073/pnas.91.10.4199. 8183891 PMC43752

[232] Hamilton B, Dong Y, Shindo M, Liu W, Odell I, Ruvkun G, Lee SS A systematic RNAi screen for longevity genes in C. elegans. Genes Dev. 2005; 19:1544–55. 10.1101/gad.1308205. 15998808 PMC1172061

[233] Kaeberlein M, Powers RW 3rd, Steffen KK, Westman EA, Hu D, Dang N, Kerr EO, Kirkland KT, Fields S, Kennedy BK Regulation of yeast replicative life span by TOR and Sch9 in response to nutrients. Science. 2005; 310:1193–96. 10.1126/science.1115535. 16293764

[234] Teleman AA, Chen YW, Cohen SM 4E-BP functions as a metabolic brake used under stress conditions but not during normal growth. Genes Dev. 2005; 19:1844–48. 10.1101/gad.341505. 16103212 PMC1186183

[235] Henderson ST, Bonafè M, Johnson TE daf-16 protects the nematode Caenorhabditis elegans during food deprivation. J Gerontol A Biol Sci Med Sci. 2006; 61:444–60. 10.1093/gerona/61.5.444. 16720740

[236] Chen D, Pan KZ, Palter JE, Kapahi P Longevity determined by developmental arrest genes in Caenorhabditis elegans. Aging Cell. 2007; 6:525–33. 10.1111/j.1474-9726.2007.00305.x. 17521386 PMC2746107

[237] Curran SP, Ruvkun G Lifespan regulation by evolutionarily conserved genes essential for viability. PLoS Genet. 2007; 3:e56. 10.1371/journal.pgen.0030056. 17411345 PMC1847696

[238] Hansen M, Taubert S, Crawford D, Libina N, Lee SJ, Kenyon C Lifespan extension by conditions that inhibit translation in Caenorhabditis elegans. Aging Cell. 2007; 6:95–110. 10.1111/j.1474-9726.2006.00267.x. 17266679

[239] Hipkiss AR On why decreasing protein synthesis can increase lifespan. Mech Ageing Dev. 2007; 128:412–14. 10.1016/j.mad.2007.03.002. 17452047

[240] Pan KZ, Palter JE, Rogers AN, Olsen A, Chen D, Lithgow GJ, Kapahi P Inhibition of mRNA translation extends lifespan in Caenorhabditis elegans. Aging Cell. 2007; 6:111–19. 10.1111/j.1474-9726.2006.00266.x. 17266680 PMC2745345

[241] Syntichaki P, Troulinaki K, Tavernarakis N eIF4E function in somatic cells modulates ageing in Caenorhabditis elegans. Nature. 2007; 445:922–26. 10.1038/nature05603. 17277769

[242] Smith ED, Tsuchiya M, Fox LA, Dang N, Hu D, Kerr EO, Johnston ED, Tchao BN, Pak DN, Welton KL, Promislow DE, Thomas JH, Kaeberlein M, Kennedy BK Quantitative evidence for conserved longevity pathways between divergent eukaryotic species. Genome Res. 2008; 18:564–70. 10.1101/gr.074724.107. 18340043 PMC2279244

[243] Steffen KK, MacKay VL, Kerr EO, Tsuchiya M, Hu D, Fox LA, Dang N, Johnston ED, Oakes JA, Tchao BN, Pak DN, Fields S, Kennedy BK, Kaeberlein M Yeast life span extension by depletion of 60s ribosomal subunits is mediated by Gcn4. Cell. 2008; 133:292–302. 10.1016/j.cell.2008.02.037. 18423200 PMC2749658

[244] Tavernarakis N Ageing and the regulation of protein synthesis: a balancing act? Trends Cell Biol. 2008; 18:228–35. 10.1016/j.tcb.2008.02.004. 18346894

[245] Tohyama D, Yamaguchi A, Yamashita T Inhibition of a eukaryotic initiation factor (eIF2Bdelta/F11A3.2) during adulthood extends lifespan in Caenorhabditis elegans. FASEB J. 2008; 22:4327–37. 10.1096/fj.08-112953. 18728216

[246] Kennedy BK, Kaeberlein M Hot topics in aging research: protein translation, 2009. Aging Cell. 2009; 8:617–23. 10.1111/j.1474-9726.2009.00522.x. 19747234 PMC3673879

[247] Demontis F, Perrimon N FOXO/4E-BP signaling in Drosophila muscles regulates organism-wide proteostasis during aging. Cell. 2010; 143:813–25. 10.1016/j.cell.2010.10.007. 21111239 PMC3066043

[248] Rogers AN, Chen D, McColl G, Czerwieniec G, Felkey K, Gibson BW, Hubbard A, Melov S, Lithgow GJ, Kapahi P Life span extension via eIF4G inhibition is mediated by posttranscriptional remodeling of stress response gene expression in C. elegans. Cell Metab. 2011; 14:55–66. 10.1016/j.cmet.2011.05.010. 21723504 PMC3220185

[249] Pestov DG, Shcherbik N Rapid cytoplasmic turnover of yeast ribosomes in response to rapamycin inhibition of TOR. Mol Cell Biol. 2012; 32:2135–44. 10.1128/MCB.06763-11. 22451491 PMC3372233

[250] Leprivier G, Remke M, Rotblat B, Dubuc A, Mateo AR, Kool M, Agnihotri S, El-Naggar A, Yu B, Somasekharan SP, Faubert B, Bridon G, Tognon CE, et al The eEF2 kinase confers resistance to nutrient deprivation by blocking translation elongation. Cell. 2013; 153:1064–79. 10.1016/j.cell.2013.04.055. 23706743 PMC4395874

[251] Cattie DJ, Richardson CE, Reddy KC, Ness-Cohn EM, Droste R, Thompson MK, Gilbert WV, Kim DH Mutations in Nonessential eIF3k and eIF3l Genes Confer Lifespan Extension and Enhanced Resistance to ER Stress in Caenorhabditis elegans. PLoS Genet. 2016; 12:e1006326. 10.1371/journal.pgen.1006326. 27690135 PMC5045169

[252] Gonskikh Y, Polacek N Alterations of the translation apparatus during aging and stress response. Mech Ageing Dev. 2017; 168:30–36. 10.1016/j.mad.2017.04.003. 28414025

[253] Mathis AD, Naylor BC, Carson RH, Evans E, Harwell J, Knecht J, Hexem E, Peelor FF 3rd, Miller BF, Hamilton KL, Transtrum MK, Bikman BT, Price JC Mechanisms of In Vivo Ribosome Maintenance Change in Response to Nutrient Signals. Mol Cell Proteomics. 2017; 16:243–54. 10.1074/mcp.M116.063255. 27932527 PMC5294211

[254] Solis GM, Kardakaris R, Valentine ER, Bar-Peled L, Chen AL, Blewett MM, McCormick MA, Williamson JR, Kennedy B, Cravatt BF, Petrascheck M Translation attenuation by minocycline enhances longevity and proteostasis in old post-stress-responsive organisms. Elife. 2018; 7. 10.7554/eLife.40314. 30479271 PMC6257811

[255] Titorenko VI, Smith JJ, Szilard RK, Rachubinski RA Pex20p of the yeast Yarrowia lipolytica is required for the oligomerization of thiolase in the cytosol and for its targeting to the peroxisome. J Cell Biol. 1998; 142:403–20. 10.1083/jcb.142.2.403. 9679140 PMC2133052

[256] Madeo F, Fröhlich E, Fröhlich KU A yeast mutant showing diagnostic markers of early and late apoptosis. J Cell Biol. 1997; 139:729–34. 10.1083/jcb.139.3.729. 9348289 PMC2141703

