# Supplementary material for: Mechanisms by which PE21, an extract from the white willow Salix alba, delays chronological aging in budding yeast
Source: Oncotarget. 2019 Oct 8;10(56):5780–816. doi: 10.18632/oncotarget.27209 (PMC6791382; doi:10.18632/oncotarget.27209)
Supplement: Supplementary file 1 [file oncotarget-10-5780-s001.pdf]

Mechanisms by which PE21, an extract from the white willow *Salix alba*, delays chronological aging in budding yeast

SUPPLEMENTARY MATERIALS

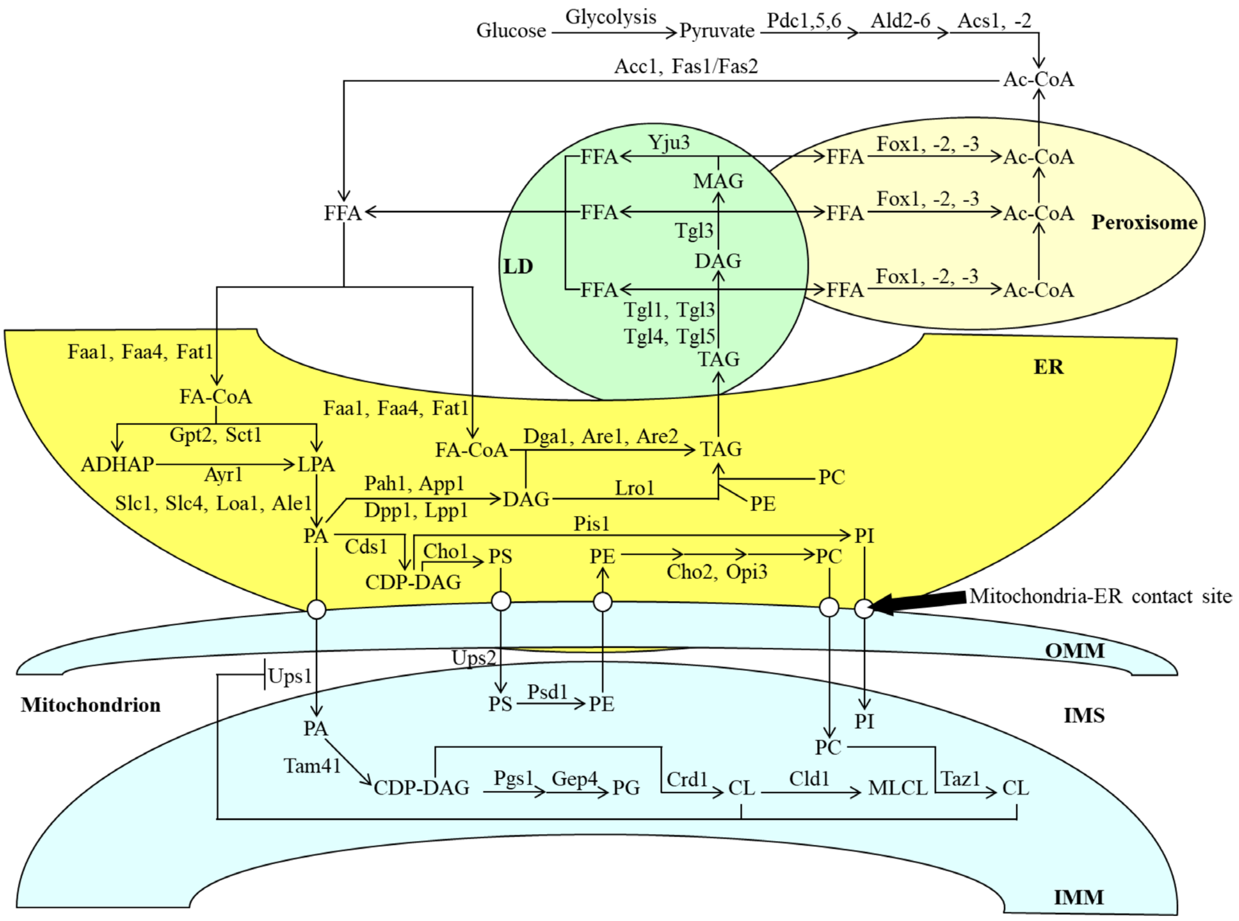

Supplementary Figure 1: The relative concentrations of various lipid classes in yeast cells depend on the metabolic and interorganellar transport processes that are catalyzed by enzymes residing in the cytosol, endoplasmic reticulum (ER), mitochondria, lipid droplets (LD) and peroxisomes. A T bar denotes a cardiolipin (CL)-dependent inhibition of phosphatidic acid (PA) transfer from the outer mitochondrial membrane (OMM) across the intermediate space (IMS) to the inner mitochondrial membrane (IMM). See text for more details. Other abbreviations: Acc1, acetyl-CoA carboxylase 1; Ac-CoA, acetyl-CoA; Acs1 and Acs2, acetyl CoA synthetases 1 and 2; ADHAP, acyl-dihydroxyacetone phosphate; Ale1, acyltransferase for lysophosphatidylethanolamine 1; Ald2-6, aldehyde dehydrogenases 2 to 6; App1, actin patch protein 1; Are1/2, acyl-coenzyme A: cholesterol acyl transferase-related enzymes 1 and 2; Ayr1, acyl-dihydroxyacetone-phosphate reductase 1; CDP, cytidine diphosphate; Cds1, CDP-diacylglycerol synthase 1; Cho1/2, choline requiring 1 and 2; CL, cardiolipin; Cld1, cardiolipin-specific deacylase 1; Crd1, cardiolipin synthase 1; DAG, diacylglycerol; Dga1, diacylglycerol acyltransferase 1; Dpp1, diacylglycerol pyrophosphate phosphatase 1; Faa1 and Faa4, fatty acid activation proteins 1 and 4; FA-CoA, fatty acyl-CoA ester; Fas1 and Fas2, fatty acid synthetases 1 and 2; Fat1, fatty acid transporter 1; FFA, free fatty acid; Fox1, Fox2 and Fox3, fatty acid oxidation proteins 1, 2 and 3; Gep4, genetic interactor of prohibitins protein 4; Gpt2, glycerol-3-phosphate acyltransferase; Loa1, lysophosphatidic acid: oleoyl-CoA acyltransferase 1; Lpp1, lipid phosphate phosphatase 1; LPA, lysophosphatidic acid; Lro1, lecithin cholesterol acyl transferase related open reading frame 1; MAG, monoacylglycerol; MLCL, monolysocardiolipin; Opi3, overproducer of inositol 3; PA, phosphatidic acid; Pah1, phosphatidic acid phosphohydrolase 1; PC, phosphatidylcholine; Pdc1, Pdc5 and Pdc6, pyruvate decarboxylases 1, 5 and 6; PE, phosphatidylethanolamine; PG, phosphatidylglycerol; Pgs1, phosphatidylglycerolphosphate synthase 1; PI, phosphatidylinositol; Pis1, phosphatidylinositol synthase 1; PS, phosphatidylserine; Psd1, phosphatidylserine decarboxylase 1; Sct1, suppressor of choline-transport mutants 1; Slc1 and Slc4, sphingolipid compensation proteins 1 and 4; Tam41, translocator assembly and maintenance protein 41; TAG, triacylglycerol; Taz1, tafazzin protein 1; Tgl1, Tgl3, Tgl4, Tgl5, triglyceride lipases 1, 3, 4 and 5; Ups1 and Ups2, unprocessed proteins 1 and 2; Yju3, monoglyceride lipase.

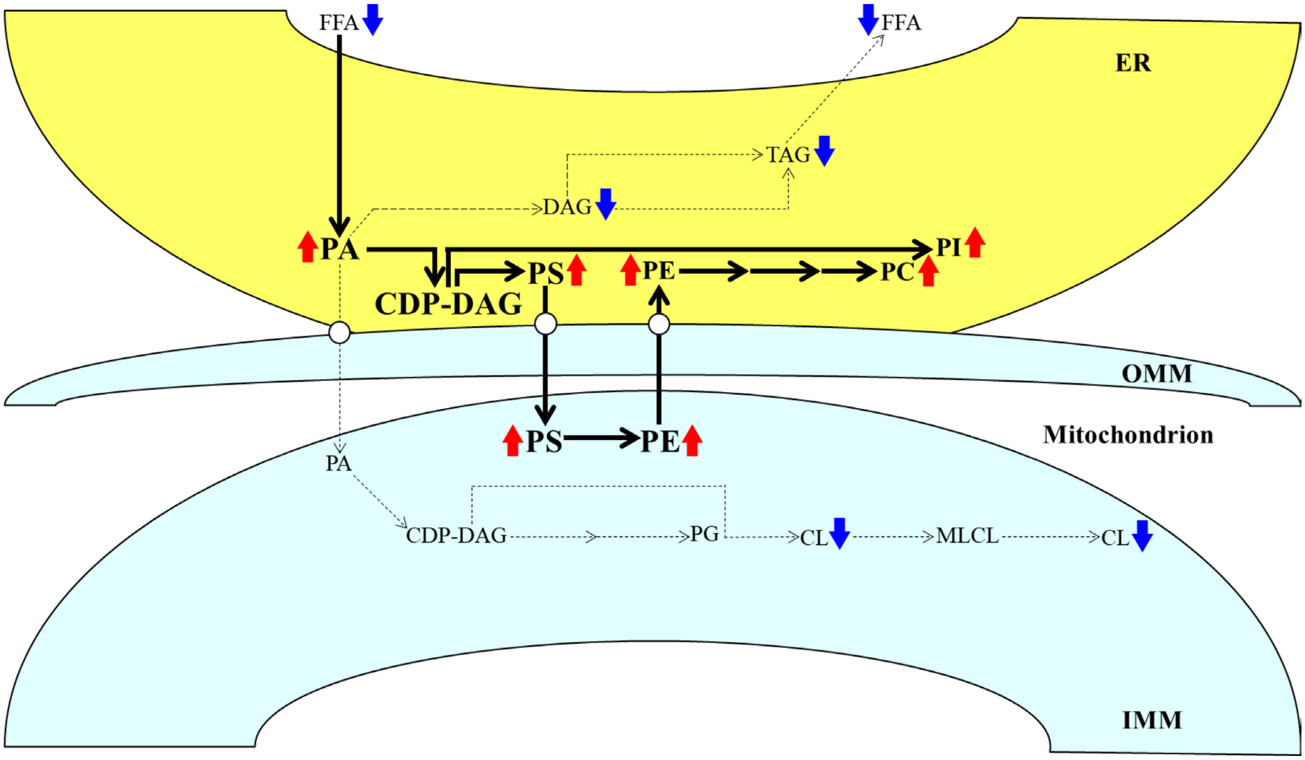

Supplementary Figure 2: A model for a PE21-dependent reorganization of lipid metabolism and transport in yeast cells. PE21 alters the efficiencies with which free fatty acids (FFA) and phosphatidic acid (PA) are included into the synthesis of other lipid classes in the endoplasmic reticulum (ER) and mitochondria. Arrows next to the names of lipid classes denote those of them whose concentrations are increased (red arrows) or decreased (blue arrows) in yeast cells cultured in the presence of PE21. The thickness of black arrows is proportional to the efficiency with which FFA and PA are included into the synthesis of other lipid classes. See text for more details. Other abbreviations: CL, cardiolipin; CDP, cytidine diphosphate; DAG, diacylglycerol; IMM, inner mitochondrial membrane; MLCL, monolysocardiolipin; OMM, outer mitochondrial membrane; PC, phosphatidylcholine; PE, phosphatidylethanolamine; PG, phosphatidylglycerol; PI, phosphatidylinositol; PS, phosphatidylserine; TAG, triacylglycerol.

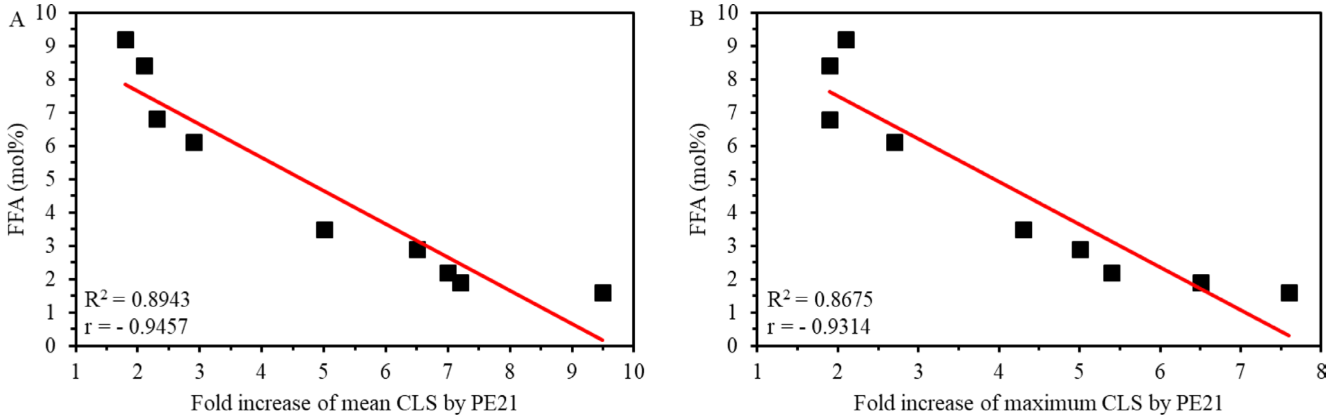

C

Genetic background	Fold increase of mean CLS by PE21	Fold increase of maximum CLS by PE21	FFA concentration (mol%)
tgl4Δ	9.5 ± 0.5	7.6 ± 0.6	1.6 ± 0.1
tgl3Δ	7.2 ± 0.3	6.5 ± 0.4	1.9 ± 0.2
tgl1Δ	7.0 ± 0.4	5.4 ± 0.2	2.2 ± 0.3
tgl5Δ	6.5 ± 0.3	5.0 ± 0.2	2.9 ± 0.1
WT	5.0 ± 0.4	4.3 ± 0.3	3.5 ± 0.4
faa1Δ	2.9 ± 0.2	2.7 ± 0.2	6.1 ± 0.7
faa4Δ	2.3 ± 0.2	1.9 ± 0.1	6.8 ± 0.6
slc1Δ	2.1 ± 0.1	1.9 ± 0.2	8.4 ± 0.9
ale1Δ	1.8 ± 0.1	2.1 ± 0.2	9.2 ± 1.2

Supplementary Figure 3: The efficiency with which PE21 extends yeast longevity inversely correlates with the intracellular concentration of FFA. WT cells and mutant cells that carry a single-gene-deletion mutation eliminating either Faa1, Faa4, Ale1, Slc1, Tgl1, Tgl3, Tgl4 or Tgl5 were cultured in the synthetic minimal YNB medium initially containing 2% glucose with 0.1% PE21. Survival curves shown in Figures 3A, 3F, 4A, 4F, 5A, 5F, 6A and 6F were used to calculate the folds of increase of the mean and maximum CLS by PE21 for the WT, *faa1D*, *faa4D*, *ale1D*, *slc1D*, *tgl1D*, *tgl3D*, *tgl4D* and *tgl5D* strains, as shown in Figures 3C, 3D, 3H, 3I, 4C, 4D, 4H, 4I, 5C, 5D, 5H, 5I, 6C, 6D, 6H and 6I. (**A**, **B**) Plots comparing the folds increase of mean (**A**) or maximum (**B**) CLS and the highest intracellular concentration of FFA (which was observed in WT and mutant cells recovered on day 3 of culturing with PE21). Different points show the data for WT, *faa1D*, *faa4D*, *ale1D*, *slc1D*, *tgl1D*, *tgl3D*, *tgl4D* or *tgl5D* cells. Linear trendlines and the R-squared values are displayed; these values demonstrate a good fit of the line to the data. The Pearson's correlation coefficient (r) values are also shown; because the r value less than - 0.9 is considered a very high negative correlation between the two variables, the fold increase of the mean (**A**) or maximum (**B**) CLS has a very high negative correlation with the intracellular concentration of FFA. (**C**) The experimental data used to create plots shown in (**A** and **B**). Genetic backgrounds of strains, the folds of increase of the mean and maximum CLS by PE21, and the highest concentration of FFA (which was observed in WT and mutant cells recovered on day 3 of culturing with PE21) are shown. Data are presented as means ± SEM (*n* = 4). Abbreviation: FFA, free fatty acids.

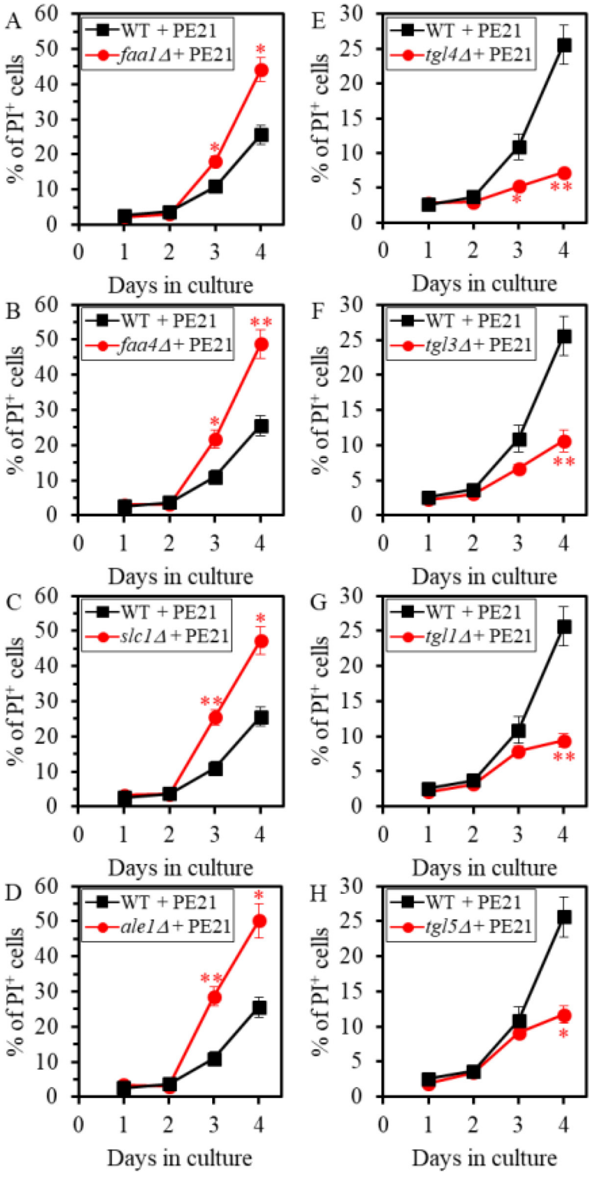

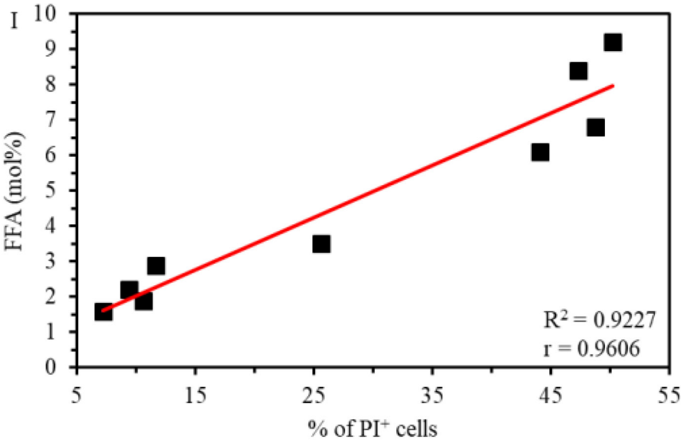

J

Genetic background	% of PI+ cells on Day 4	FFA concentration (mol%)
tgl4Δ	7.2 ± 0.6	1.6 ± 0.1
tgl3Δ	10.6 ± 1.6	1.9 ± 0.2
tgl1Δ	9.4 ± 0.9	2.2 ± 0.3
tgl5Δ	11.7 ± 1.2	2.9 ± 0.1
WT	25.6 ± 2.8	3.5 ± 0.4
faa1Δ	44.1 ± 3.4	6.1 ± 0.7
faa4Δ	48.8 ± 4.1	6.8 ± 0.6
slc1Δ	47.3 ± 3.9	8.4 ± 0.9
ale1Δ	50.2 ± 4.9	9.2 ± 1.2

Supplementary Figure 4: The percentage of cells undergoing necrotic death directly correlates with FFA concentration in the yeast cell. WT cells and mutant cells carrying a single-gene-deletion mutation eliminating either Faa1, Faa4, Ale1, Slc1, Tgl1, Tgl3, Tgl4 or Tgl5 were cultured in the synthetic minimal YNB medium initially containing 2% glucose with 0.1% PE21. (**A**–**H**) Cells recovered on different days of culturing were stained with propidium iodide (PI) as described in Materials and Methods. PI positive staining identifies cells that are permeable to PI because their plasma membranes have been damaged. Such loss of plasma membrane integrity is a hallmark event of necrotic cell death. Percentage of cells exhibiting PI positive staining is shown. Data are presented as means ± SEM (n = 3; $^{*}p < 0.05$; $^{**}p < 0.01$). Data for the WT strain cultured with PE21 are replicated in graph B of Figure 7. (**I**) Plot comparing the maximum percentage of cells exhibiting PI positive staining (which was observed in WT and mutant cells recovered on day 4 of culturing with PE21) and the highest intracellular concentration of FFA (which was observed in WT and mutant cells recovered on day 3 of culturing with PE21). Different points show the data for WT, *faa1D*, *faa4D*, *ale1D*, *slc1D*, *tgl1D*, *tgl3D*, *tgl4D* or *tgl5D* cells. Linear trendline and the R-squared value are displayed; the R-squared value demonstrates a good fit of the line to the data. The Pearson's correlation coefficient (r) value is also shown. Because the r value more than 0.9 is considered a very high positive correlation between the two variables, the percentage of cells exhibiting PI positive staining has a very high positive correlation with the intracellular concentration of FFA. (**J**) The experimental data used to create the plot shown in (**I**). Genetic backgrounds of strains, the percentage of cells exhibiting PI positive staining on day 4 of culturing with PE21, and the highest intracellular concentration of FFA observed on day 3 of culturing with PE21 are shown. Data are presented as means ± SEM (n = 3). Abbreviation: FFA, free fatty acids.

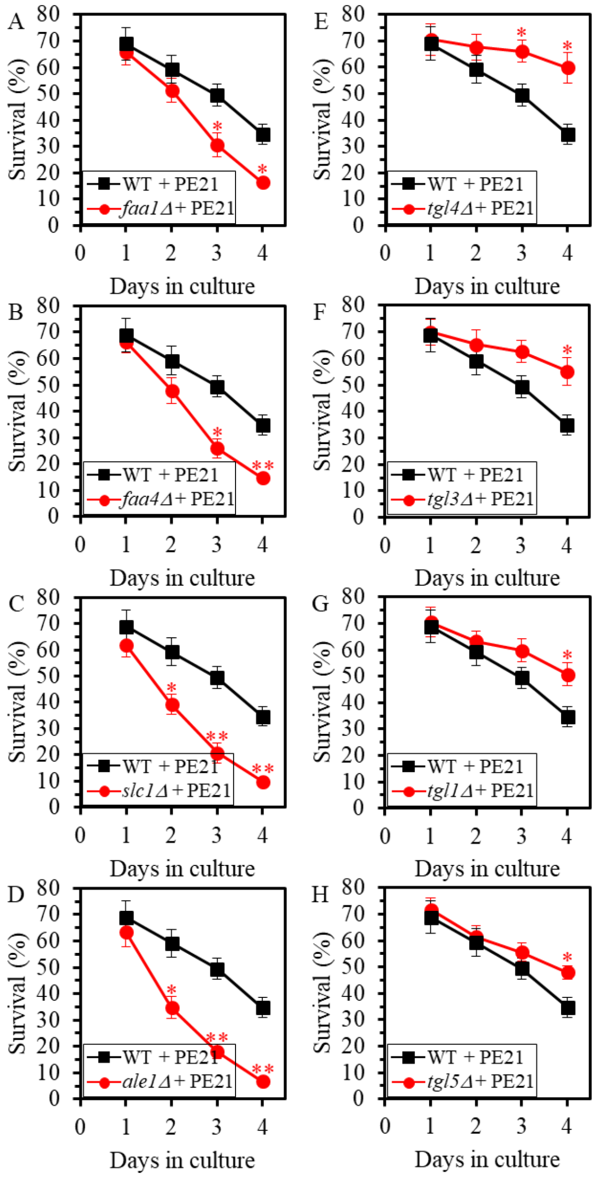

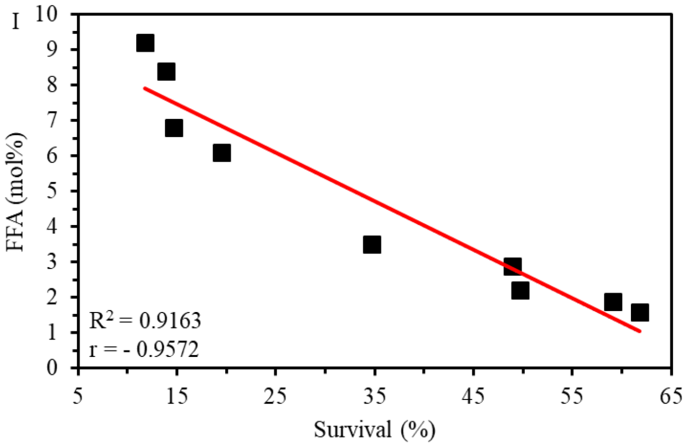

J

Genetic background	Survival (%) on Day 4	FFA concentration (mol%)
tgl4Δ	59.8 ± 5.6	1.6 ± 0.1
tgl3Δ	55.1 ± 5.1	1.9 ± 0.2
tgl1Δ	50.7 ± 4.3	2.2 ± 0.3
tgl5Δ	47.9 ± 2.7	2.9 ± 0.1
WT	34.7 ± 3.8	3.5 ± 0.4
faa1Δ	16.5 ± 1.4	6.1 ± 0.7
faa4Δ	14.7 ± 1.9	6.8 ± 0.6
slc1Δ	9.9 ± 1.1	8.4 ± 0.9
ale1Δ	6.8 ± 0.9	9.2 ± 1.2

Supplementary Figure 5: The resistance of yeast cells to liponecrotic RCD inversely correlates with FFA concentration in the yeast cell. WT cells and mutant cells carrying a single-gene-deletion mutation eliminating either Faa1, Faa4, Ale1, Slc1, Tgl1, Tgl3, Tgl4 or Tgl5 were cultured in the synthetic minimal YNB medium initially containing 2% glucose with 0.1% PE21. (**A**–**H**) Clonogenic survival of cells recovered on different days of culturing and then treated for 2 h with 0.1 mM POA (a monounsaturated form of FFA) to elicit a liponecrotic mode of RCD as described in Materials and Methods. Data are presented as means ± SEM (n = 3; $^{*}p < 0.05$; $^{**}p < 0.01$). Data for the WT strain cultured with PE21 are replicated in graph (**C**) of Figure 7. (**I**) Plot comparing the minimum percentage of clonogenic survival of POA-treated cells (which was observed in WT and mutant cells that were recovered on day 4 of culturing with PE21 and then treated with POA) and the highest intracellular concentration of FFA (which was observed in WT and mutant cells recovered on day 3 of culturing with PE21). Different points show the data for WT, *faa1D*, *faa4D*, *ale1D*, *slc1D*, *tgl1D*, *tgl3D*, *tgl4D* or *tgl5D* cells. Linear trendline and the R-squared value are displayed; the R-squared value demonstrates a good fit of the line to the data. The Pearson's correlation coefficient (r) value is also shown. Because the r value less than - 0.9 is considered a very high negative correlation between the two variables, the resistance of yeast cells to liponecrotic RCD has a very high negative correlation with FFA concentration in the yeast cell. (**J**) The experimental data used to create the plot shown in (**I**). Genetic backgrounds of strains, the minimum percentage of clonogenic survival of cells that were recovered on day 4 of culturing with PE21 and then treated with POA, and the highest intracellular concentration of FFA observed on day 3 of culturing with PE21 are shown. Data are presented as means ± SEM (n = 3). Abbreviation: FFA, free fatty acids.

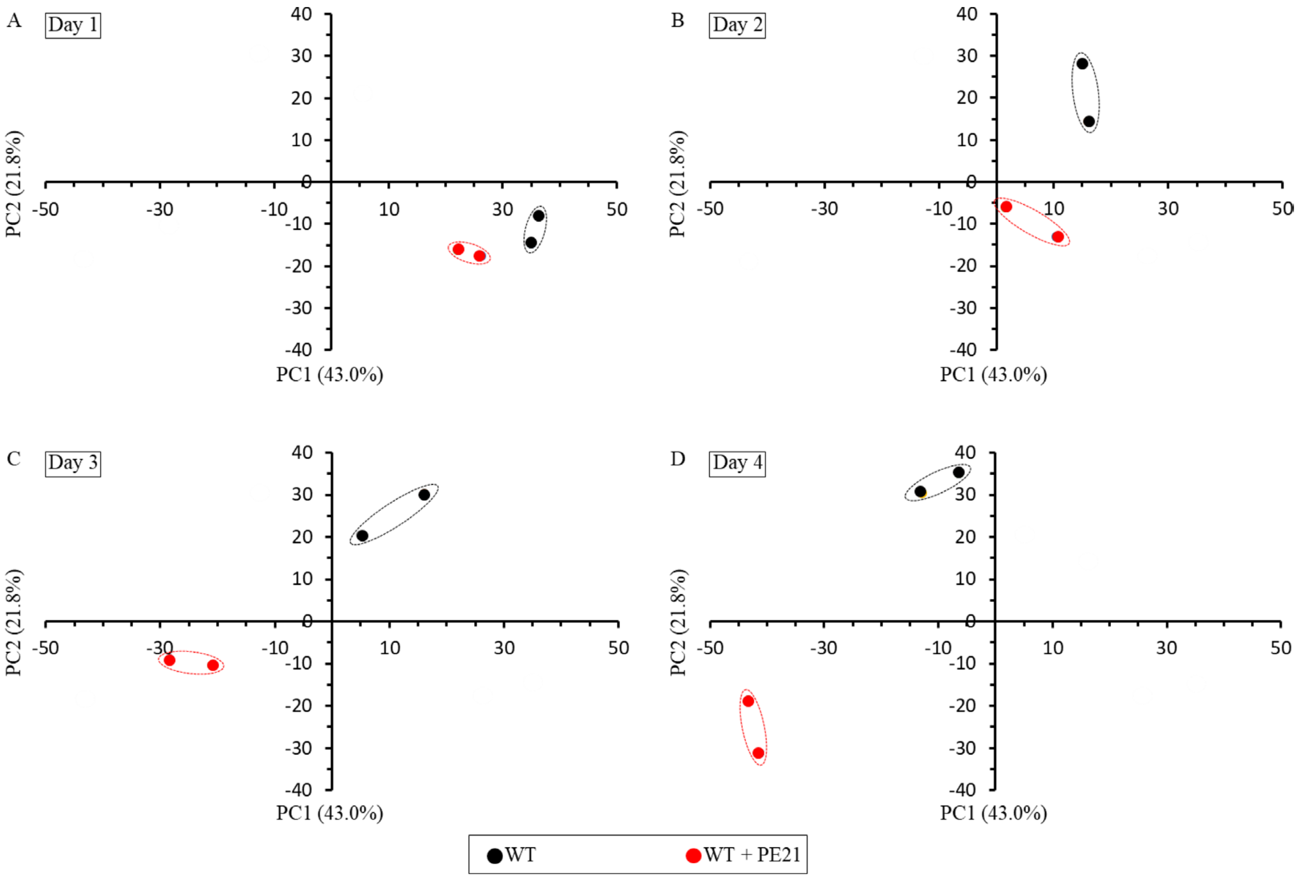

Supplementary Figure 6: Principal component analysis (PCA) for the comparison of cellular proteins identified in yeast cultured in the presence of PE21 or in its absence. WT cells were cultured in the synthetic minimal YNB medium initially containing 2% glucose with 0.1% PE21 or without it. Cells were recovered on days 1, 2, 3 and 4 of culturing. Mass spectrometry-based identification and quantitation of proteins recovered from these cells was performed as described in Materials and Methods. (**A**–**D**) PCA was performed for proteins identified in cells that were cultured with or without PE21 and recovered on day 1 (**A**), day 2 (**B**), day 3 (**C**) or day 4 (**D**) of culturing. Data of 2 independent experiments, each being depicted by a red or black dot, are presented. Partitional clustering of quantitative data was performed using the *k*-means clustering algorithm.

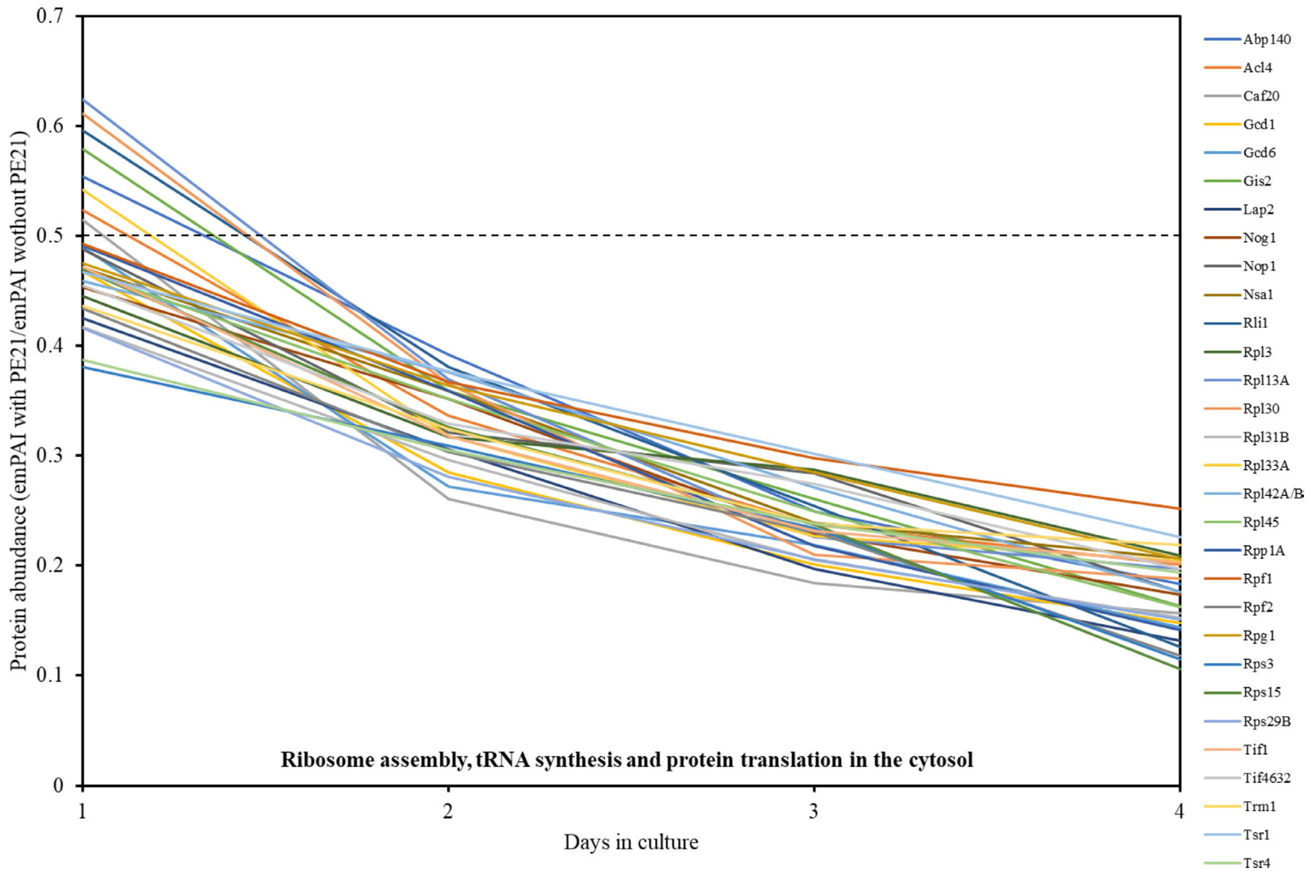

Supplementary Figure 7: PE21 decreases the abundance of cellular proteins known to be downregulated during the UPR^{ER} response in yeast. These proteins are involved in ribosome assembly, tRNA synthesis and protein translation in the cytosol. WT cells were cultured in the synthetic minimal YNB medium initially containing 2% glucose with 0.1% PE21 or without it. Cells were recovered on days 1, 2, 3 and 4 of culturing. Mass spectrometry-based identification and quantitation of proteins recovered from these cells, and the calculation of the relative abundance of cellular proteins in a pair of analyzed datasets (i.e. in the datasets of age-matched WT cells cultured with or without PE21), were performed as described in Materials and Methods. Relative levels of proteins in WT cells cultured with PE21 (fold difference relative to those in WT cells cultured without PE21) are shown. The 2-fold decrease in the ratio "protein abundance with PE21/protein abundance without PE21" is shown by a dotted line. Data are presented as mean values of 2 independent experiments. Abbreviation: emPAI, the exponentially modified protein abundance index, a measure of the relative abundance of cellular proteins in a pair of analyzed datasets.

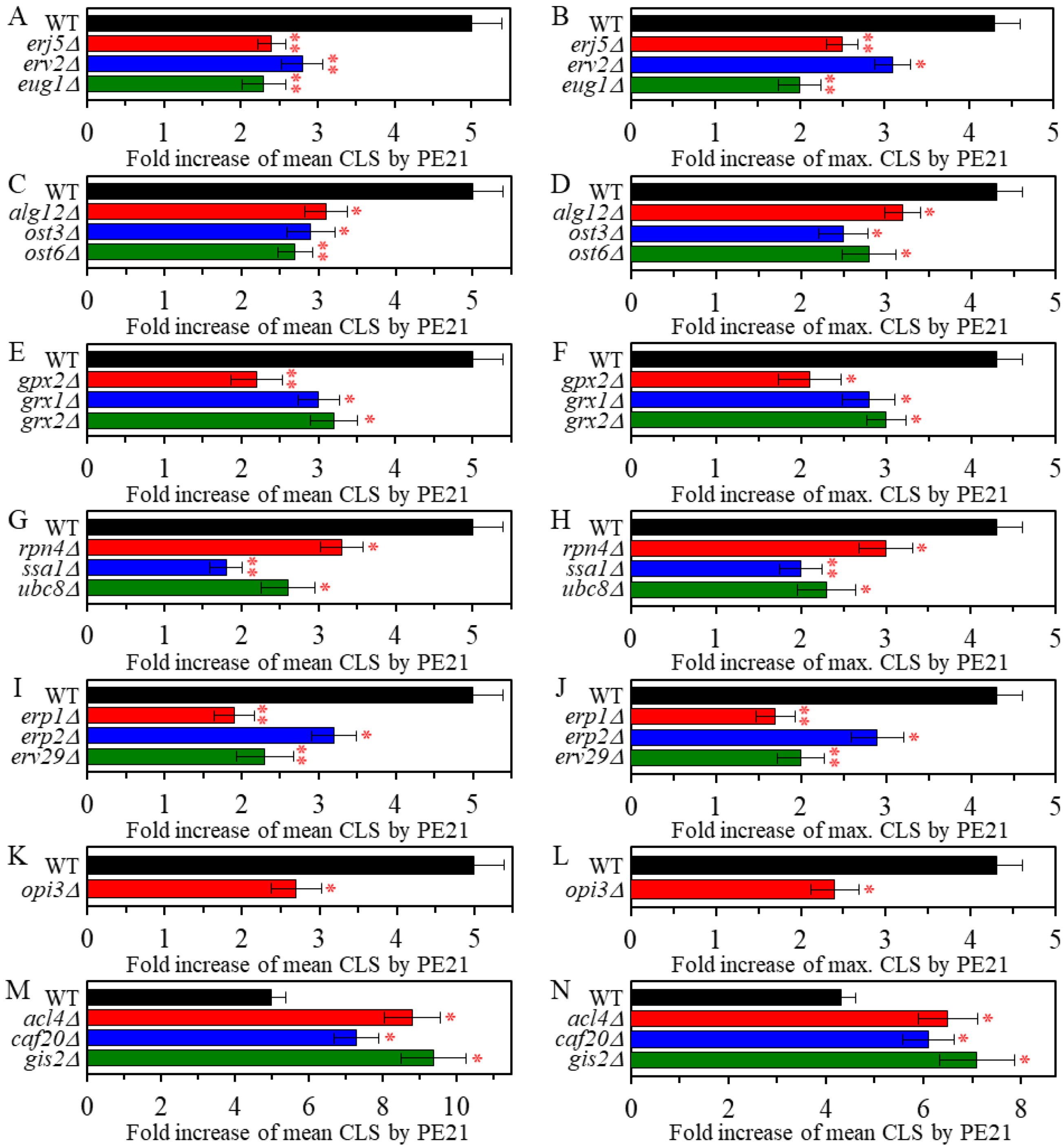

Supplementary Figure 8: Single-gene-deletion mutations eliminating proteins that are upregulated by both PE21 and UPR^{ER} stimuli decrease the efficiency with which PE21 extends yeast longevity, whereas single-gene-deletion mutations eliminating proteins that are downregulated by both PE21 and UPR^{ER} stimuli increase such efficiency. WT cells and mutant cells carrying a single-gene-deletion mutation eliminating a protein upregulated or downregulated by both PE21 and UPR^{ER} stimuli were cultured in the synthetic minimal YNB medium initially containing 2% glucose with 0.1% PE21 or without it. Survival curves of chronologically aging WT and mutant strains were used to calculate the fold of increase of the mean (**A**, **C**, **E**, **G**, **I**, **K**, **M**) and maximum (**B**, **D**, **F**, **H**, **J**, **L**, **N**) CLS by PE21 for the WT and mutant strains. Data are presented as means ± SEM ($n = 3$; $^{*}p < 0.05$; $^{**}p < 0.01$). Data for the WT strain are replicated in graphs of (**A**–**N**) and Figures 3C, 3D, 3H, 3I, 4C, 4D, 4H, 4I, 5C, 5D, 5H, 5I, 6C, 6D, 6H, 6I, 10I, 10J, 11G, 11H, 14I, 14J, 15I, 15J, and Supplementary Figure 10A–10N.

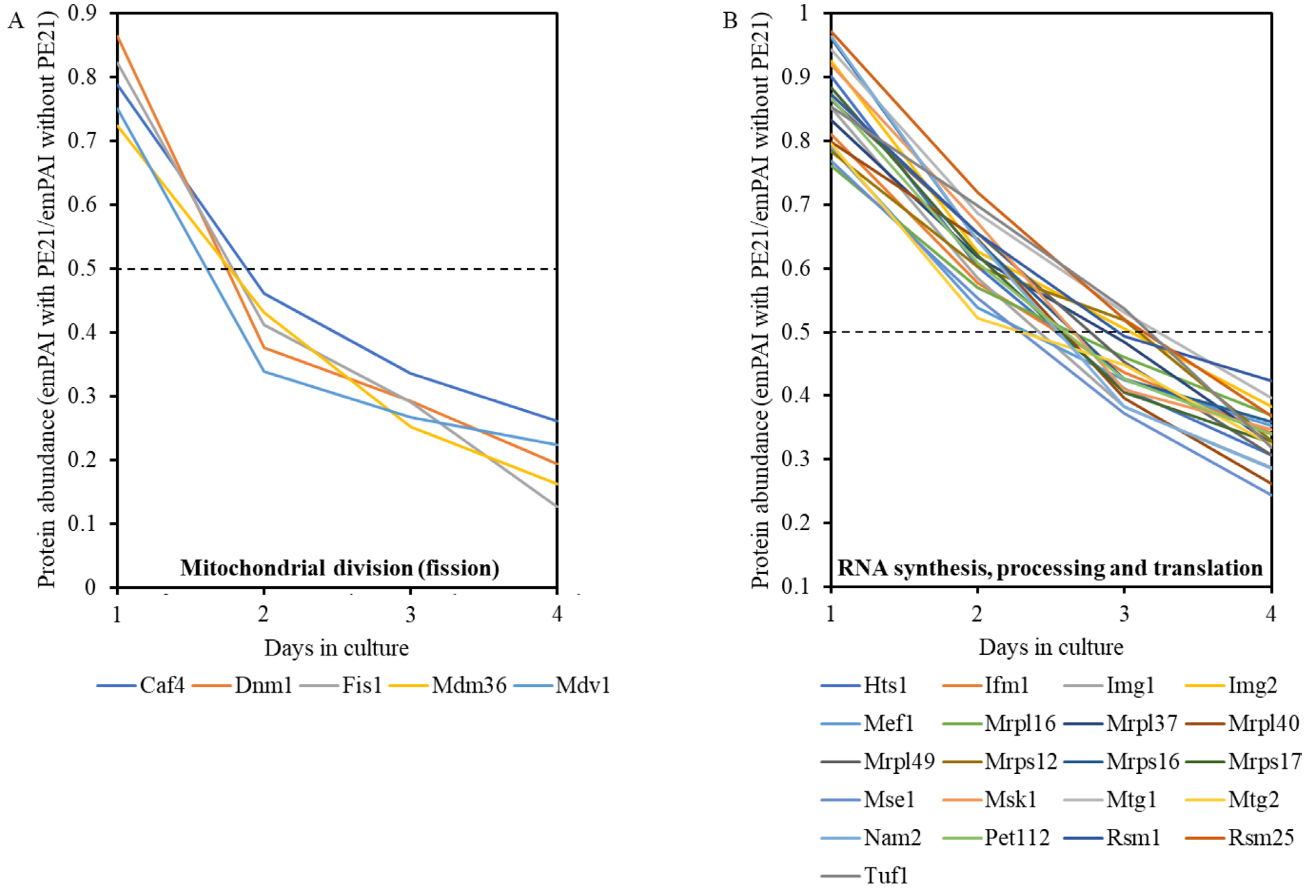

Supplementary Figure 9: PE21 decreases the abundance of two classes of mitochondrial proteins. WT cells were cultured in the synthetic minimal YNB medium initially containing 2% glucose with 0.1% PE21 or without it. Cells were recovered on days 1, 2, 3 and 4 of culturing. Mass spectrometry-based identification and quantitation of proteins recovered from these cells, and the calculation of the relative abundance of cellular proteins in a pair of analyzed datasets (i.e. in the datasets of age-matched WT cells cultured with or without PE21), were performed as described in Materials and Methods. Relative levels of proteins in WT cells cultured with PE21 (fold difference relative to those in WT cells cultured without PE21) are shown. These proteins include the following ones: components of the mitochondrial division (fission) machinery (**A**), and proteins that catalyze RNA synthesis, processing and translation within mitochondria (**B**). The 2-fold decrease in the ratio "protein abundance with PE21/protein abundance without PE21" is shown by a dotted line. Data are presented as mean values of 2 independent experiments. Abbreviation: emPAI, the exponentially modified protein abundance index, a measure of the relative abundance of cellular proteins in a pair of analyzed datasets.

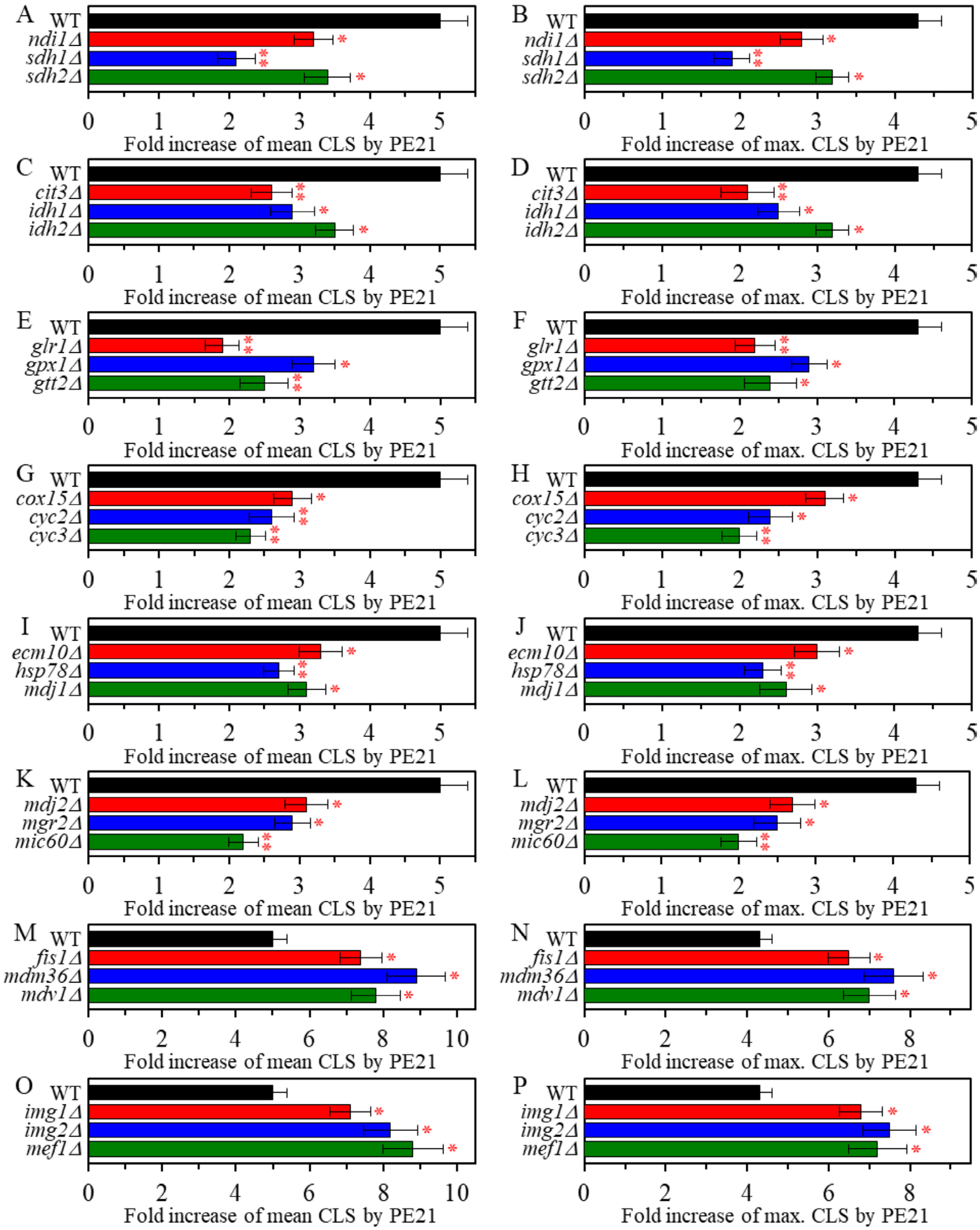

Supplementary Figure 10: Single-gene-deletion mutations eliminating mitochondrial proteins that are upregulated by PE21 decrease the geroprotective efficiency of PE21, while single-gene-deletion mutations eliminating mitochondrial proteins that are downregulated by PE21 increases such efficiency. WT cells and mutant cells carrying a single-gene-deletion mutation eliminating a mitochondrial protein upregulated or downregulated by PE21 were cultured in the synthetic minimal YNB medium initially containing 2% glucose with 0.1% PE21 or without it. Survival curves of chronologically aging WT and mutant strains were used to calculate the fold of increase of the mean (**A**, **C**, **E**, **G**, **I**, **K**, **M**, **O**) and maximum (**B**, **D**, **F**, **H**, **J**, **L**, **N**, **P**) CLS by PE21 for the WT and mutant strains. Data are presented as means ± SEM (n = 3; $^{*}p < 0.05$; $^{**}p < 0.01$). Data for the WT strain are replicated in graphs of (**A**–**P**) and Figures 3C, 3D, 3H, 3I, 4C, 4D, 4H, 4I, 5C, 5D, 5H, 5I, 6C, 6D, 6H, 6I, 10I, 10J, 11G 11H, 14I, 14J, 15I 15J, and Supplementary Figure 8A–8N.
